# Synthesis and Antibacterial Study of Novel Harmine Derivatives and Tetrahydro-β-Carboline Derivatives In Vitro

**DOI:** 10.3390/molecules27092888

**Published:** 2022-04-30

**Authors:** Yan Liang, Tianzeng Song, Bingmei He, Lei Tang, Deshun Zhou, Dian He

**Affiliations:** 1 School of Pharmacy, Lanzhou University, West Donggang Road No. 199, Lanzhou 730000, China; liangy18@lzu.edu.cn (Y.L.); lishangwei6636@sina.com (L.T.); 2 Institute of Animal Science, Tibet Academy of Agricultural & Animal Husbandry Science, Lhasa 850009, China; songtianzeng123@sina.com (T.S.); kgchbm126com@126.com (B.H.); 3 Key Laboratory of Veterinary Pharmaceutical Development, Ministry of Agriculture and Rural Affairs, Lanzhou Institute of Husbandry and Pharmaceutical Sciences of Chinese Academy of Agriculture Sciences, No. 335, Qilihe District, Lanzhou 730050, China

**Keywords:** single crystal diffraction, chemical synthesis, bacteriostatic experiment, molecular docking

## Abstract

Dairy mastitis is a disease of dairy cattle caused by a variety of pathogenic microorganisms which has biought huge economic losses aused huge economic losses to the world. In this paper, Harmine derivatives and tetrahydro-β-carboline derivatives synthesized by the splice method are shown to have a good inhibitory effect on the pathogenic bacteria of dairy mastitis. The results of a bacteriostatic test on pathogenic bacteria of dairy cow mastitis (*S. dysgalactiae*, *S. pyogenes*, *B. subtilis* and *P. vulgaris*) showed that compound **7l** had the best bacteriostatic effect on *Streptococcus dysgalactiae*, with a mic value of 43.7 μ g/mL. When the concentration of **7l** was 1 × MIC and 2 × MIC, it had a significant inhibitory effect on *Streptococcus dysgalactiae*, and there was almost no growth of *Streptococcus dysgalactiae* at 4 × MIC. The binding properties of target compound **7l** to *amine oxidase [flavin-containing] A* protein were simulated by the molecular docking technique. The ligand **7l** achieved strong binding with the receptor through three hydrogen bonds. The hydrogen bonds were amino acid residues thr-52, arg-51 and ser-24, which are the main force for the compound to bind to active sites.

## 1. Introduction

**Figure 1:**
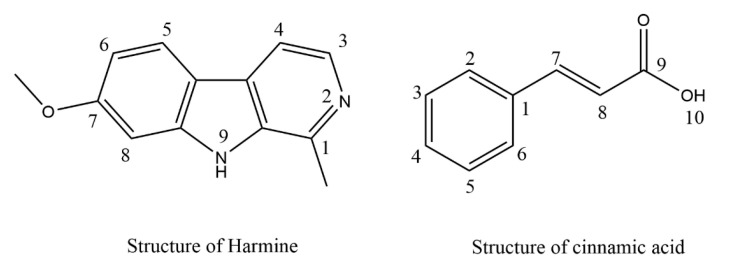
Structure of Harmine and cinnamic acid.

Harmel is a plant of the genus Pederma of Tribulus teristaceae, growing in arid areas [1]. Harmine is the main active ingredient of Pederma teristaceae [2]; The structure is shown in Figure 1. It has antibacterial properties and protects the seeds from bacterial invasion. Harmine has abundant natural distribution and extensive pharmacological activities, i.e., anti-tumor, anti-inflammatory, antioxidant and other effects. Additionally, its excellent antibacterial effect has attracted extensive attention from researchers [3]. Structural modification of harmine is expected to improve its antibacterial activity. Cinnamic acid is isolated from cinnamon or benzoin resin and has the characteristics of low molecular weight and low price. Harmine and cinnamic acid are both plant extracts with certain bacteriostatic effects. Compounds **7l** and **7h** were obtained by concatenating the cinnamate moiety at the harmine N9 site. Compounds **11b**, **11f** and **11g** are obtained by linking different benzene ring groups at N2 site of tetrahydro-β-carboline through ester bonds. The structure of tetrahydro-β-carboline derivative is similar to that of the Harmine derivative. The spatial structure of the compound was obtained by single crystal diffraction [4,5]. This is a common chemical modification method to connect two compounds to produce new compounds. In this paper, target compound **7l** is shown to have significantly improved antibacterial performance, and notably, enhanced inhibition of *Streptococcus agalactiae*. In recent years, the topic non-antibiotic drugs of cow mastitis has received a great deal of attention. In this paper, *S. dysgalactiae, S. pyogenes, B. subtilis* and *P. vulgaris* was selected as the experimental bacteria to study the inhibitory effect of the target compound **7l** on the pathogenic bacteria of cow mastitis. It provides a theoretical basis for the study of Harmine derivatives and target compound **7l**. 

## 2. Results

### 2.1. Minimum Inhibitory Concentration Test

A microplate dilution method was used to study the inhibitory effect of compounds **7l**, **7h**, **11b**, **11f** and **11g** on Gram-positive bacteria (*S. dysgalactiae, S. pyogenes, B. subtilis*) and gram-negative bacteria (*P. vulgaris*). Harmine and cinnamic acid are used as control drugs. And the results were shown in Table 1. Compounds **7l**, **7h**, **11b**, **11f** and **11g** had a good inhibitory effect on both Gram-positive and Gram-negative bacteria, and **7l** had the best inhibitory effect on *S. dysgalactiae.*

### 2.2. Results of Bactericidal Time-Kill Kinetics

**Figure 2:**
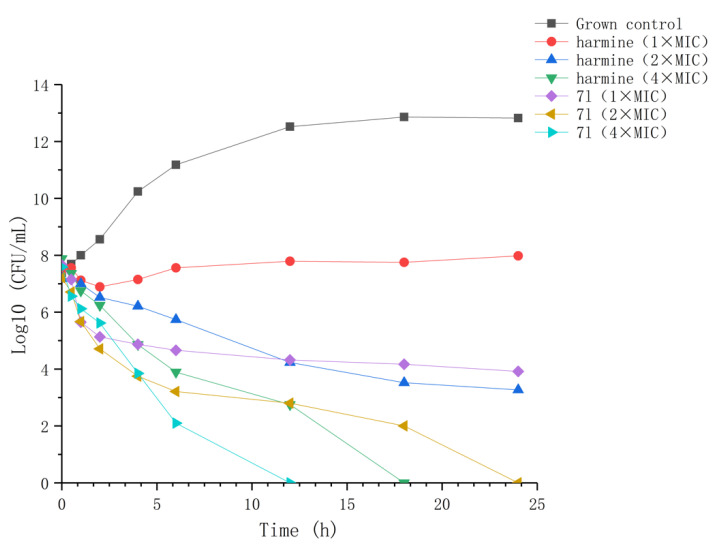
Time-kill kinetics of compound **7l** at 1 ×, 2 ×, and 4 × MIC against *S. dysgalactiae* (ATCC 35666).

The results of bactericidal time-kill kinetics were shown in Figure 2. Accord to the effect of Harmine and **7l** on *Streptococcus dysgalactiae*, the growth inhibition of 1 × MIC in the Harmine group was shown. The growth inhibition was significant in the 2 × MIC group, and no growth was found in the 4 × MIC group at 10 h. In the **7l** group, 1 × MIC was significantly inhibited, and 2 × MIC and 4 × MIC did not grow at 12 h and 6 h, respectively. Therefore, compound **7l** has a stronger inhibitory effect on *Streptococcus dysgalactiae* than Harmine. 

### 2.3. Synthesis and Characterization

**Scheme 1:**
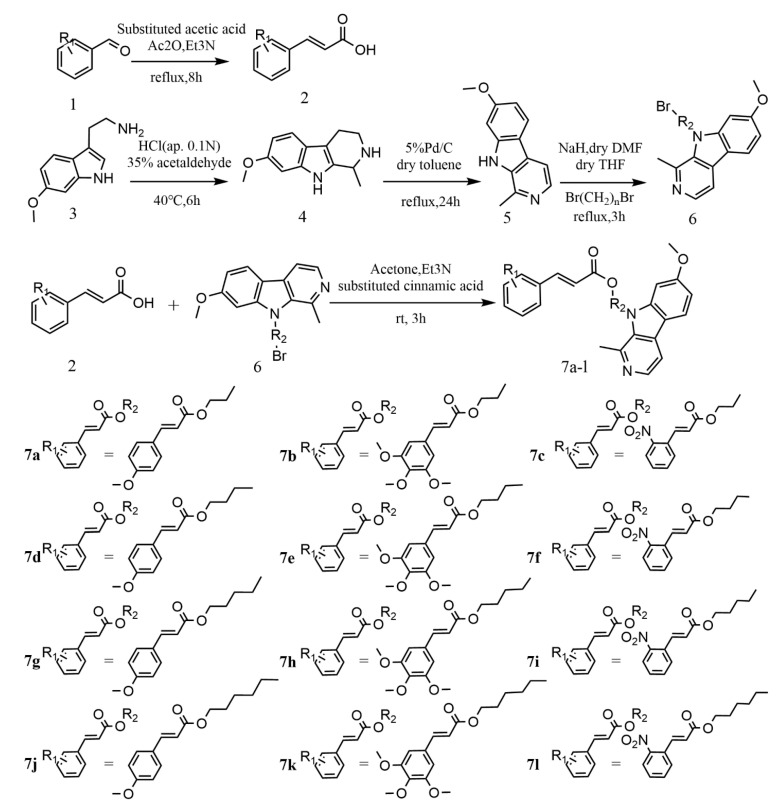
Synthesis of Harmine derivative **7a**–**7l**.

The synthesis of **7l** and its intermediates is described in Scheme 1. After Pictet-Spengler reaction, 6-methoxytryptamine forms β-carboline framework under acidic conditions, and is dehydrogenated under Pd/C catalysis to obtain Harmine. After Perkin reaction, substituted benzaldehyde and substituted acetic acid produce substituted cinnamic acid. The Harmine N9 site reacts with dibromoalkane in nucleophilic substitution. The nucleophilic substitution reaction between the product and substituted cinnamic acid is the key of the whole process.

**Scheme 2:**
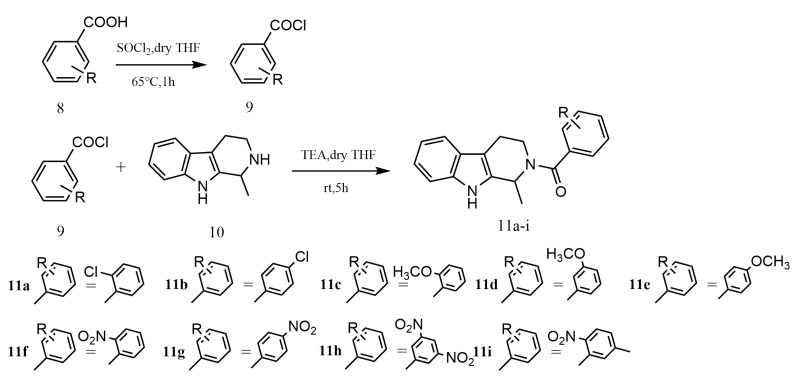
Synthesis of tetrahydro -β-carboline derivatives **11a**–**11i**.

Tetrahydro-β-carboline derivatives and their intermediates are synthesized in Scheme 2. The formation of the amide bond generally occurs via the condensation reagent method; however, as the condensation reagent method progresses too quickly, this experiment used the acyl halide method, which is relatively mild. In this setting, commonly used acylation reagents are SOCl_2_ and COCl_2_. First, the corresponding carboxylic acid reacts with its acylation reagent to produce the corresponding acyl chloride; it then reacts with the amino group to form an amide bond while the acyl halide method synthesizes the amide bond. The purpose of this is to improve the controllability of the whole reaction process.

### 2.4. Crystal Structure Description of the Compound

**Figure 3:**
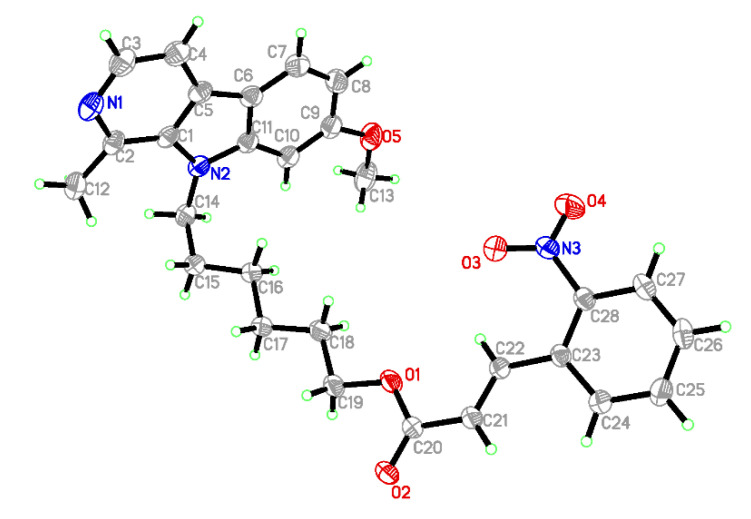
Spatial structure of compound **7l**.

**Figure 4:**
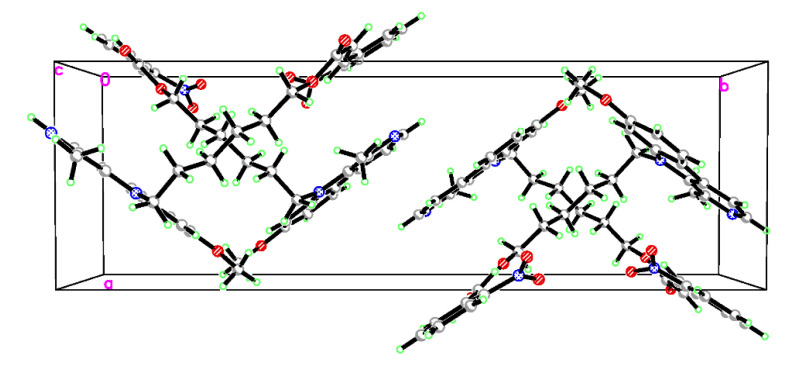
Stacking diagram of compound **7l**.

Crystal data of **7l** compound were obtained by X-ray single crystal diffraction. The molecular formula of the compound is C_28_H_29_N_3_O_5_. Detailed: monoclinic P 2_1_/*c* (no.14) a = 8.7050(11) Å b = 27.138(3) Å c = 10.4121(14) Å V = 2457.9(5) Å^3^ Z = 4 Rgt(F) = 0.0485 wRref(F2) = 0.1067. The crystal temperature remains 296(2) K. Detailed crystallographic data and structural refinement parameters are shown in Table 2. Draw spatial structure diagram (Figure 3) and packing diagram (Figure 4) according to compound geometric parameters of 7l.

The crystals of **7l** is a colorless, transparent and shiny block particles. The crystal size is 0.3 mm × 0.25 mm × 0.25 mm. The data were calculated by the least square method.

The molecule is not coplanar, according to the torsion angles C21-C22-C23-C28(-165.49°) and C21-C22-C23-C24(17.8°). The angle between Cinnamic acid nuclear plane C23-C28 and Harmine nuclear plane C1-C11 is 68.075(33), The C20-O1 bond (1.3252 Å) is shorter than an ordinary C19-O1 bond (1.4517 Å) but longer than a C20-O2 bond (1.1979 Å) due to p-π conjugation effect. The sum of the angles of O2-C20-O1(123.15°), O2-C20-C21(123.79°) and O1-C20-C21(113.06°) is 360°, indicating the sp^2^ hybridization state of the C(22) atom.

The cinnamic acid benzene part of target compound **7l** is parallel to the harmine benzene part of another **7l** molecule, and the distance between them is 3.6687 Å, which is conducive to a stable structure between molecules. According to the crystal structure analysis of the target compound, the plane where C1-C11 and C23-C28 are located is an immutable region, and C14-C22 is a single chain with a long distance which can be torsional and folded. This allows target compound **7l** to enter the active pocket and generate hydrogen bonds and other secondary bonds with the receptor.

### 2.5. Molecular Docking Results

**Figure 5:**
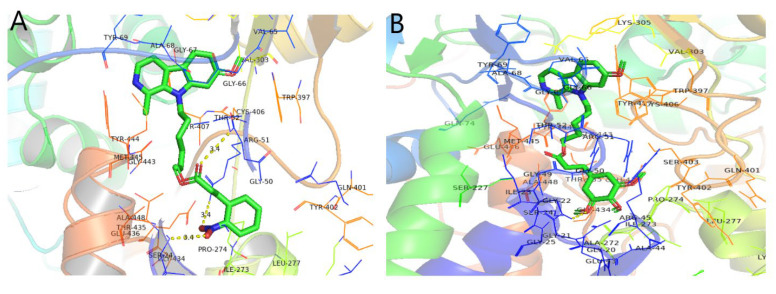
Schematic diagram of target compound **7l** (**A**) and **7h** (**B**) binding to *Amine oxidase [flavin-containing] A* protein.

The protein receptor PDBID: 2Y5Z is matched and docked with ligands **7l** and **7h** through AutoDock Vina software. The results showed that **7l** was bound to amino acid residues Thr-52, Arg-51 and Ser-24 of *Amine oxidase [flavin-containing] A* protein by hydrogen bonds, and the bond lengths were 3.4 Å, 3.4 Å and 3.4 Å respectively [6]. Additionally, **7h** binds to the Gly-22 amino acid residue of *Amine oxidase [flavin-containing] A* receptor and produces a 2.3 Å long hydrogen bond at Gly-22. Which is the main force for molecules to enter the active site. The results are shown in Figure 5. The **7l** crystal structure was compared with the ligand structure obtained by docking. The C19-O1-C20 angle of the ligand structure is larger than that of **7l**. The included angle of the target compound C19-O1-C20 is 117.00 (11). The plane of C1-C11 and C23-C28 is an immutable region, and the structure has not changed. The single chain region of C14-C22 is twisted and folded, so that the oxygen atom atO2 position forms a hydrogen bond with Thr-52. After molecular docking, the molecular energy is −9.6 kcal/mol. The ligand structure of **7h** is compared with the crystal structure. The Angle of crystal structure C12-O5-C13 is 115.747°, that of C1-C6 and C18-C28 is 14.2°, that of the ligand structure C12-O5-C13 is 109.8°, and the dihedral Angle of C1-C6 and C18-C28 is 165.5°. The regional structure of the indole ring and benzene ring did not change, and the molecular energy after docking was −7.9 kcal/mol. According to the data recorded in the PDB database, a combination of *Amine oxidase [flavin-containing] A* receptor and Harmine does not produce a hydrogen bond. Target compounds **7l** and **7h** have good binding characteristics with *Amine oxidase [flavin-containing] A*, and their strength is greater than that of Harmine, Detailed crystal data are shown in Tables S1–S30 of the supplementary document.

**Figure 6:**
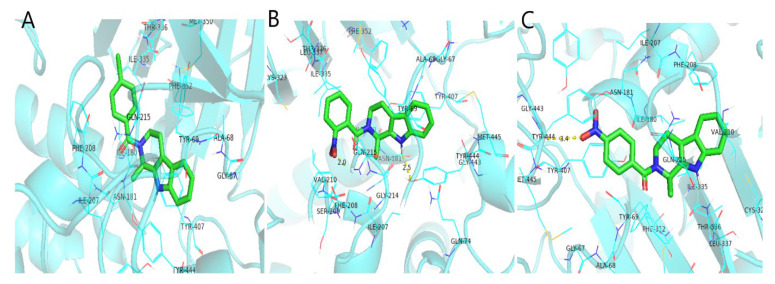
Schematic diagram of compounds **11b** (**A**), **11f** (**B**) and **11g** (**C**) binding to *Amine oxidase [flavin-containing] A* protein.

The protein receptor PDBID:2Y5Z was combined with ligands **11b**, **11f** and **11g** in a flexible docking manner. Ligands have different binding properties at receptor binding sites. The binding diagram is preferentially drawn with the conformation with the lowest binding energy, as shown in Figure 6. Notably, **11b** has no hydrogen bond when entering the active site, and the molecular energy after docking is −8.1 kcal/mol. Additionally, **11F** binds to the active site of *Amine oxidase [flavin containing] A* protein by two hydrogen bonds, producing hydrogen bonds with lengths of 2.0 Å and 2.5 Å at the amino acid residues of Gln-215 and Tyr-444, respectively. The molecular energy after binding is −8.7kcal/mol. The N2-C13-C19 angle of the ligand structure of **11f** is the same as that of the crystal structure, which is 119.9°. The dihedral angle between the C1-C7 plane and C14-C19 plane of the crystal structure is 40.3°, and the dihedral angle between the C1-C7 plane and C14-C19 plane of the ligand structure is smaller than that of crystal structure, which facilitates the formation of the hydrogen bond between **11f** and Gln-215. 

The binding site of **11g** entering the active pocket is similar to that of **11f**. It is also connected in the form of a hydrogen bond with Tyr-444 amino acid residue. The hydrogen bond length is 3.5 Å and the molecular energy after binding is −7.7kcal/mol. The N2-C11-C12 angle of the ligand structure of **11g** is the same as that of the crystal structure of **11g**: 120.0°. The C1-C8 region of the crystal structure and ligand structure is the same as that of the C12-C17 region, and there is no change. The torsion angle of N2-C11-C12-C13 and the dihedral angle between the C1-C6 plane and C12-C17 plane of the ligand structure are larger than those of the crystal structure. The torsion angle of N2-C11-C12-C13 is 59.7°, and the dihedral angle formed by the C1-C6 plane and C12-C17 plane is 35.7°.

Notably, **7l**, **7h**, **11b**, **11f** and **11g** all have high binding ability to *Amine oxidase [flavin-containing] A* protein. In the flexible docking process, the conformation of the ligand compound is automatically adjusted according to the active site, while that of the crystal structure is different. A comparison of the two conformations showed that the indole ring and benzene ring hardly changed. Because C-C and C-N bonds can be twisted, the single chain region between indole ring and benzene ring is an important factor for conformational change, and is conducive to the reduction of steric hindrance of ligand binding to the receptor, as well as to the compound binding to the receptor. The binding of **7l** produces three hydrogen bonds, the binding of **7h** produces one, the binding of **11b** produces no hydrogen bonds, the binding of **11f** produces two hydrogen bonds, and the binding of **11g** produces one hydrogen bond. The greater the number of hydrogen bonds, the stronger the binding ability at the active site, and the more advantages compared with competitive binding ligands. The molecular energies of **7l**, **7h**, **11b**, **11f** and **11g** after docking with the receptor were −9.6 kcal/mol, −7.9 kcal/mol, −8.1 kcal/mol, −8.7 kcal/mol and −7.7 kcal/mol respectively. Among them, **7l** had the lowest molecular energy after binding with the receptor, indicating that its structure is more stable. 

## 3. Discussion

Target compounds **7l** and **7h** were obtained by connecting the cinnamic acid moiety to the Harmine N9 site, while compounds **11b**, **11f** and **11g** were obtained by linking the benzene ring-containing group to tetrahydro-β-Carboline. They were characterized by ^1^H NMR, ^13^C NMR and ESI-MS. The crystal structure of compound **7l** was determined. By simulating the binding process between ligand molecule and receptor, compounds **7l**, **7h**, **11f** and **11g** were shown to be able to form strong hydrogen bonds with receptor *Amine oxidase [flavin-containing] A*. Target compound **7l** connects to the receptor *Amine oxidase [flavin-containing] A* protein as a target molecule. Compared with harmine, target compound **7l** can bind to the receptor *Amine oxidase [flavin-containing] A* protein more effectively through hydrogen bonding. Cow mastitis is a disease caused by a variety of pathogenic bacteria. Compound **7l**, synthesized in the present research, has good inhibitory activity against its pathogenic bacteria. In vitro, the antibacterial activity of compound **7l** was better than that of Harmine. Compound **7l** had good bactericidal activity against Streptococcus lactis with a minimum inhibitory concentration (MIC) of 43.7 μg/mL. This study is of great significance for the treatment of dairy cow mastitis, and is of guiding significance for the transformation of Harmine and tetrahydro-β-Caroline. The antibacterial activity of the compound is worthy of further study.

## 4. Materials and Methods

### 4.1. Instruments and Reagents

Positive control Harmine and cinnamic acid were purchased from Beijing Solarbio Technology Co., Ltd. (Beijing, China). All chemical reagents were purchased from Energy Reagent Co., Ltd. (Beijing, China). Or Shanghai Huangdi Chemical Co., Ltd. (Shanghai, China). Unless otherwise stated, all commercial reagents were used immediately upon receipt. HRMS was obtained with an ESI-Brucker APEX Ⅱ49e mass spectrometer (Bruker, Billerica, MA, USA). The ^1^H NMR and ^13^C NMR spectra were recorded with a JNM-ECS (JEOL Co., Ltd., Tokyo, Japan) and Bruke AM-400 spectrometer (Bruker, Marietta, GA, USA), respectively. The solvents used were deuterated reagents (DMSO-d6 and CDCl_3_). Data are presented as follows: chemical shift (ppm), multiplicity (s = singlet, d = doublet, t = triplet, dd = doublet of doublets, m = multiplet, br = broad), coupling constant J (Hz) and integration. To monitor the reaction, thin layer chromatography (TLC) was performed on a silica gel plate (Qingdao Ocean Chemical Co., Ltd., Shandong, China) and observed with an ULTRAVIOLET lamp. The product is further purified by column chromatography on silica gel (200–300 mesh, Gansu Yihua Chemical Glass Instrument Co., Ltd., Lanzhou, China) and eluted by air pressure in an appropriate solvent mixture. Crystals of the target compounds were obtained by solvent evaporation at 25 °C and their crystal structures were determined using a Supernova single crystal diffractometer.

### 4.2. MIC Testing

In this study, the microplate dilution method was used to screen the antibacterial activity of **7l** in vitro [7]. The investigation included three Gram-positive strains, *Streptococcus dysgalactiae* ATCC 35666, *Bacillus subtilis* CMCC(B) 63501 and *Streptococcus pyogenes* ATCC 19615, and one Gram-negative strain, *Proteusbacillus vulgaris* CMCC(B) 49027. Harmine and cinnamic acid were used as control drugs.

### 4.3. Bactericidal Time-Kill Kinetics

*Streptococcus dysgalactiae* was prepared in Mueller Hinton broth by shaking at 37 °C for 6 h. Next, **7l** (1 × MIC, 2 × MIC, 4 × MIC), Harmine (1 × MIC, 2 × MIC, 4 × MIC) and normal saline (as the growth control group) were added into the bacterial suspension, and the final bacterial concentration was 10^6^–10^7^ CFU/mL. At a predetermined time point (0, 0.5, 1, 2, 4, 6, 12 and 24 h), viable bacteria were counted and cultured at 37 °C for 24 h [8,9]. Bacterial concentrations are expressed as Log_10_ (CFU/mL). The same procedure was repeated three times.

### 4.4. Synthesis and Crystallization

The 6-methoxtryptamine was dissolved in 40 mL HCl solution (0.1N) and stirred at room temperature until clarified. Next, 5 mL of 35% acetaldehyde solution (20 mmol) was slowly added to the reaction solution. The reaction was carried out at 40 °C for 6 h, the reaction liquid was cooled to room temperature and the pH was adjusted to 10. After extraction with dichloromethane, the organic phase was combined, washed with saturated NaCl solution, dried with anhydrous sodium sulfate, filtered, decompressed and suspended by steaming, and purified by column chromatography to prepare the compound for use [10].

Triethylamine was dropped into 3-(4-methoxyphenyl) acrylic acid (1 mmol) acetone and stirred into the above compound until it was completely dissolved. Then, 9-(4-bromobutyl-7-methoxy-1-methyl-9H-pyridine [3,4-b] indole (3 mmol) was dissolved in the reaction solution and stirred continuously at 25 °C for 3 h. The reaction process was monitored by TLC. After the mixture has completely reacted, a solution of hydrochloric acid add to adjust the pH to 3–5 [11,12,13]. The mixture was extracted three times with ethyl acetate. The organic phase was extracted and washed with saturated NaHCO_3_ solution and saturated NaCl solution, respectively. The organic phase was dried on anhydrous Na_2_SO_4_ and concentrated by filtration. Compound **7l** was further purified by silica gel column chromatography [14,15].

3-(7-methoxy-1-methyl-9H-pyridine [3,4-b] indole-9-base) propyl (E)-3-(4-methoxy phenyl) acrylate(**7a**) Yellow solid, 0.45g (yield 52.1%), m.p. 133.2–133.9 °C, ^1^H NMR (400 MHz, CDCl_3_) δ: 8.29 (d, *J* = 5.2 Hz, 1H), 7.96 (d, *J* = 8.4 Hz, 1H), 7.73 (d, *J* = 5.2 Hz, 1H), 7.65 (d, *J* = 16.0 Hz, 1H), 7.49 (d, *J* = 8.8 Hz, 2H), 6.94–6.87 (m, 4H), 6.29 (d, *J* = 16.0 Hz, 1H), 4.65 (t, *J* =7.6 Hz, 2H), 4.28 (t, *J* = 5.8 Hz, 2H), 3.91 (s, 3H), 3.86 (s, 3H), 3.06 (s, 3H), 2.28–2.22 (m, 2H); ^13^C NMR (100 MHz, CDCl_3_) δ: 166.97, 161.53, 160.89, 144.97, 142.95, 140.47, 138.48, 135.12, 129.76, 129.48, 122.35, 115.21, 114.79, 114.33, 112.22, 108.82, 93.21, 61.42, 55.58, 55.34, 41.79, 29.86, 23.35; HRMS (ES) calcd [M + H]^+^ for C_26_H_26_N_2_O_4_ 431.1970, found 431.1923.

3-(7-methoxy-1-methyl-9H-pyridine [3,4-b] indole-9-base) propyl (E)-3-(three, four, five-phenyl 3 armour oxygen radicals) acrylate (**7b**) White solid, 0.41g(yield 49.2%), m.p. 102.6–104.2 °C, ^1^H NMR (400 MHz, CDCl_3_) δ: 8.29 (d, *J* = 5.2 Hz, 1H), 7.96 (d, *J* = 9.2 Hz, 1H), 7.73 (d, *J* = 5.2 Hz, 1H), 7.58 (d, *J*= 16.0 Hz, 1H), 6.90 (s, 2H), 6.76 (s, 2H), 6.31 (d, *J* = 16.0 Hz, 1H), 4.64 (t, *J* = 7.4 Hz, 2H), 4.29 (t, *J* = 5.8 Hz, 2H), 3.92 (s, 9H), 3.90 (s, 3H), 3.05 (s, 3H), 2.28–2.22 (m, 2H); ^13^C NMR (100 MHz, CDCl_3_) δ: 166.75, 161.05, 153.56, 145.42, 143.12, 140.54, 140.44, 138.59, 135.28, 129.73, 122.54, 116.68, 115.41, 112.40, 108.90, 105.41, 93.48, 61.80, 61.08, 56.28, 55.77, 41.97, 31.82, 29.95, 29.36, 23.45; HRMS (ES) calcd [M + H]^+^ for C_28_H_30_N_2_O_6_ 491.2182, found 491.2131.

4-(7-methoxy-1-methyl-9H-pyridine [3,4-b] indole-9-base) propyl (E)-3-(2-nitro phenyl) acrylate (**7c**) Yellow solid, 0.38 g (yield 44.2%), m.p. 128.6–129.3 °C, ^1^H NMR (400 MHz, CDCl_3_) δ: 8.29 (d, *J* = 5.2 Hz, 1H), 8.14 (d, *J* = 16.0 Hz, 1H), 8.08 (d, *J* = 7.2 Hz, 1H), 7.97 (d, *J* = 9.2 Hz, 1H), 7.73–7.64 (m, 3H), 7.60–7.56 (m, 1H), 6.90–6.87 (m, 2H), 6.33 (d, *J* = 16.0 Hz, 1H), 4.67 (t, *J* = 7.4 Hz, 2H), 4.33–4.29 (m, 2H), 3.92 (s, 3H), 3.05 (s, 3H), 2.30–2.24 (m, 2H); ^13^C NMR (100 MHz, CDCl_3_) δ: 165.67, 160.75, 148.18, 142.94, 140.40, 139.88, 138.10, 135.16, 130.41, 130.19, 129.26, 128.98, 124.79, 123.03, 122.25, 115.09, 112.14, 108.53, 93.31, 64.62, 55.60, 44.66, 28.47, 23.31; HRMS (ES) calcd [M + H]^+^ for C_25_H_23_N_3_O_5_ 446.1716, found 446.1756.

4-(7-methoxy-1-methyl-9H-pyridine [3,4-b] indole-9-base) butyl (E)-3-(4-methoxy phenyl) acrylate(**7d**) White solid, 0.38g(yield 43.6%), m.p. 118.7–120.2 °C, ^1^H NMR (400 MHz, CDCl_3_) δ: 8.29 (d, *J* = 5.2 Hz, 1H), 7.98 (d, *J* = 9.6 Hz, 1H), 7.74 (d, *J* = 5.2 Hz, 1H), 7.63 (d, *J* = 16.0 Hz, 1H), 7.46 (d, *J* = 8.8 Hz, 2H), 6.91–6.88 (m, 4H), 6.29 (d, *J* = 16.0 Hz, 1H), 4.54 (t, *J* = 7.6 Hz, 2H), 4.25 (t, *J* = 6.4 Hz, 2H), 3.94 (s, 3H), 3.84 (s, 3H), 3.03(s, 3H), 2.01–1.94 (m, 2H), 1.867–1.80 (m, 2H); ^13^C NMR (100 MHz, CDCl_3_) δ: 167.21, 161.44, 160.88, 144.76, 142.95, 140.44, 138.38, 135.21, 129.75, 129.42, 126.95, 122.39, 115.23, 115.12, 114.30, 112.26, 108.66, 93.37, 63.47, 55.68, 55.67, 44.38, 27.18, 26.13, 23.41; HRMS (ES) calcd [M + H]^+^ for C_27_H_28_N_2_O_4_ 445.2127, found 445.1884.

7-methoxy-4- (1-methyl-9H-pyridine [3,4-b] indole-9-base) butyl (E)-3-(three, four, five, three oxygen radicals phenyl) acrylate(**7e**) Yellow solid, 0.42g (yield 49.0%), m.p. 95.7–96.3 °C, ^1^H NMR (400 MHz, CDCl_3_) δ: 8.29 (d, *J* = 5.2 Hz, 1H), 7.99 (d, *J* = 9.2 Hz, 1H), 7.75 (d, *J* = 5.2 Hz, 1H), 7.59 (d, *J* = 16.0 Hz, 1H), 6.91–6.89 (m, 2H), 6.74 (s, 2H), 6.31 (d, *J* = 16.0 Hz, 1H), 4.55 (t, *J*= 7.8 Hz, 2H), 4.26 (t, *J* = 6.2 Hz, 2H), 3.94 (s, 3H), 3.89 (s, 9H), 3.03 (s, 3H), 2.02–1.95 (m, 2H), 1.87–1.80 (m, 2H); ^13^C NMR (100 MHz, CDCl_3_) δ: 166.83, 160.87, 153.40, 145.08, 142.95, 140.41, 140.19, 138.40, 135.21, 129.69, 129.44, 122.42, 116.87, 115.26, 112.28, 108.61, 105.23, 93.43, 63.67, 60.93, 56.13, 55.69, 44.37, 27.19, 26.12, 23.42; HRMS (ES) calcd [M + H]^+^ for C_29_H_32_N_2_O_6_ 505.2338, found 505.2361.

4-(7-methoxy-1-methyl-9H-pyridine [3,4-b] indole-9-base) butyl (E)-3-(2-nitro phenyl) acrylate(**7f**) White solid, 0.37g(yield 43.6%), m.p. 130.9–132.2 °C, ^1^H NMR (400 MHz, CDCl_3_) δ: 8.28 (d, *J* = 5.2 Hz, 1H), 8.11 (d, *J* = 15.6 Hz, 1H), 8.04 (d, *J* = 8.0 Hz, 1H), 7.97 (d, *J* = 9.2 Hz, 1H), 7.73 (d, *J* = 5.2 Hz, 1H), 7.67–7.60 (m, 2H), 7.56 –7.52(m, 1H), 6.90–6.87 (m, 2H), 6.34 (d, *J* = 16.0 Hz, 1H), 4.54 (t, *J* =7.8 Hz, 2H), 4.27 (t, *J* = 6.4 Hz, 2H), 3.94 (s, 3H), 3.03 (s, 3H), 2.01–1.93 (m, 2H), 1.87–1.80 (m, 2H); ^13^C NMR (100 MHz, CDCl_3_) δ: 165.62, 160.88, 148.22, 142.94, 140.41, 140.38, 138.36, 135.17, 133.49, 130.40, 130.33, 129.43, 129.08, 124.88, 122.73, 122.37, 115.20, 112.24, 108.69, 93.34, 64.07, 55.67, 44.32, 27.16, 26.03, 23.38; HRMS (ES) calcd [M + H]^+^ for C_26_H_25_N_3_O_5_ 460.1872, found 460.1619.

7-methoxy-5-(1-methyl-9H-pyridine [3,4-b] indole-9-base) amyl (E)-3-(4-methoxy phenyl) acrylate(**7g**) White solid, 0.40g(yield 46.2%), m.p. 91.1–92.3 °C, ^1^H NMR (400 MHz, DMSO-*d*_6_) δ: 8.17 (d, *J* = 5.2 Hz, 1H), 8.08 (d, *J* = 8.4 Hz, 1H), 7.88 (d, *J* = 5.2 Hz, 1H), 7.66 (d, *J* = 8.8 Hz, 2H), 7.57 (d, *J* = 16.0 Hz, 1H), 7.20 (s, 1H), 6.98 (d, *J* = 8.4 Hz, 2H), 6.87 (d, *J* = 8.6 Hz, 1H), 6.44 (d, *J* = 16.0 Hz, 1H), 4.57 (t, *J* = 7.6 Hz, 2H), 4.13 (t, *J* = 6.6 Hz, 2H), 3.91 (s, 3H), 3.81 (s, 3H), 2.96 (s, 3H), 1.82–1.74 (m, 2H), 1.72–1.69 (m, 2H); 1.51–1.43 (m, 2H); ^13^C NMR (100 MHz, CDCl_3_) δ: 167.22, 161.33, 160.84, 144.44, 143.00, 140.31, 138.03, 135.15, 129.66, 129.40, 126.99, 122.33, 115.31, 115.10, 114.24, 112.21, 108.64, 93.32, 63.79, 55.63, 55.30, 44.66, 30.23, 28.50, 23.38, 23.20; HRMS (ES) calcd [M + H]^+^ for C_28_H_30_N_2_O_4_ 459.2283, found 459.2315.

7-methoxy-5-(-methyl-9H-pyridine [3,4-b] indole-9-base) amyl (E)-3- (three, four, five, three oxygen radicals phenyl) acrylate(**7h**) White solid, 0.40g(yield 48.9%), m.p. 103.7–104.7 °C, ^1^H NMR (300 MHz, DMSO-*d*_6_) δ: 8.16 (d, *J* = 5.1 Hz, 1H), 8.08 (d, *J* = 8.4 Hz, 1H), 7.87 (d, *J* = 5.4 Hz, 1H), 7.57 (d, *J* = 15.9 Hz, 1H), 7.20 (s, 1H), 7.07 (s, 2H), 6.86 (d, *J* = 8.7 Hz, 1H), 6.64 (d, *J* = 15.9 Hz, 1H), 4.56 (t, *J* = 7.4 Hz, 2H), 4.14 (t, *J* = 6.5 Hz, 2H), 3.91 (s, 3H), 3.83 (s, 6H), 3.70 (s, 3H), 2.95 (s, 3H), 1.81–1.69 (m, 4H), 1.53–1.44 (m, 2H); ^13^C NMR (100 MHz, DMSO-*d*_6_) δ: 166.88, 160.99, 153.55, 145.16, 143.21, 140.97, 139.93, 138.19, 135.02, 130.08, 128.90, 122.81, 117.76, 114.68, 112.67, 109.54, 106.40, 94.12, 64.19, 60.56, 56.50, 56.04, 44.41, 30.39, 28.47, 23.53, 23.20; HRMS (ES) calcd [M + H]^+^ for C_30_H_34_N_2_O_6_ 519.2495, found 519.2474.

7-methoxy-5-(1-methyl-9H-pyridine [3,4-b] indole-9-base) amyl (E)-3-(2-nitro phenyl) acrylate(**7i**) Yellow solid, 0.38g (yield 44.2%), m.p. 127.4–130.0 °C, ^1^H NMR (400 MHz, CDCl_3_) δ: 8.28 (d, *J* = 5.2 Hz, 1H), 8.11 (d, *J* = 16.0 Hz, 1H), 8.06 (d, *J* = 8.0 Hz, 1H), 7.97 (d, *J* = 9.2 Hz, 1H), 7.73 (d, *J* = 5.6 Hz, 1H), 7.69–7.62 (m, 2H), 7.54–7.58 (m, 1H), 6.89–6.86 (m, 2H), 6.34 (d, *J* = 16.0 Hz, 1H), 4.53–4.49 (m, 2H), 4.24 (t, *J* = 6.4 Hz, 2H), 3.95 (s, 3H), 3.03 (s, 3H), 1.95–1.87 (m, 2H), 1.82–1.75 (m, 2H), 1.59–1.50 (m, 2H); ^13^C NMR (100 MHz, CDCl_3_) δ: 165.82, 158.08, 148.23, 144.12, 140.01, 138.76, 136.94, 133.49, 130.58, 130.22, 129.14, 127.31, 124.87, 123.16, 118.24, 115.39, 113.62, 108.74, 104.15, 99.94, 64.73, 62.57, 59.60, 33.71, 32.46, 31.74, 24.40; HRMS (ES) calcd [M + H]^+^ for C_27_H_27_N_3_O_5_ 474.2056, found 474.2011.

6-(7-methoxy-1-methyl-9H-pyridine [3,4-b] indole-9-base) hexyl (E) -3-(4-methoxy phenyl) acrylate(**7j**) Yellow solid, 0.36g (yield 37.6%), m.p. 88.7–90.4 °C, ^1^H NMR (300 MHz, CDCl_3_) δ: 8.29 (d, *J* = 5.4 Hz, 1H), 7.97 (d, *J* = 8.4 Hz, 1H), 7.73 (d, *J* = 5.1Hz, 1H), 7.63 (d, *J* = 16.2 Hz, 1H), 7.46 (d, *J* = 8.5 Hz, 2H), 6.84–6.90(m, 4H), 6.29 (d, *J* = 15.9 Hz, 1H), 4.45 (t, *J* = 7.8 Hz, 2H), 4.18 (t, *J* =6.7 Hz, 2H), 3.94 (s, 3H), 3.82 (s, 3H), 3.02 (s, 3H), 1.90–1.81 (m, 2H), 1.74–1.66 (m,2H), 1.50–1.45 (m, 4H); ^13^C NMR (100 MHz, CDCl_3_) δ: 167.25, 161.27, 160.76, 144.30, 142.95, 140.37, 138.08, 135.16, 129.60, 129.28, 127.00, 122.28, 115.42, 115.11, 114.21, 112.15, 108.51, 93.31, 64.03, 55.60, 55.26, 44.68, 30.47, 28.60, 26.52, 25.76, 23.30; HRMS (ES) calcd [M + H]^+^ for C_29_H_32_N_2_O_4_ 473.2440, found 473.2393.

6-(7-methoxy-1-methyl-9H-pyridine [3,4-b] indole-9-base)hexyl (E)-3-(three, four, five, three oxygen radicals phenyl) acrylate(**7k**) Yellow solid, 0.36g (yield 38.1%), m.p. 73.7–73.9 °C, ^1^H NMR (400 MHz, CDCl_3_) δ: 8.29 (d, *J* = 5.2 Hz, 1H), 7.98 (d, *J* = 8.4 Hz, 1H), 7.74 (d, *J* = 5.2 Hz, 1H), 7.59 (d, *J* = 15.6 Hz, 1H), 6.91–6.86 (m, 2H), 6.75 (s, 2H), 6.33 (d, *J* = 16.0 Hz, 1H), 4.48 (t, *J* = 7.8 Hz, 2H), 4.20 (t, *J* = 6.6 Hz, 2H), 3.95 (s, 3H), 3.88 (s, 9H), 3.02 (s, 3H), 1.90–1.83 (m, 2H), 1.74–1.69 (m, 2H), 1.50–1.47 (m, 4H); ^13^C NMR (100 MHz, CDCl_3_) δ: 166.95, 160.86, 153.36, 144.70, 143.08, 140.30, 140.05, 137.94, 135.19, 129.81, 129.46, 122.40, 117.21, 115.15, 112.26, 108.57, 105.15, 93.44, 64.24, 60.91, 56.10, 55.68, 44.76, 30.50, 28.61, 26.55, 25.77, 23.19; HRMS (ES) calcd [M + H]^+^ for C_31_H_36_N_2_O_6_ 533.2651, found 533.2551.

6-(7-methoxy-1-methyl-9H-pyridine [3,4-b] indole-9-base) hexyl (E)-3-(2-nitro phenyl) acrylate(**7l**) White solid, 0.35g(yield 33.9%), m.p. 117.3–119.9 °C, ^1^H NMR (300 MHz, DMSO-*d*_6_) δ: 8.16 (d, *J* = 5.1 Hz, 1H), 8.08 (d, *J* = 8.7 Hz, 2H), 7.95–7.90 (m, 2H), 7.87 (d, *J* = 5.1 Hz, 1H), 7.78 (t, *J* = 7.5 Hz, 1H), 7.68 (t, *J* = 7.7 Hz, 1H), 7.18 (s, 1H), 6.86 (d, *J* = 8.7 Hz, 1H), 6.62 (d, *J* = 15.9 Hz, 1H), 4.55 (t, *J* = 7.4 Hz, 2H), 4.16 (t, *J* = 6.5 Hz, 2H), 3.90 (s, 3H), 2.95 (s, 3H), 1.78–1.71 (m, 2H), 1.66–1.60 (m, 2H), 1.45–1.40 (m, 4H); ^13^C NMR (100 MHz, CDCl_3_) δ: 165.67, 160.75, 148.18, 142.94, 140.40, 139.88, 138.10, 135.16, 133.40, 130.41, 130.19, 129.26, 128.98, 124.79, 123.03, 122.25, 115.09, 112.14, 108.53, 93.31, 64.62, 55.60, 44.66, 30.48, 28.47, 26.51, 25.76, 23.31; HRMS (ES) calcd [M + H]^+^ for C_28_H_29_N_3_O_5_ 488.2185, found 488.2201.

First, 1.8 mmol of the corresponding acid was weighed and dissolved in dry THF. Then, 2.16 mmol of SOCl_2_ was dropped into the system, refluxed at 65 °C for 1h, and the solvent was dried with a rotary evaporator set at a constant temperature to obtain acyl chloride for future use. Next, 1.5 mmol of intermediate (10) was dissolved in 10 mL dry tetrahydrofuran, and the acyl chloride obtained in the previous step was dissolved in 5 mL dry tetrahydrofuran before being slowly dropped into the reaction system, The reaction process is shown in Scheme 2. The reaction temperature was 25 °C, the reaction time was 5h, the solvent was evaporated by rotary evaporation, and saturated NaCl was washed with water three times. After drying with anhydrous sodium sulfate, the target compound was purified by chromatographic column (ethyl acetate: petroleum ether = 1:1).

(1-methyl-1,3,4,9-tetrahydro-2H-pyridyl [3,4-b] indole-2-) (2-chlorophenyl) ketone(**11a**) White solid, 0.41g(yield 49.3%), m.p. 224–225 °C, ^1^H NMR (400 MHz, DMSO-*d_6_*, TMS, ppm): δ 11.02 (s, 1H, NH), 7.58 (dd, *J* = 7.6, 1.5 Hz, 1H, Ar-H), 7.53–7.46 (m, 2H, Ar-H), 7.45 (dd, *J* = 6.9, 1.5 Hz, 1H, Ar-H), 7.37 (d, *J* = 7.7 Hz, 1H, Ar-H), 7.33 (d, *J* = 8.0 Hz, 1H, Ar-H), 7.10–7.04 (m, 1H, Ar-H), 6.97 (td, *J* = 7.5, 0.9 Hz, 1H, Ar-H), 5.71 (m, 1H, CH-N), 3.50–3.41 (m, 2H, CH_2_-N), 2.69–2.58 (m, 2H, CH_2_-CH_2_), 1.55 (d, *J* = 6.7 Hz, 3H, CH_3_). 13C NMR (100 MHz, DMSO-d6, TMS, ppm): δ 166.50, 136.62, 136.53, 135.38, 131.10, 130.13, 129.92, 128.32, 128.06, 126.74, 121.56, 119.13, 118.29, 111.64, 106.56, 61.48, 45.55, 21.98, 19.18. HRMS (ES) calcd [M + H]^+^ for C_19_H_17_N_2_O_1_Cl 325.1164, found 325.1112.

(1-methyl-1,3,4,9-tetrahydro-2H-pyridyl [3,4-b] indole-2-) (4-chlorophenyl) ketone(**11b**) Yellow solid, 0.55g (yield 69.9%), m.p. 213–214 °C, ^1^H NMR (400 MHz, DMSO-*d_6_*, TMS, ppm): δ 10.99 (s, 1H, NH), 7.55 (d, *J* = 8.1 Hz, 2H, Ar-H), 7.48 (d, *J* = 6.7 Hz, 2H, Ar-H), 7.40 (d, *J* = 7.6 Hz, 1H, Ar-H), 7.31 (t, *J* = 15.5 Hz, 1H, Ar-H), 7.06 (t, *J* = 6.7 Hz, 1H, Ar-H), 6.98 (t, *J* = 7.3 Hz, 1H, Ar-H), 5.71–5.50 (m, 1H, CH-N), 3.70 (m, CH_2_-N), 3.50–3.39 (m, 1H, CH_2_-N), 2.84–2.63 (m, 2H, CH_2_-CH_2_), 1.59–1.48 (d, *J* = 6.7 Hz, 3H, CH_3_).13C NMR (100 MHz, DMSO-d6, TMS, ppm):δ 168.02, 136.51, 135.93, 135.68, 134.70, 134.70, 129.22, 126.81, 126.81, 121.61, 119.12, 118.34, 111.69, 111.54, 106.52, 55.99, 46.00, 22.17, 19.35. HRMS (ES) calcd [M + H]^+^ for C_19_H_17_N_2_O_1_Cl 325.1164, found 325.1095.

(1-methyl-1,3,4,9-tetrahydro-2H-pyridyl [3,4-b] indole-2-) (2-methoxyphenyl) ketone(**11c**) White solid, 0.54g(yield 68.7%), m.p. 236–237 °C, ^1^H NMR (400 MHz, DMSO-*d_6_*, TMS, ppm): δ 10.87 (s, 1H, NH), 7.44–7.39 (m, 2H, Ar-H), 7.32 (d, *J* = 8.0 Hz, 1H, Ar-H), 7.19 (d, *J* = 6.2 Hz, 1H, Ar-H), 7.11 (d, *J* = 8.3 Hz,1H, Ar-H), 7.08–7.02 (m, 2H, Ar-H), 6.96 (t, *J* = 7.3 Hz, 1H, Ar-H), 5.78–5.60 (m, 1H, CH-N), 3.79 (s, *J* = 22.0 Hz, 3H, OCH_3_), 3.42 (m, *J* = 20.4, 10.9 Hz, 2H, CH_2_-N), 2.76–2.54 (m, 2H, CH_2_-CH_2_), 1.52 (d, *J* = 6.7 Hz, 3H, CH_3_).13C NMR (100 MHz, DMSO-*d*6, TMS, ppm):δ 167.53, 155.67, 136.55, 135.87, 135.81, 130.81, 128.26, 127.52, 126.80, 126.75, 121.45, 121.25, 119.05, 111.58, 106.77, 56.27, 55.78, 45.29, 22.29, 19.41. HRMS (ES) calcd [M + H]^+^ for C_20_H_20_N_2_O_2_ 321.1760, found 321.1606.

(1-methyl-1,3,4,9-tetrahydro-2H-pyridyl [3,4-b] indole-2-) (3-methoxyphenyl) ketone(**11d**) White solid, 0.55g(yield 68.7%), m.p. 150–151 °C, ^1^H NMR (400 MHz, DMSO-*d_6_*, TMS, ppm): δ 10.85 (s, 1H, NH), 7.42–7.36 (m, 2H, Ar-H), 7.30 (d, *J* = 7.4 Hz, 1H, Ar-H), 7.08–7.02 (m, 2H, Ar-H), 7.00–6.92 (m, 3H, Ar-H), 5.63 (m, 6.5 Hz, 1H, CH-N), 3.80 (s, 3H, OCH_3_), 3.34 (m, *J* = 12.6, 7.5 Hz, 2H, CH_2_-N), 2.83–2.62 (m, 2H, CH_2_-CH_2_), 1.53 (d, *J* = 6.6 Hz, 3H, CH_3_). 13C NMR (100 MHz, DMSO-*d*6, TMS, ppm):δ 169.62, 159.77, 138.59, 136.49, 135.79, 130.35, 126.83, 121.51, 119.10, 118.90, 118.28, 115.75, 112.20, 111.59, 106.58, 55.79, 45.83, 41.87, 22.29, 19.38. HRMS (ES) calcd [M + H]^+^ for C_20_H_20_N_2_O_2_ 321.1760, found 321.1605. ESI-MS: found 321.1605 for C_20_H_20_N_2_O_2_ [M + H] ^+^.

(1-methyl-1,3,4,9-tetrahydro-2H-pyridyl [3,4-b] indole-2-) (4-methoxyphenyl) one (**11e**) White solid, 0.55g(yield 70.8%), m.p. 232–233 °C, ^1^H NMR (400 MHz, DMSO-*d_6_*, TMS, ppm): δ 10.78 (s, 1H, NH), 7.39 (d, *J* = 8.6 Hz, 3H, Ar-H), 7.29 (d, *J* = 8.0 Hz, 1H, Ar-H), 7.08–6.94 (m, 4H, Ar-H), 5.57–5.30 (m, 1H, CH-N), 3.81 (s, 3H, OCH_3_), 3.42–3.21 (m, 2H, CH_2_-N), 2.72 (m, 2H, CH_2_-CH_2_), 1.53 (d, *J* = 6.7 Hz, 3H, CH_3_).13C NMR (100 MHz, DMSO-*d*6, TMS, ppm):δ 169.96, 160.68, 136.47, 135.96, 129.21, 129.00, 126.86, 126.86, 121.48, 119.08, 118.26, 114.32, 114.32, 111.58,106.70, 55.78, 42.05, 19.12. HRMS (ES) calcd [M + H]^+^ for C_20_H_20_N_2_O_2_ 321.1760, found 321.1610.

(1-methyl-1,3,4,9-tetrahydro-2H-pyridyl [3,4-b] indole-2 -) (2-nitrophenyl) ketone(**11f**) Yellow solid, 0.51g (yield 66.1%), m.p. 251–252 °C, ^1^H NMR (400 MHz, DMSO-*d_6_*, TMS, ppm): δ 11.03 (s, 1H, NH), 8.26 (d, *J* = 8.2 Hz, 1H, Ar-H), 7.89 (t, *J* = 7.3 Hz, 1H, Ar-H), 7.75 (t, *J* = 7.4 Hz, 1H, Ar-H), 7.61 (d, *J* = 7.2 Hz, 1H, Ar-H), 7.37 (dd, *J* = 17.5, 7.8 Hz, 2H, Ar-H), 7.06 (dd, *J* = 16.6, 8.7 Hz, 1H, Ar-H), 6.98 (t, *J* = 7.3 Hz, 1H, Ar-H), 5.59 (m, 1H, CH-N), 3.58–3.39 (m, 2H, CH_2_-N), 2.82–2.61 (m, 2H, CH_2_-CH_2_), 1.58 (d, *J* = 6.6 Hz, 3H, CH_3_). 13C NMR (100 MHz, DMSO-*d*6, TMS, ppm):δ 166.27, 145.69, 136.56, 135.37, 133.26, 130.85, 126.85, 126.77, 125.34, 121.56, 119.14, 118.39, 118.28, 111.65, 106.55, 50.86, 41.75, 21.84, 20.63. HRMS (ES) calcd [M + H]^+^ for C_19_H_17_N_3_O_3_ 336.1505, found 336.1354.

(1-methyl-1,3,4,9-tetrahydro-2H-pyridyl [3,4-b] indole-2-) (4-nitrophenyl) ketone(**11g**) Yellow solid, 0.48g (yield 59.7%), m.p. 268–269 °C, ^1^H NMR (400 MHz, DMSO-*d_6_*, TMS, ppm): δ 10.90 (s, 1H, NH), 8.31 (d, *J* = 8.7 Hz, 2H, Ar-H), 7.72 (d, *J* = 8.5 Hz, 2H, Ar-H), 7.40 (d, *J* = 7.8 Hz, 2H, Ar-H), 7.06 (t, *J* = 7.1 Hz, 1H, Ar-H), 6.98 (td, *J* = 7.5, 1.0 Hz, 1H, Ar-H), 5.80–5.57 (m, 1H, CH-N), 3.67–3.39 (m, 2H, CH_2_-N), 2.85–2.61 (m, 2H, CH_2_-CH_2_), 1.56 (d, *J* = 5.8 Hz, 3H, CH_3_). 13C NMR (100 MHz, DMSO-*d*6, TMS, ppm):δ 168.10, 148.34, 143.33, 136.52, 135.42, 128.48, 126.77, 126.77, 124.45, 124.45, 121.57, 119.15, 118.28, 111.64, 106.47, 46.00, 41.95, 22.15, 19.39. HRMS (ES) calcd [M + H]^+^ for C_19_H_17_N_3_O_3_ 336.1505, found 336.1361.

(1-methyl-1,3,4,9-tetrahydro-2H-pyridyl [3,4-b] indole-2-) (3,5-dinitrophenyl) ketone (**11h**) Red solid, 0.47g (yield 54.3%), m.p. 249 °C, ^1^H NMR (400 MHz, DMSO-*d_6_*, TMS, ppm): δ 11.02 (s, 1H), 8.90 (s, 1H, NH), 8.69 (d, *J* = 1.6 Hz, 2H, Ar-H), 7.42 (d, *J* = 7.6 Hz, 1H, Ar-H), 7.34 (d, *J* = 8.0 Hz, 1H, Ar-H), 7.07 (t, *J* = 7.4 Hz, 1H, Ar-H), 6.98 (t, *J* = 7.4 Hz, 1H, Ar-H), 5.67 (dd, *J* = 12.8, 6.6 Hz, 1H, CH-N), 3.72 (dd, *J* = 13.8, 4.4 Hz, 1H, CH_2_-N), 3.58–3.47 (m, 1H, CH_2_-N), 2.81 (dd, *J* = 19.0, 8.1 Hz, 1H, CH_2_-CH_2_), 2.66 (d, *J* = 12.7 Hz, 1H, CH_2_-CH_2_), 1.60 (d, *J* = 6.6 Hz, 3H, CH_3_). 13C NMR (100 MHz, DMSO-*d*6, TMS, ppm):δ 165.79, 148.77,148.77, 139.80, 136.51, 135.34, 127.81, 126.76, 121.57, 119.69, 119.14, 118.32, 111.61, 106.49,100.00, 46.40, 42.19, 22.20, 19.36. HRMS (ES) calcd [M + H]^+^ for C_19_H_16_N_4_O_5_ 381.1356, found 381.2984.

(1-methyl-1,3,4,9-tetrahydro-2H-pyridinetrium [3,4-b] indole-2-) (2-nitro-5-methylphenyl) ketone (**11i**) Yellow solid, 0.45g (yield 53.8%), m.p. 258–260 °C, ^1^H NMR (400 MHz, DMSO-*d_6_*, TMS, ppm): δ 10.91 (s, 1H, NH), 8.14 (d, *J* = 8.4 Hz, 1H, Ar-H), 7.55–7.50 (m, 1H, Ar-H), 7.38 (d, *J* = 6.2 Hz, 2H, Ar-H), 7.34 (d, *J* = 8.0 Hz, 1H, Ar-H), 7.10–7.02 (m, 1H, Ar-H), 7.00–6.94 (m, 1H, Ar-H), 5.64 (m, 1H, CH-N), 3.48 (dd, *J* = 14.9, 9.2 Hz, 2H, CH_2_-N), 2.84–2.59 (m, 2H, CH_2_-CH_2_), 2.47 (s, 3H, Ar-CH_3_), 1.58 (d, *J* = 6.7 Hz, 3H, CH_3_). 13C NMR (100 MHz, DMSO-*d*6, TMS, ppm):δ 166.39, 147.05, 143.33, 136.55, 135.40, 133.41, 131.11, 126.86, 126.78, 125.31, 121.54, 119.12, 118.27, 111.64, 106.57, 65.55, 45.94, 21.42, 19.21, 18.30. HRMS (ES) calcd [M + H]^+^ for C_20_H_19_N_3_O_3_ 350.1661, found 350.1510.

First, 20 mg of the compound was added to 3 mL dichloromethane for ultrasonic treatment until it was completely dissolved. Next, 1mL of dichloromethane and n-hexane mixed buffer solution (1:1) was slowly added to the wall, and 9 mL n-hexane was added to the upper layer of the buffer solution [16,17]. Through slow evaporation of the solvent, crystals of compound **7l**, **7h**, **11b**, **11f** and **11g** were obtained [18].

Colorless crystals with appropriate size were selected for X-ray diffraction analysis. The temperature was maintained at 296K during data acquisition. Anisotropic thermal parameters were used to refine all non-hydrogen atoms. The hydrogen atoms were placed in the calculated positions. The crystal structure was analyzed by the direct solution method of SHELX.97 and refined by SHELX.97 [19,20]. Data collection: Bruker SMART APEX II; cell refinement: Bruker SMART; data reduction: Bruker SAINT; molecular graphics: SHELXTL.

### 4.5. Molecular Docking

According to the information registered in the DRUGBANK database, Harmine has binding activity to *Amine oxidase [flavin-containing] A* protein. In this experiment, target compounds **7l**, **7h**, **11b**, **11f** and **11g** were selected to match and simulate molecular docking with one target of *Amine oxidase [flavin-containing] A* protein, and the corresponding crystal structure (PDBID:2Z5Y) of the protein was found from the RCSB PDB protein structure database as *Amine oxidase [flavin-containing] A*. Target compound **7l**, **7h**, **11b**, **11f** and **11g** actedas the ligand. The PyMOL and AutoDock Tools were used to process 2Z5Y protein receptor and **7l**, **7h**, **11b**, **11f** and **11g** ligand, and to find the active pocket position. AutoDock Vina software was used to dock the ligand with the receptor to obtain the binding energy.

The X-ray crystallographic coordinates for the structures reported in this study have been deposited at the Cambridge Crystallographic Data Centre (CCDC) under deposition numbers 2118489(**7l**), 2155794(**7h**), 2155796(**11b**), 2155798(**11f**) and 2155799(**11g**). The data can be obtained free of charge from The Cambridge Crystallographic Data Centre via https://www.ccdc.cam.ac.uk/data_request/cifwww.ccdc.cam.ac.uk/data_request/cif(accessed on 20 April 2022).

## Figures and Tables

**Table 1:** Minimum Inhibitory Concentration (MICs) (μg·mL^−1^).

Compound	*S. dysgalactiae*	*P. vulgaris*	*B. subtilis*	*S. pyogenes*
**7a**	87.5	175	350.0	175.0
**7b**	175.0	350	700.0	175.0
**7c**	87.5	175	350.0	350.0
**7d**	175.0	175	700.0	175.0
**7e**	87.5	350	1400.0	175.0
**7f**	87.5	350	700.0	175.0
**7g**	175.0	175	700.0	350.0
**7h**	87.5	175.0	350.0	175.0
**7i**	87.5	350	350.0	175.0
**7j**	175.0	350	700.0	175.0
**7k**	175.0	350	350.0	175.0
**7l**	43.7	175.0	175.0	87.5
**11a**	175.0	350	700.0	175.0
**11b**	87.5	175.0	350.0	350.0
**11c**	350.0	350	350.0	350.0
**11d**	350.0	350	500.0	175.0
**11e**	350.0	350	1400.0	350.0
**11f**	175.0	700.0	700.0	350.0
**11g**	175.0	350.0	700.0	175.0
**11h**	350.0	350	500.0	350.0
**11i**	175.0	350	700.0	175.0
Harmine	175.0	350.0	350.0	175.0
cinnamic acid	500.0	500.0	500.0	500.0

**Table 2:** Crystallographic data and structure refinement parameters of compound **7l**.

Empirical Formula	C_28_H_29_N_3_O_5_
Temperature (K)	296
Crystal system	Monoclinic
Space group	P21/c
a/ Å	8.7050 (11)
b/Å	27.138 (3)
c/ Å	10.4121 (14)
α/°	90
β/°	92.221 (2)
γ/°	90
Volume/Å^3^	2457.9 (5)
Z	4
Ρ_calc_ mg/mm^3^	1.318
μ(Mo Kα) (mm^−1^)	0.09
F (0 0 0)	1032
Crystal size (mm^3^)	0.30 × 0.20 × 0.20
Radiation	Mo Kα (λ = 0.71073)
CCDC deposit number	2,118,489

## Data Availability

The original contributions presented in the study are included in the article and Supplementary Material, further inquiries can be directed to the corresponding author.

## Supplementary Materials

The following are available online at https://www.mdpi.com/article/10.3390/molecules27092888/s1, Figures S1–S63: The ^1^H-NMR, ^13^C-NMR and ESI-MS spectrum of compound **7a-l** and **11a-i**. Tables S1–S6: Crystal data of compound **7l**; Tables S7–S12: Crystal data of compound **7h**; Tables S13–S18: Crystal data of compound **11b**; Tables S19–S24: Crystal data of compound **11f**; Tables S25–S30: Crystal data of compound **11g**.

## Sample Availability

All samples of the compound are available from the author.

## References

[1] Ansari M.N. (2020). Assessment of Antidiarrheal, Antispasmodic and Antimicrobial Activities of Methanolic Seeds Extract of *Peganum harmala* L. (Nitrariaceae). Pharm. Res. Int..

[2] Moustafa N.E., Alomari A.A. (2019). Green synthesis and bactericidal activities of isotropic and anisotropic spherical gold nanoparticles produced using *Peganum harmala* L. leaf and seed extracts. Biotechnol. Appl. Biochem..

[3] Zhang L., Li D., Yu S. (2020). Pharmacological effects of harmine and its derivatives: A review. Arch. Pharmacal Res..

[4] Amsaraj C., Bharathikannan R., Muthuraja P., Rajkumar M. (2021). Analyzing integrated π…π, C[sbnd]H…π and hydrogen bonding interactions in N, N-Dimethyl-4-aminopyridinium benzilate. Mol. Struct..

[5] Huseynzada A.E., Jelsch C., Akhundzada H.N., Soudani S., Nasr C.B., Doria F., Hasanova U.A., Freccero M. (2020). Synthesis, crystal structure and antibacterial properties of 6-methyl-2-oxo-4-(quinolin-2-yl)-1,2,3,4-tetrahydropyrimidine-5-carboxylate. Mol. Struct..

[6] Son S.-Y., Ma J., Kondou Y., Yoshimura M., Yamashita E., Tsukihara T. (2008). Structure of human monoamine oxidase A at 2.2-A resolution: The control of opening the entry for substrates/inhibitors. Proc. Natl. Acad. Sci. USA.

[7] Shaheen H.A., Issa M.Y. (2020). In vitro and in vivo activity of *Peganum harmala* L. alkaloids against phytopathogenic bacteria. Sci. Hortic..

[8] Iranshahy M., Bazzaz S.F., Haririzadeh G., Abootorabi B.Z., Mohamadi A.M., Khashyarmanesh Z. (2019). Chemical composition and antibacterial properties of *Peganum harmala* L. Avicenna J. Phytomedicine.

[9] Liu W., Liu X., Tian L., Gao Y., Liu W., Chen H., Jiang X., Xu Z., Ding H., Zhao Q. (2021). Design, synthesis and biological evaluation of harmine derivatives as potent GSK-3β/DYRK1A dual inhibitors for the treatment of Alzheimer’s disease. Eur. J. Med. Chem..

[10] Dian H.E., Yang Z.Q., Hou M. (2014). Synthesis, crystal structure and antitumor activities of N-(2-(1*H*-indol-3-yl)ethyl)-2-nitroaniline. Chin. J. Struct. Chem..

[11] Chen L.-Y., Yang C.-Z., Xu Y., Qi C.-Y., Zhong Y., Wu B. (2021). Synthesis, Crystal Structure, and Biological Evaluation of (E )-1-(4-(4-Bromobenzyl)piperazin-1-YL)-3-(4-Chlorophenyl)PROP-2-en-1-one. Struct. Chem..

[12] Akabli T., Toufik H., Lamchouri F. (2020). In silico modeling studies of N9 -substituted harmine derivatives as potential anticancer agents: Combination of ligand-based and structure-based approaches. J. Biomol. Struct. Dyn..

[13] Sireesha R., Sreenivasulu R., Chandrasekhar C., Jadav S.S., Pavani Y., Rao M.V.B., Subbarao M. (2021). Design, synthesis, anti-cancer evaluation and binding mode studies of benzimidazole/benzoxazole linked β-carboline derivatives. Mol. Struct..

[14] Feng X., Fan S., Lv G., Yan M., Wu G., Jin Y., Yang Z. (2021). Expression, purification and X-ray crystal diffraction analysis of alcohol dehydrogenase 1 from *Artemisia annua* L. Protein Expr. Purif..

[15] Wassim M., Philippe G., Zakaria E. (2021). A new square pyramidal copper(II) complex [Cu(C10H24N4)Br]Br: Crystal structure, thermal analysis, Hirschfeld surfaces, electrical and semiconducting properties. Mol. Struct..

[16] Amanzhan A., Zhanymkhanova P.Z., Bagryanskaya I.Y., Shults E.E., Turmukhambetov A.Z., Adekenov S.M. (2021). Structure and Stereochemistry of a Hydrazone Derivative of Harmine. Struct. Chem..

[17] Magro F., Ceretti M., Meven M., Paulus W. (2021). Infrared furnace for in situ neutron single-crystal diffraction studies in controlled gas atmospheres at high temperatures. Appl. Crystallogr..

[18] Liang Y., He D., Zhou D., Li J., Tang L., Wang Z. (2021). Synthesis, Antibacterial and Pharmacokinetic Evaluation of Novel Derivatives of Harmine N9-Cinnamic Acid. Molecules.

[19] Sheldrick George M. (2015). Crystal structure refinement with SHELXL. Acta Crystallogr. Sect. C Struct. Chem..

[20] Sheldrick George M. (2015). SHELXT—Integrated space-group and crystal-structure determination. Acta Crystallogr. Sect. A Found. Adv..

