# Supplementary material for: Synthesis and Antibacterial Study of Novel Harmine Derivatives and Tetrahydro-β-Carboline Derivatives In Vitro
Source: Molecules. 2022 Apr 30;27(9):2888. doi: 10.3390/molecules27092888 (PMC9104299; doi:10.3390/molecules27092888)

checkCIF/PLATON report

Structure factors have been supplied for datablock(s) 1_a

THIS REPORT IS FOR GUIDANCE ONLY. IF USED AS PART OF A REVIEW PROCEDURE FOR PUBLICATION, IT SHOULD NOT REPLACE THE EXPERTISE OF AN EXPERIENCED CRYSTALLOGRAPHIC REFEREE.

No syntax errors found. CIF dictionary Interpreting this report

Datablock: 1_a

Bond precision:	C-C = 0.0023 A		Wavelength=0.71073
Cell:	a=8.3100(14)	b=14.485(3)	c=14.462(2)
	alpha=90	beta=105.111(3)	gamma=90
Temperature:	296 K		

	Calculated	Reported
Volume	1680.6(5)	1680.6(5)
Space group	P 21/n	P 21/n
Hall group	-P 2yn	-P 2yn
Moiety formula	C19 H17 N3 O3	?
Sum formula	C19 H17 N3 O3	C19 H19.50 N3 O3
Mr	335.36	337.87
Dx,g cm-3	1.325	1.335
Z	4	4
Mu (mm-1)	0.092	0.092
F000	704.0	714.0
F000'	704.32	
h,k,lmax	11,20,20	11,20,19
Nref	5124	4748
Tmin,Tmax	0.978,0.982	0.664,0.746
Tmin'	0.973	

Correction method= # Reported T Limits: Tmin=0.664 Tmax=0.746
AbsCorr = MULTI-SCAN

Data completeness= 0.927 Theta(max)= 30.510

R(reflections)= 0.0501(3159) wR2(reflections)= 0.1309(4748)

S = 0.999 Npar= 227

The following ALERTS were generated. Each ALERT has the format
test-name_ALERT_alert-type_alert-level.
Click on the hyperlinks for more details of the test.

Alert level C

```
PLAT041_ALERT_1_C Calc. and Reported SumFormula    Strings  Differ     Please Check
PLAT043_ALERT_1_C Calculated and Reported Mol. Weight Differ by ..       2.51 Check
PLAT068_ALERT_1_C Reported F000 Differs from Calcd (or Missing)...     Please Check
PLAT911_ALERT_3_C Missing FCF Refl Between Thmin & STh/L=    0.600          4 Report
PLAT934_ALERT_3_C Number of (Iobs-Icalc)/Sigma(W) > 10 Outliers ..          1 Check
```

Alert level G

```
FORMU01_ALERT_2_G  There is a discrepancy between the atom counts in the
            _chemical_formula_sum and the formula from the _atom_site* data.
            Atom count from _chemical_formula_sum:C19 H19.5 N3 O3
            Atom count from the _atom_site data:  C19 H17 N3 O3
CELLZ01_ALERT_1_G Difference between formula and atom_site contents detected.
CELLZ01_ALERT_1_G WARNING: H atoms missing from atom site list. Is this intentional?
           From the CIF: _cell_formula_units_Z    4
           From the CIF: _chemical_formula_sum  C19 H19.50 N3 O3
           TEST: Compare cell contents of formula and atom_site data

           atom    Z*formula  cif sites diff
           C         76.00     76.00    0.00
           H         78.00     68.00   10.00
           N         12.00     12.00    0.00
           O         12.00     12.00    0.00
PLAT007_ALERT_5_G Number of Unrefined Donor-H Atoms .............          1 Report
PLAT793_ALERT_4_G Model has Chirality at C11         (Centro SPGR)          R Verify
PLAT912_ALERT_4_G Missing # of FCF Reflections Above STh/L=  0.600        352 Note
PLAT941_ALERT_3_G Average HKL Measurement Multiplicity ..........        2.4 Low
PLAT965_ALERT_2_G The SHELXL WEIGHT Optimisation has not Converged     Please Check
PLAT978_ALERT_2_G Number C-C Bonds with Positive Residual Density.          7 Info
```

0 **ALERT level A** = Most likely a serious problem - resolve or explain
0 **ALERT level B** = A potentially serious problem, consider carefully
5 **ALERT level C** = Check. Ensure it is not caused by an omission or oversight
9 **ALERT level G** = General information/check it is not something unexpected

5 ALERT type 1 CIF construction/syntax error, inconsistent or missing data
3 ALERT type 2 Indicator that the structure model may be wrong or deficient
3 ALERT type 3 Indicator that the structure quality may be low
2 ALERT type 4 Improvement, methodology, query or suggestion
1 ALERT type 5 Informative message, check

It is advisable to attempt to resolve as many as possible of the alerts in all categories. Often the minor alerts point to easily fixed oversights, errors and omissions in your CIF or refinement strategy, so attention to these fine details can be worthwhile. In order to resolve some of the more serious problems it may be necessary to carry out additional measurements or structure refinements. However, the purpose of your study may justify the reported deviations and the more serious of these should normally be commented upon in the discussion or experimental section of a paper or in the "special_details" fields of the CIF. checkCIF was carefully designed to identify outliers and unusual parameters, but every test has its limitations and alerts that are not important in a particular case may appear. Conversely, the absence of alerts does not guarantee there are no aspects of the results needing attention. It is up to the individual to critically assess their own results and, if necessary, seek expert advice.

Publication of your CIF in IUCr journals

A basic structural check has been run on your CIF. These basic checks will be run on all CIFs submitted for publication in IUCr journals (*Acta Crystallographica*, *Journal of Applied Crystallography*, *Journal of Synchrotron Radiation*); however, if you intend to submit to *Acta Crystallographica Section C* or *E* or *IUCrData*, you should make sure that full publication checks are run on the final version of your CIF prior to submission.

Publication of your CIF in other journals

Please refer to the *Notes for Authors* of the relevant journal for any special instructions relating to CIF submission.

PLATON version of 19/02/2022; check.def file version of 19/02/2022

Datablock 1_a - ellipsoid plot

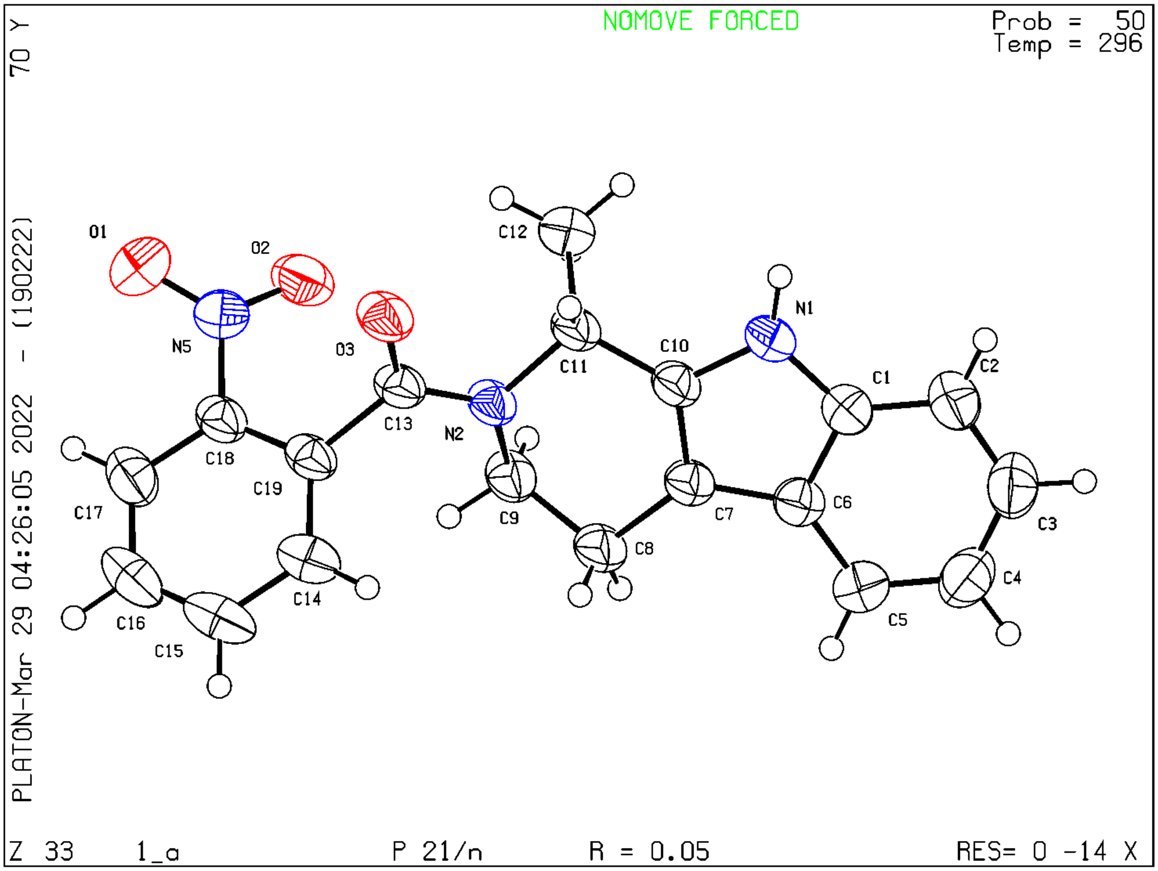

Supplement: Supplementary file 1 [file molecules-27-02888-s001.zip › checkcif (11f).pdf]
