# Supplementary material for: Synthesis and Antibacterial Study of Novel Harmine Derivatives and Tetrahydro-β-Carboline Derivatives In Vitro
Source: Molecules. 2022 Apr 30;27(9):2888. doi: 10.3390/molecules27092888 (PMC9104299; doi:10.3390/molecules27092888)

checkCIF/PLATON report

Structure factors have been supplied for datablock(s) 1_a

THIS REPORT IS FOR GUIDANCE ONLY. IF USED AS PART OF A REVIEW PROCEDURE FOR PUBLICATION, IT SHOULD NOT REPLACE THE EXPERTISE OF AN EXPERIENCED CRYSTALLOGRAPHIC REFEREE.

No syntax errors found. CIF dictionary Interpreting this report

Datablock: 1_a

Bond precision: C-C = 0.0030 A Wavelength=0.71073

Cell:	a=7.9502(12)	b=12.4483(19)	c=14.631(2)
	alpha=81.041(3)	beta=80.934(3)	gamma=73.883(3)
Temperature:	296 K		

	Calculated	Reported
Volume	1364.1(3)	1364.1(4)
Space group	P -1	P -1
Hall group	-P 1	-P 1
Moiety formula	C30 H34 N2 O6	?
Sum formula	C30 H34 N2 O6	C30 H34 N2 O6
Mr	518.59	518.59
Dx,g cm-3	1.263	1.263
Z	2	2
Mu (mm-1)	0.088	0.088
F000	552.0	552.0
F000'	552.27	
h,k,lmax	11,18,21	11,18,19
Nref	9000	7171
Tmin,Tmax	0.979,0.983	0.615,0.746
Tmin'	0.974	

Correction method= # Reported T Limits: Tmin=0.615 Tmax=0.746
AbsCorr = MULTI-SCAN

Data completeness= 0.797 Theta(max)= 31.405

R(reflections)= 0.0546(3498) wR2(reflections)= 0.1606(7171)

S = 0.999 Npar= 349

The following ALERTS were generated. Each ALERT has the format
test-name_ALERT_alert-type_alert-level.
Click on the hyperlinks for more details of the test.

Alert level C

```
PLAT026_ALERT_3_C Ratio Observed / Unique Reflections (too) Low ..        49% Check
PLAT230_ALERT_2_C Hirshfeld Test Diff for    O6       --C29      .        5.5 s.u.
PLAT230_ALERT_2_C Hirshfeld Test Diff for    C25      --C26      .        5.5 s.u.
PLAT911_ALERT_3_C Missing FCF Refl Between Thmin & STh/L=    0.600         34 Report
PLAT934_ALERT_3_C Number of (Iobs-Icalc)/Sigma(W) > 10 Outliers ..          1 Check
```

Alert level G

```
PLAT154_ALERT_1_G The s.u.'s on the Cell Angles are Equal ..(Note)      0.003 Degree
PLAT912_ALERT_4_G Missing # of FCF Reflections Above STh/L=  0.600       1689 Note
PLAT941_ALERT_3_G Average HKL Measurement Multiplicity ..........         1.3 Low
PLAT952_ALERT_5_G Calculated (ThMax) and CIF-Reported Lmax Differ.          2 Units
PLAT958_ALERT_1_G Calculated (ThMax) and Actual (FCF) Lmax Differ.          2 Units
PLAT965_ALERT_2_G The SHELXL WEIGHT Optimisation has not Converged     Please Check
PLAT978_ALERT_2_G Number C-C Bonds with Positive Residual Density.          6 Info
PLAT992_ALERT_5_G Repd & Actual _reflns_number_gt Values Differ by         10 Check
```

0 **ALERT level A** = Most likely a serious problem - resolve or explain
0 **ALERT level B** = A potentially serious problem, consider carefully
5 **ALERT level C** = Check. Ensure it is not caused by an omission or oversight
8 **ALERT level G** = General information/check it is not something unexpected

2 ALERT type 1 CIF construction/syntax error, inconsistent or missing data
4 ALERT type 2 Indicator that the structure model may be wrong or deficient
4 ALERT type 3 Indicator that the structure quality may be low
1 ALERT type 4 Improvement, methodology, query or suggestion
2 ALERT type 5 Informative message, check

It is advisable to attempt to resolve as many as possible of the alerts in all categories. Often the minor alerts point to easily fixed oversights, errors and omissions in your CIF or refinement strategy, so attention to these fine details can be worthwhile. In order to resolve some of the more serious problems it may be necessary to carry out additional measurements or structure refinements. However, the purpose of your study may justify the reported deviations and the more serious of these should normally be commented upon in the discussion or experimental section of a paper or in the "special_details" fields of the CIF. checkCIF was carefully designed to identify outliers and unusual parameters, but every test has its limitations and alerts that are not important in a particular case may appear. Conversely, the absence of alerts does not guarantee there are no aspects of the results needing attention. It is up to the individual to critically assess their own results and, if necessary, seek expert advice.

Publication of your CIF in IUCr journals

A basic structural check has been run on your CIF. These basic checks will be run on all CIFs submitted for publication in IUCr journals (*Acta Crystallographica*, *Journal of Applied Crystallography*, *Journal of Synchrotron Radiation*); however, if you intend to submit to *Acta Crystallographica Section C* or *E* or *IUCrData*, you should make sure that full publication checks are run on the final version of your CIF prior to submission.

Publication of your CIF in other journals

Please refer to the *Notes for Authors* of the relevant journal for any special instructions relating to CIF submission.

PLATON version of 19/02/2022; check.def file version of 19/02/2022

Datablock 1_a - ellipsoid plot

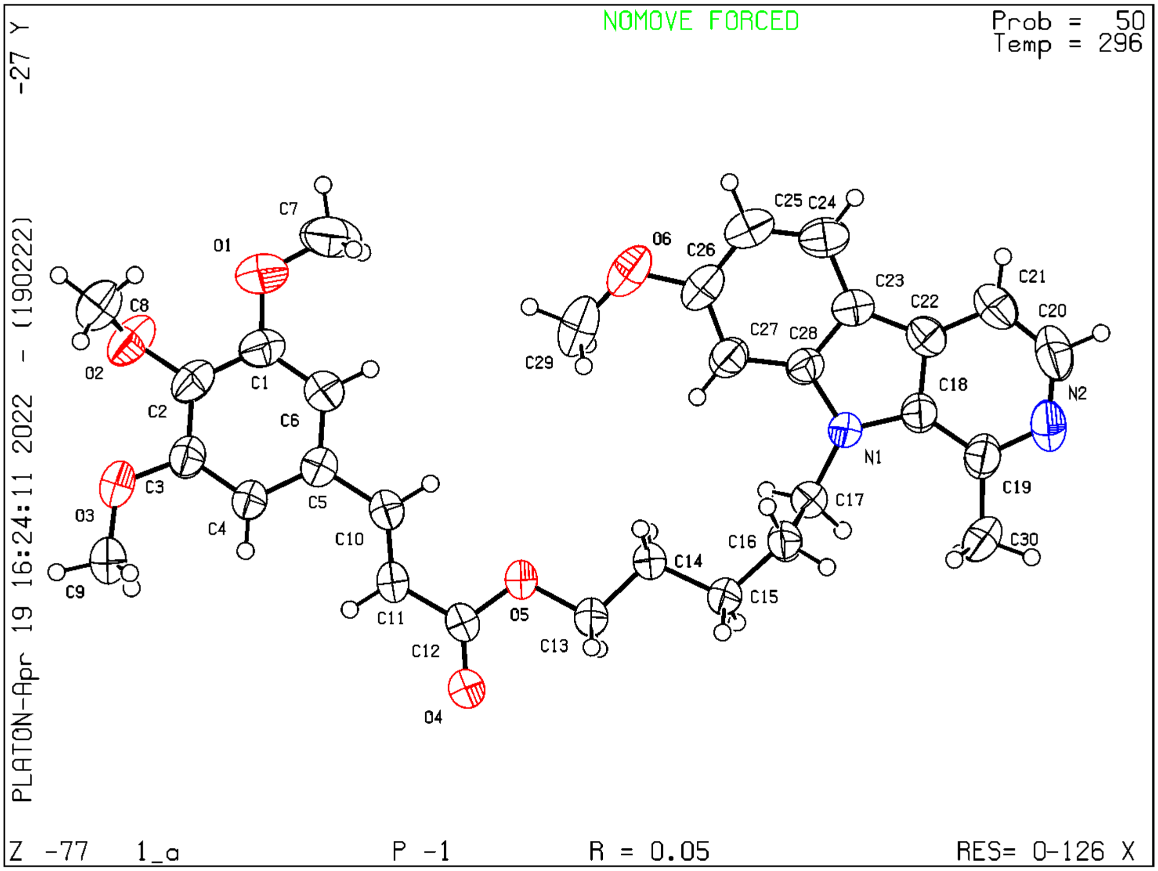

Supplement: Supplementary file 1 [file molecules-27-02888-s001.zip › checkcif (7h).pdf]
