# Supplementary material for: Synthesis and Antibacterial Study of Novel Harmine Derivatives and Tetrahydro-β-Carboline Derivatives In Vitro
Source: Molecules. 2022 Apr 30;27(9):2888. doi: 10.3390/molecules27092888 (PMC9104299; doi:10.3390/molecules27092888)
Supplement: Supplementary file 1 [file molecules-27-02888-s001.zip › supplementary materia.pdf]

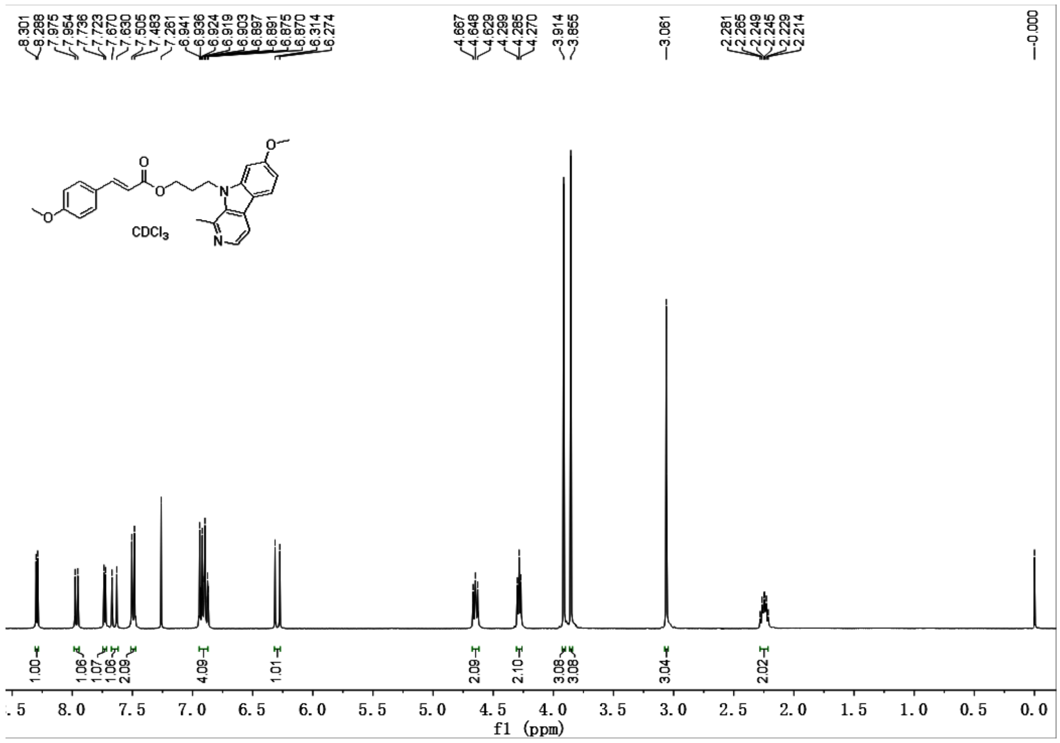

S1: 1H-NMR of 7a

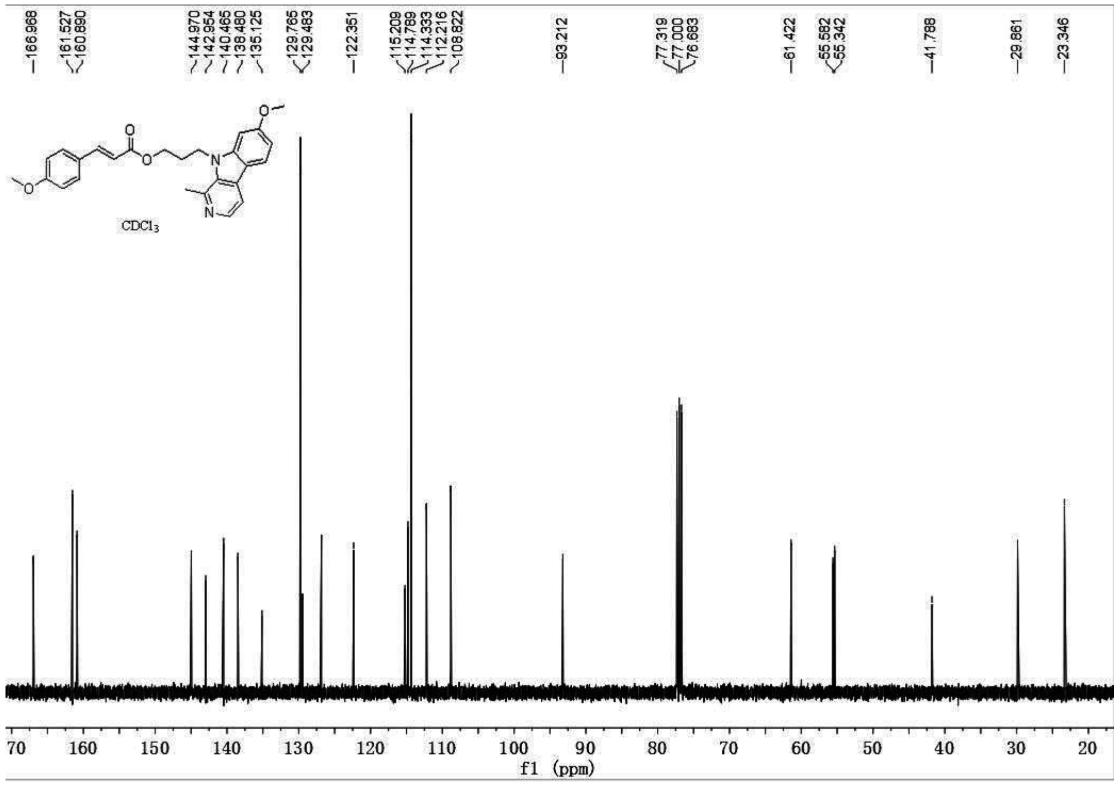

S2: 13C-NMR of 7a

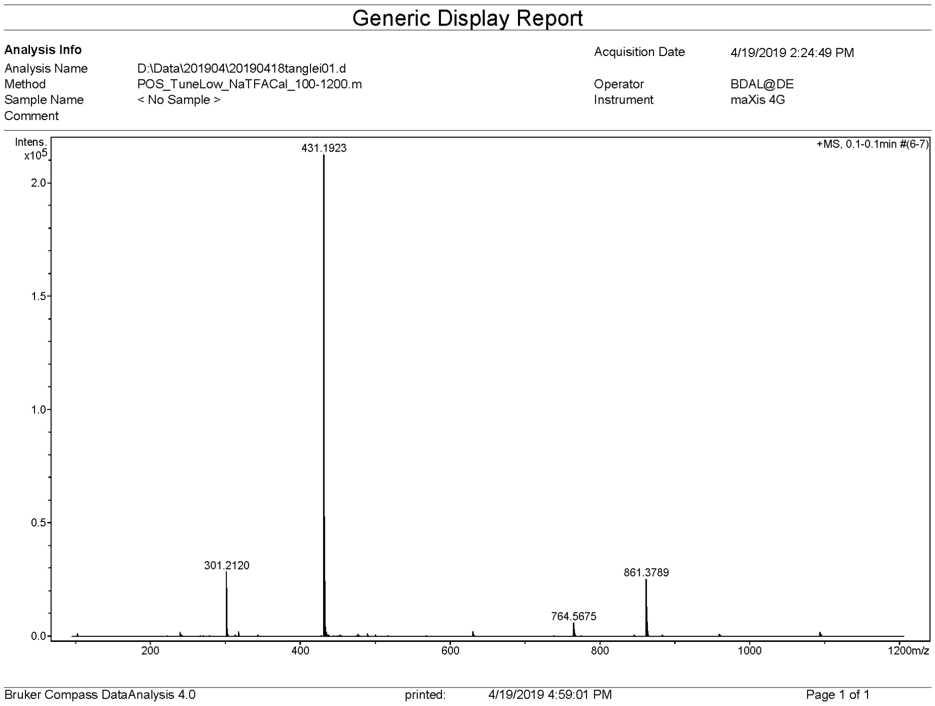

S3: ESI-MS of 7a

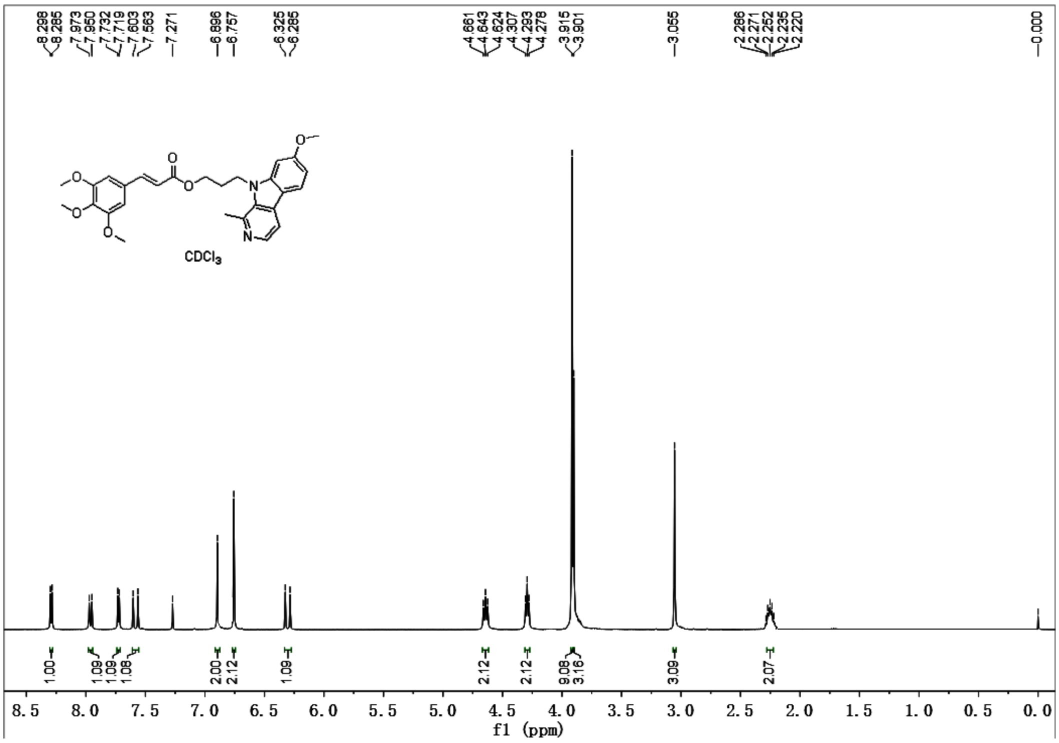

S4: 1H-NMR of 7b

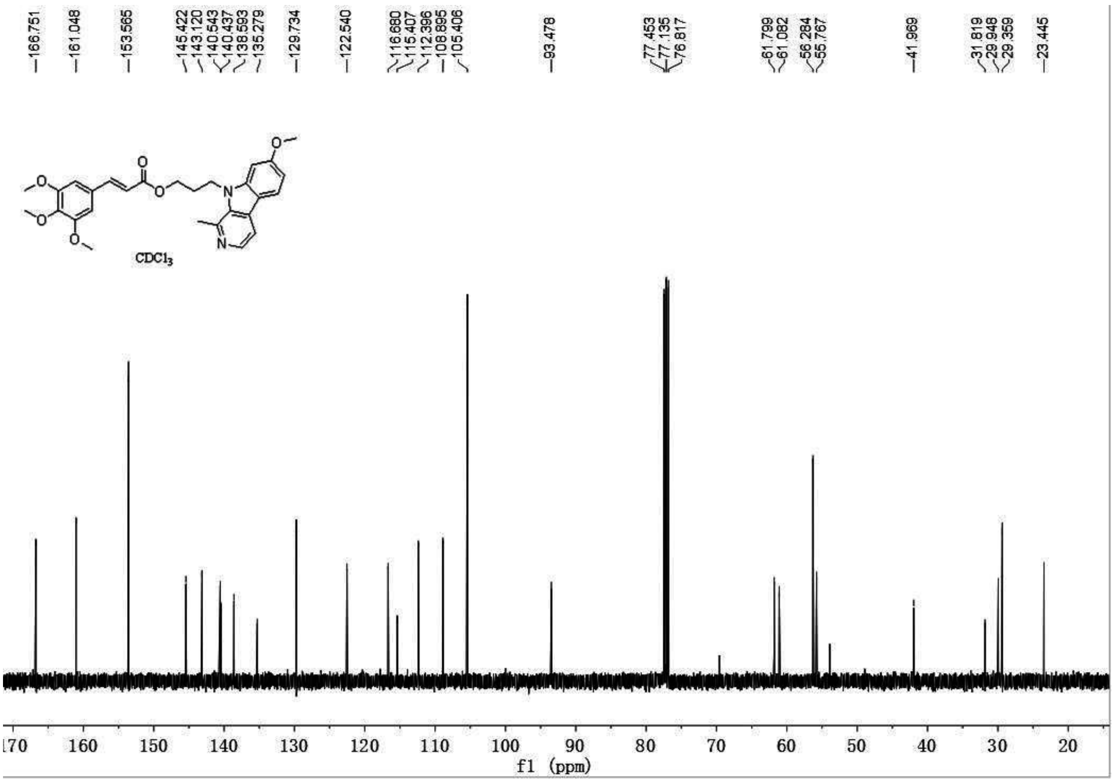

S5: 13C-NMR of 7b

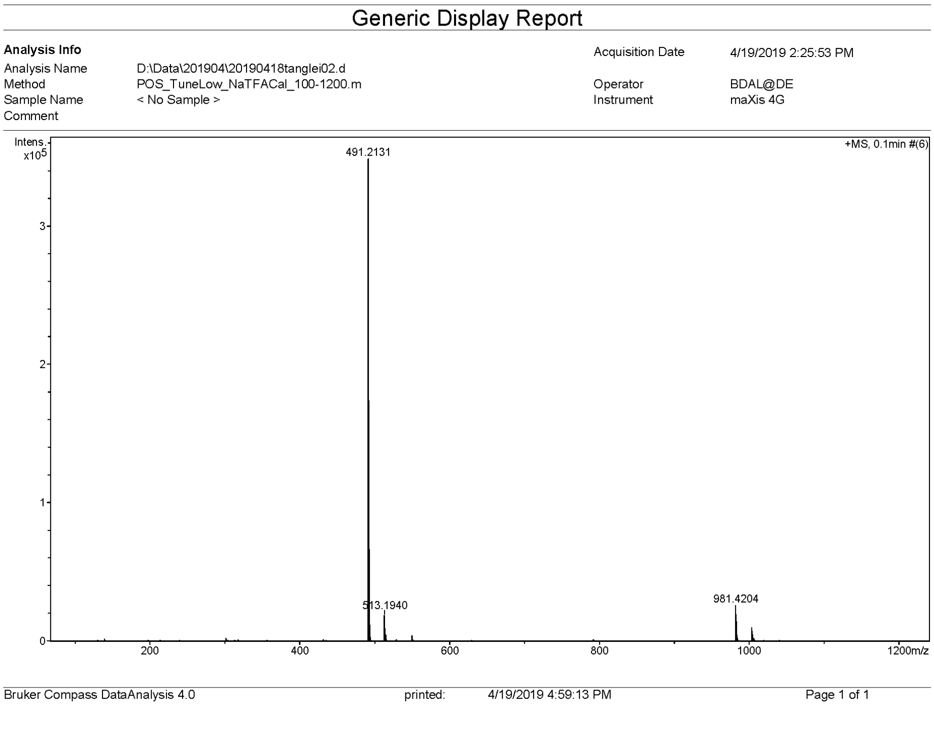

S6: ESI-MS of 7b

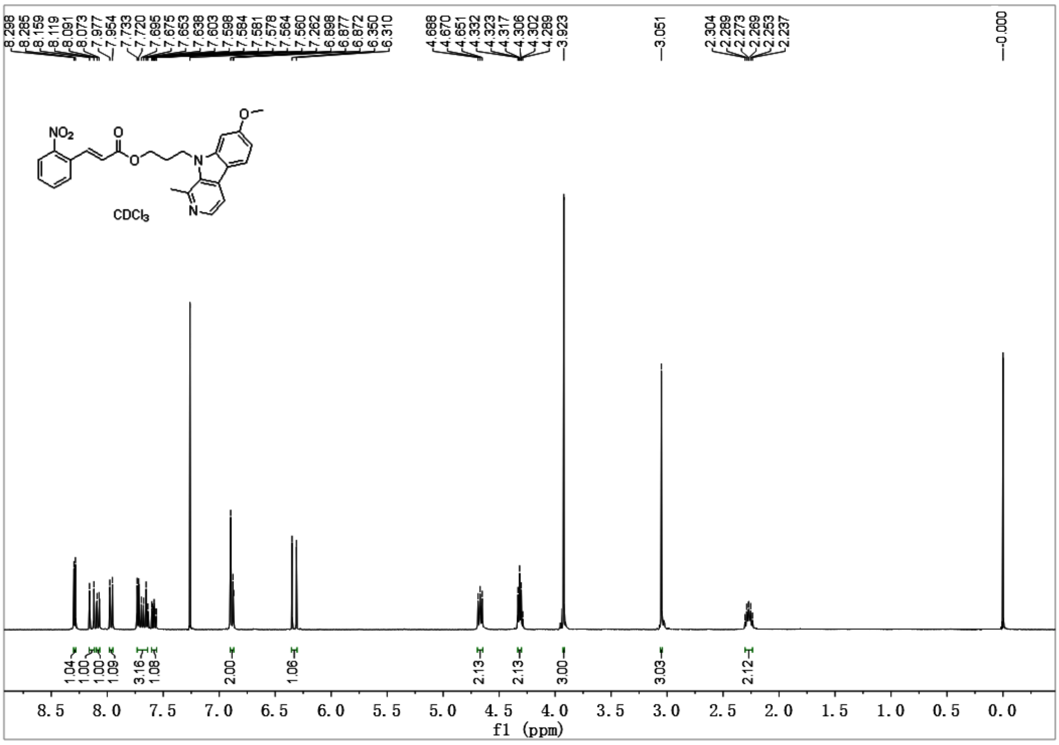

S7: 1H-NMR of 7c

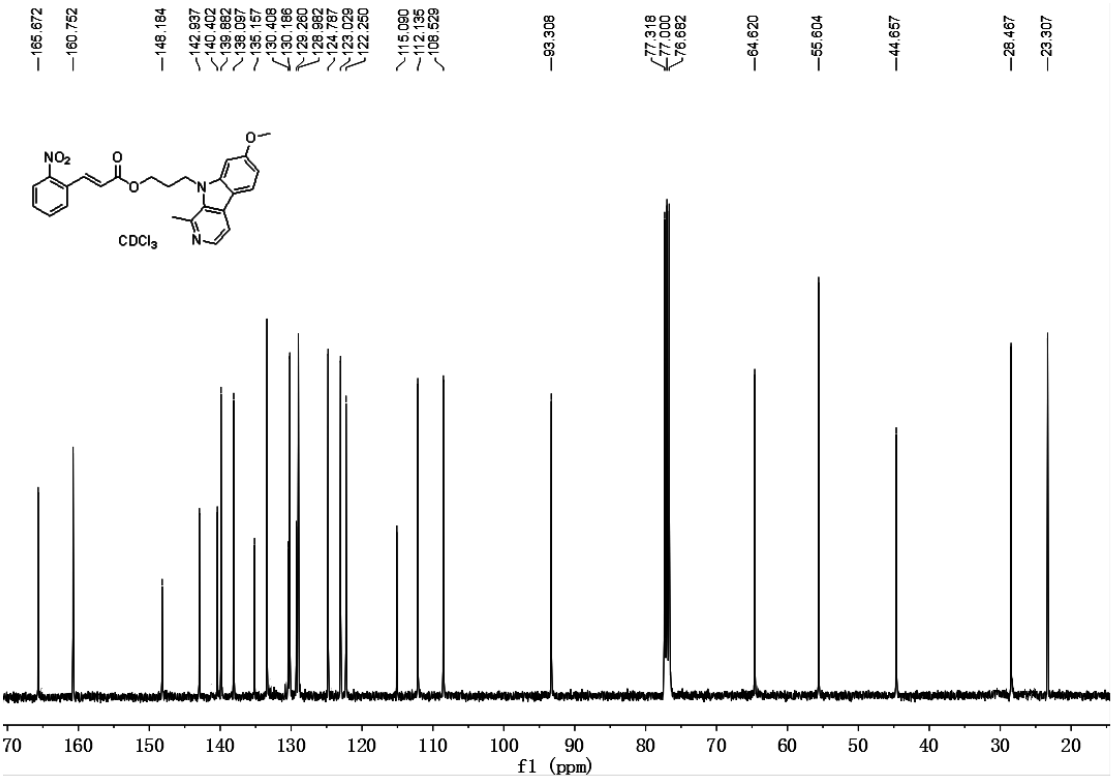

S8: 13C-NMR of 7c

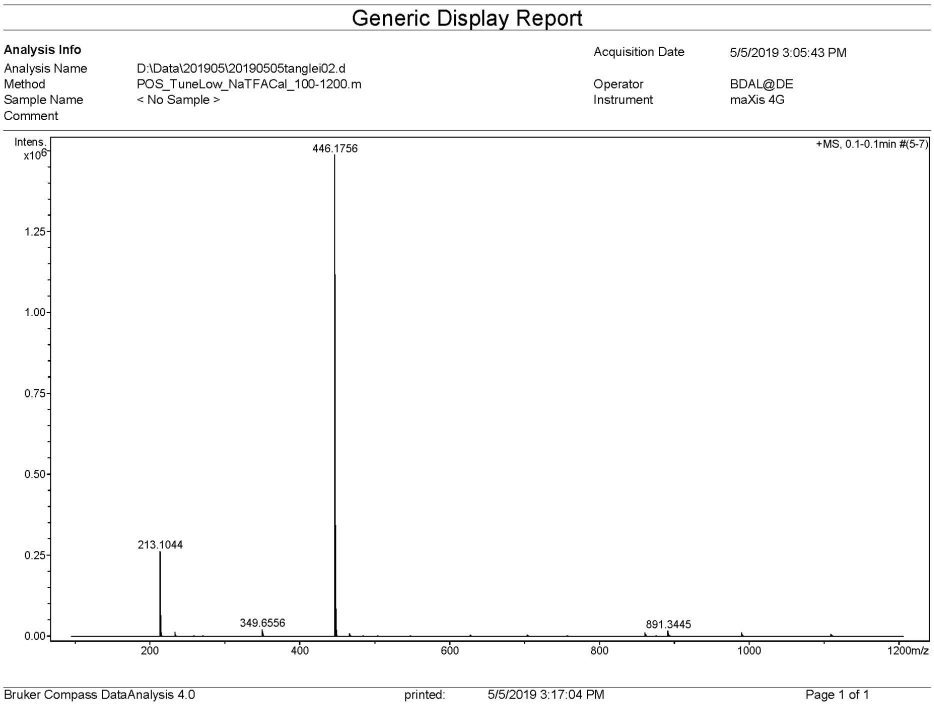

S9: ESI-MS of 7c

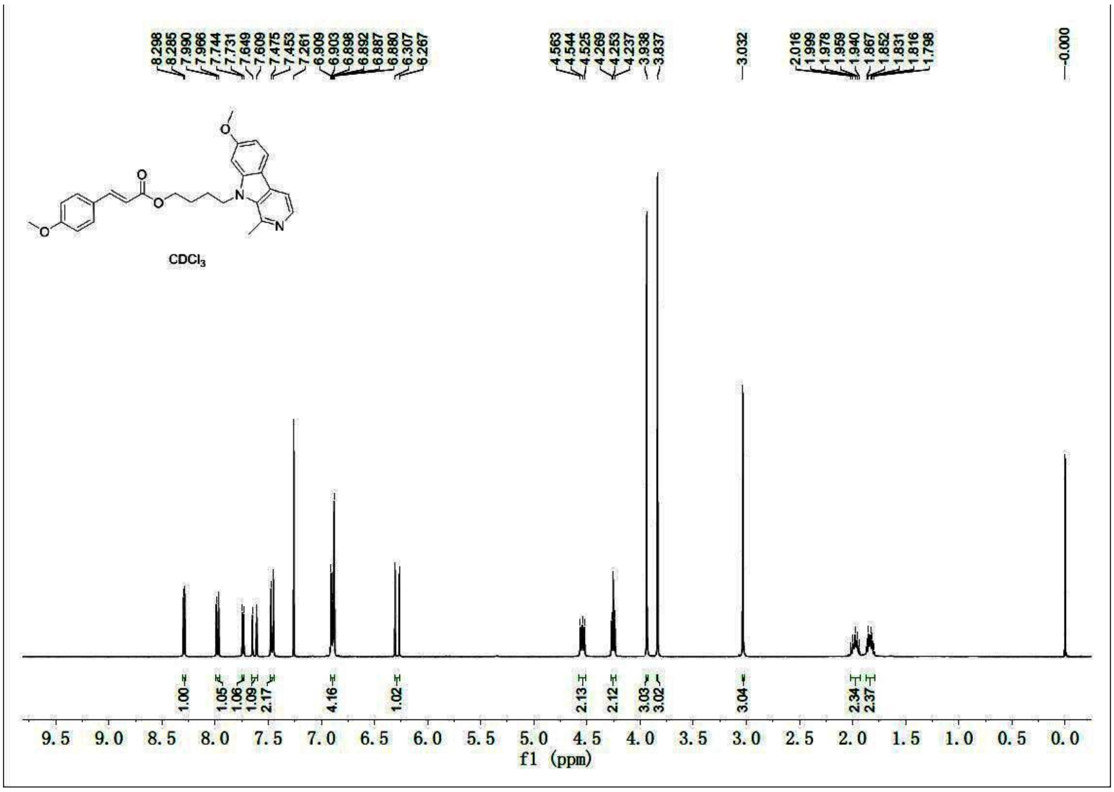

S10: 1H-NMR of 7d

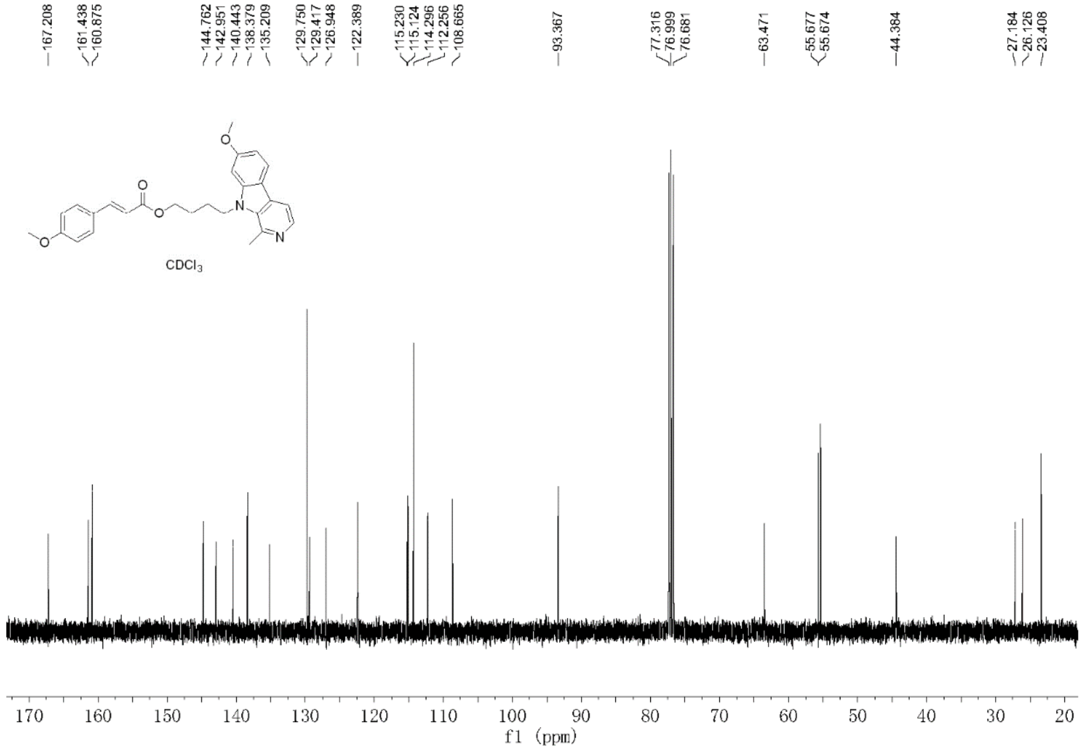

S11: 13C-NMR of 7d

Generic Display Report

Analysis Info		Acquisition Date	3/22/2019 4:14:11 PM
Analysis Name	D:\Data\201903\20190322zhangchenghong05.d		
Method	POS_TuneLow_NaTFACal_100-1200.m	Operator	BDAL@DE
Sample Name	< No Sample >	Instrument	maXis 4G
Comment			

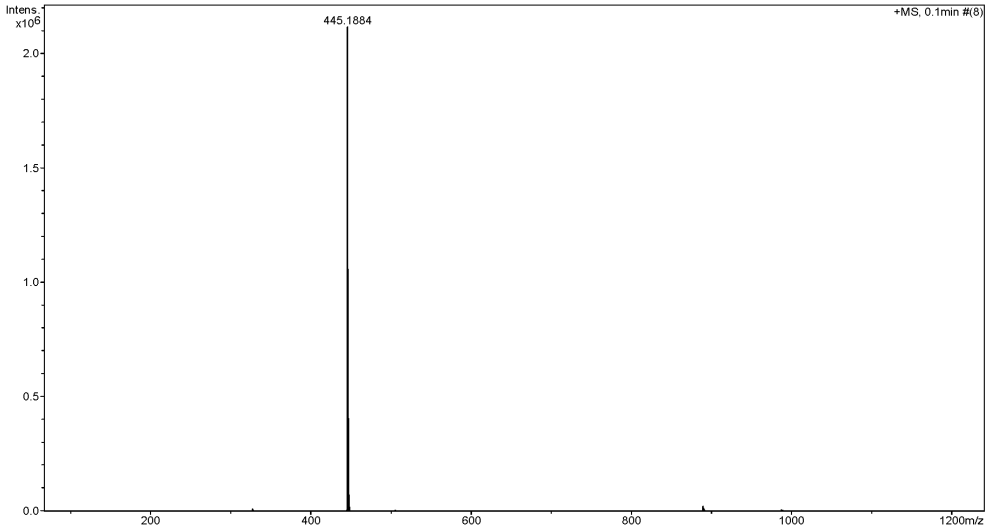

Bruker Compass DataAnalysis 4.0 printed: 3/22/2019 4:29:22 PM Page 1 of 1

S12: ESI-MS of 7d

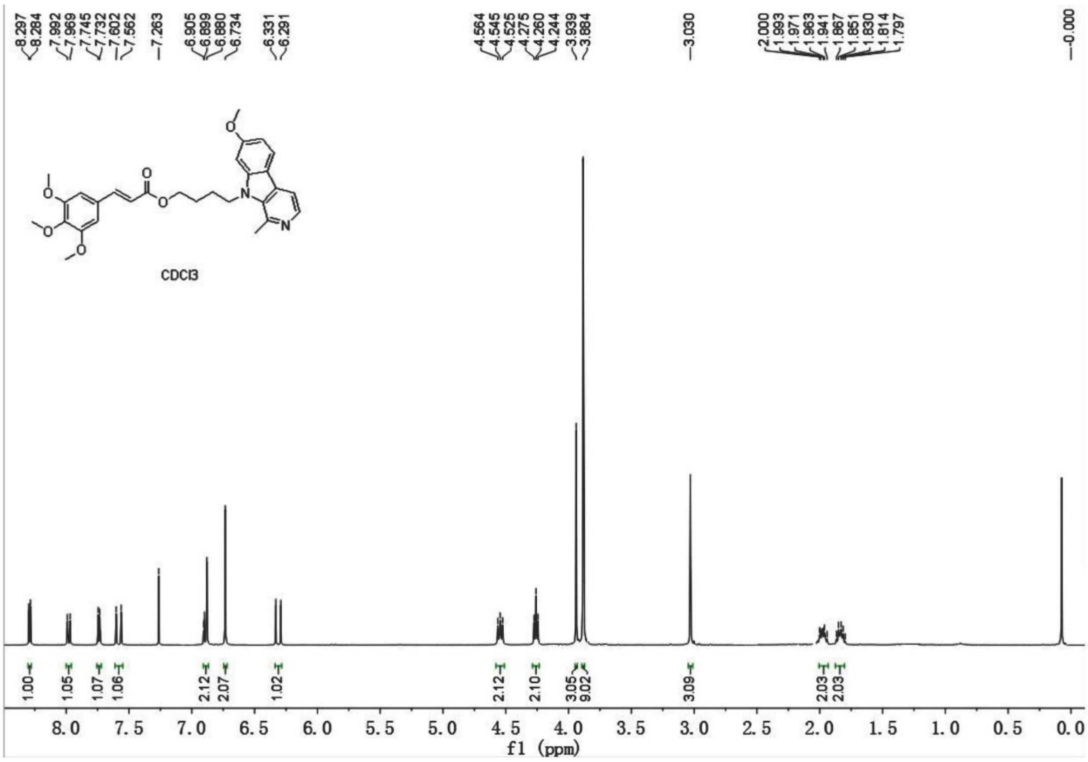

S13: 1H-NMR of 7e

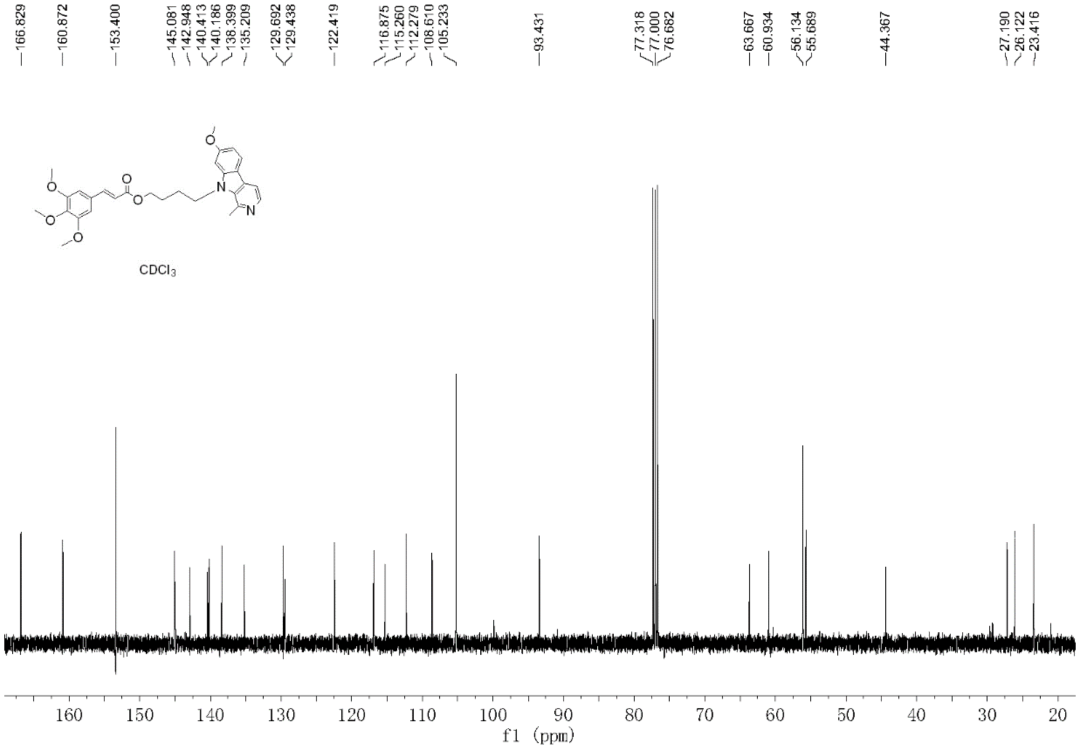

S14: 13C-NMR of 7e

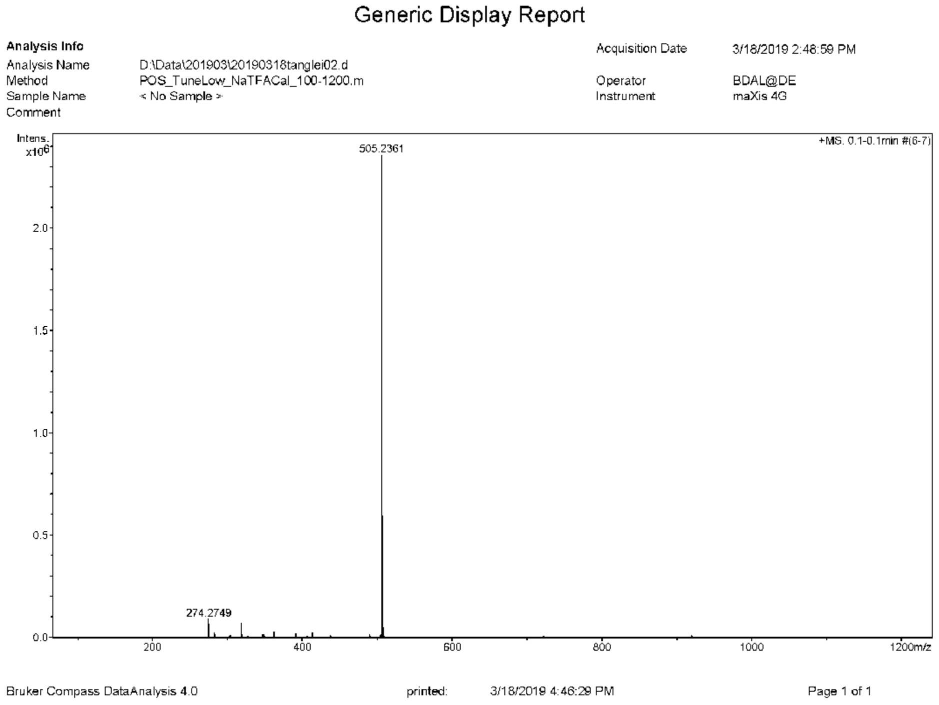

S15: ESI-MS of 7e

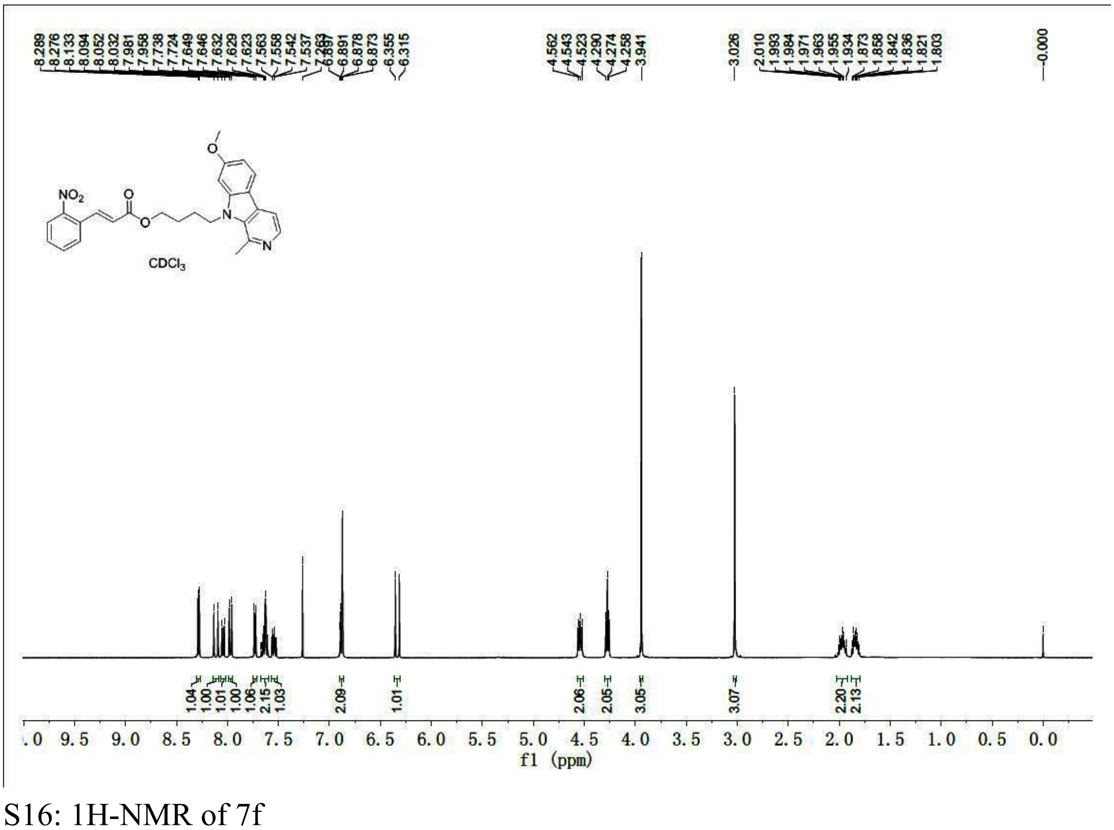

S16: 1H-NMR of 7f

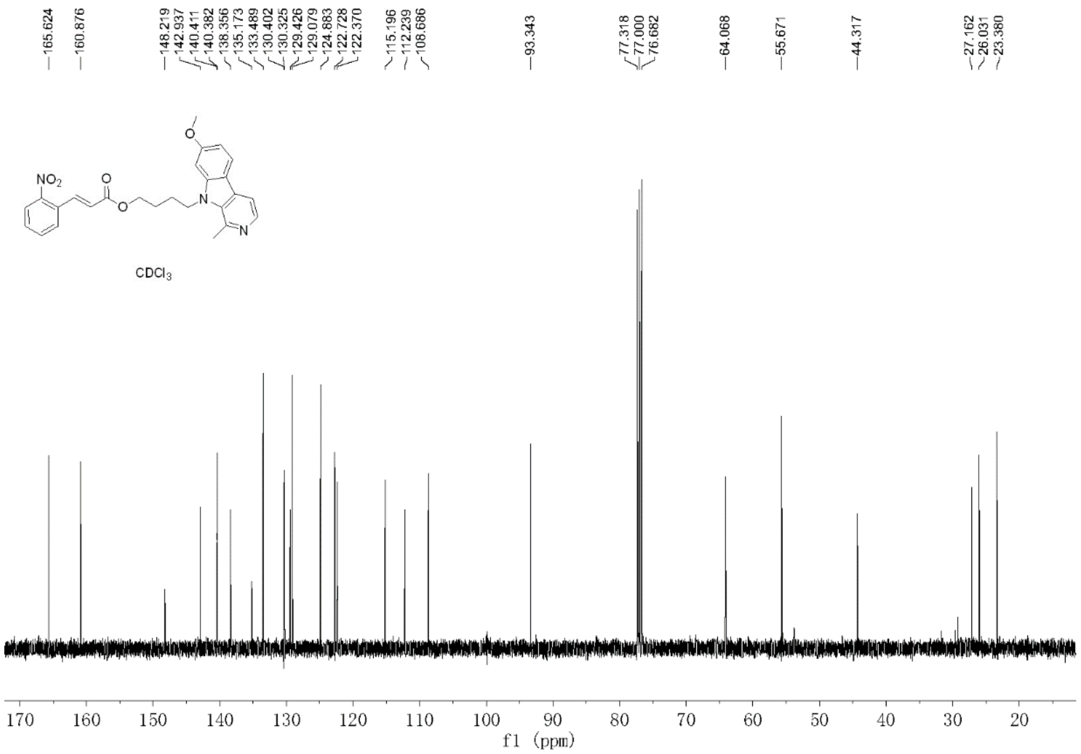

S17: 13C-NMR of 7f

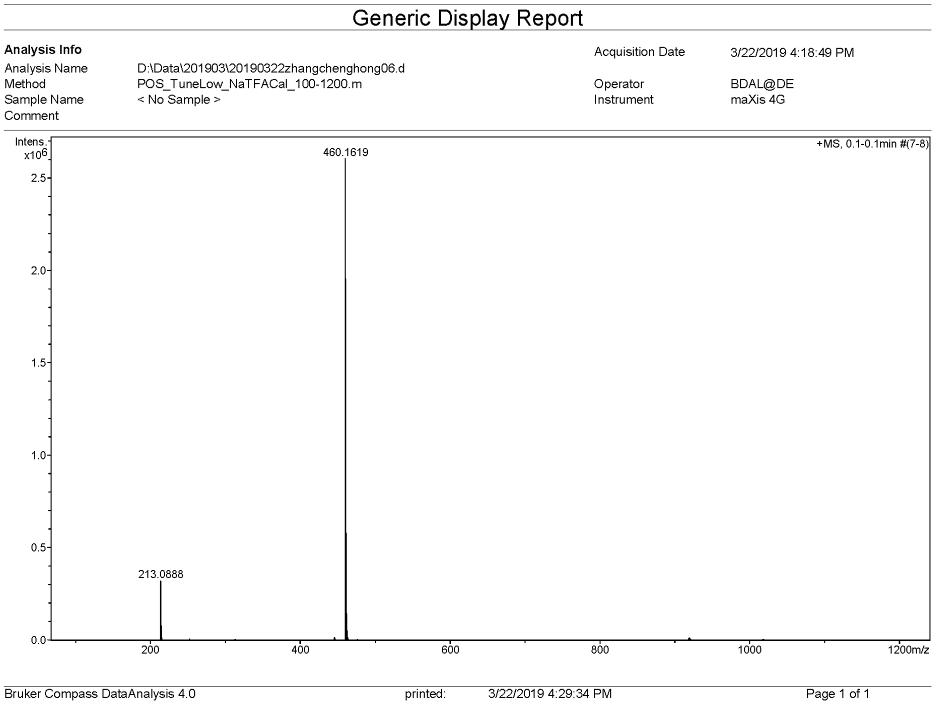

S18: ESI-MS of 7f

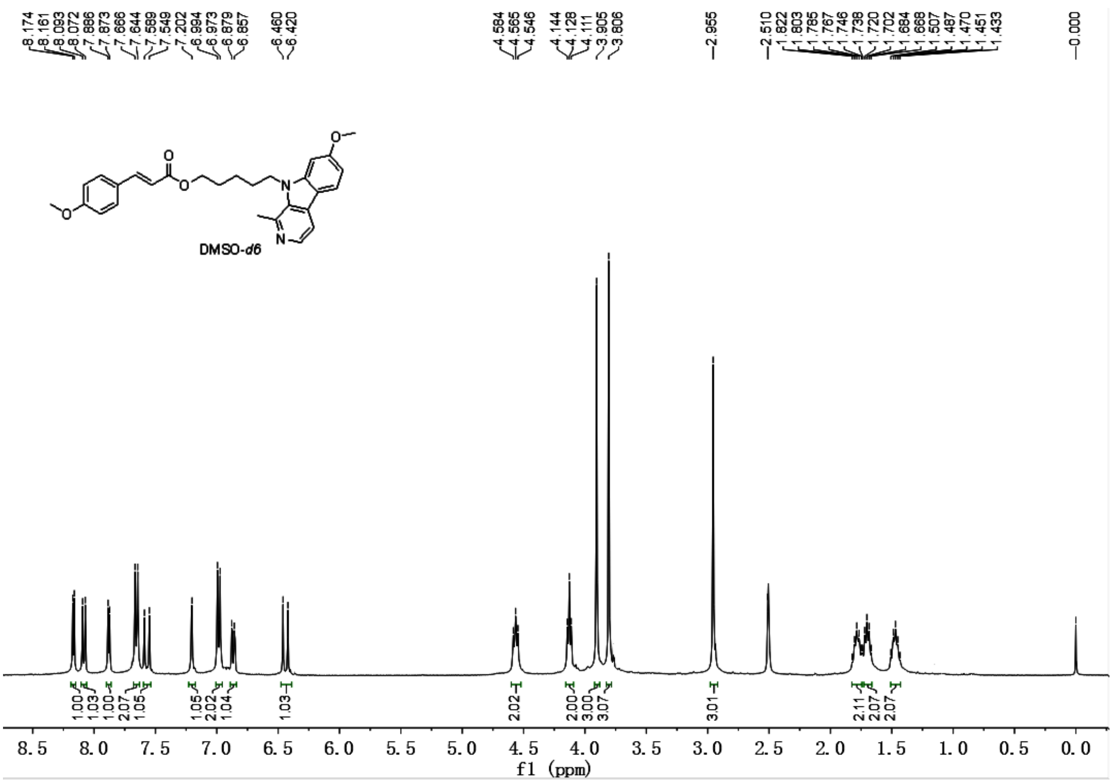

S19: 1H-NMR of 7g

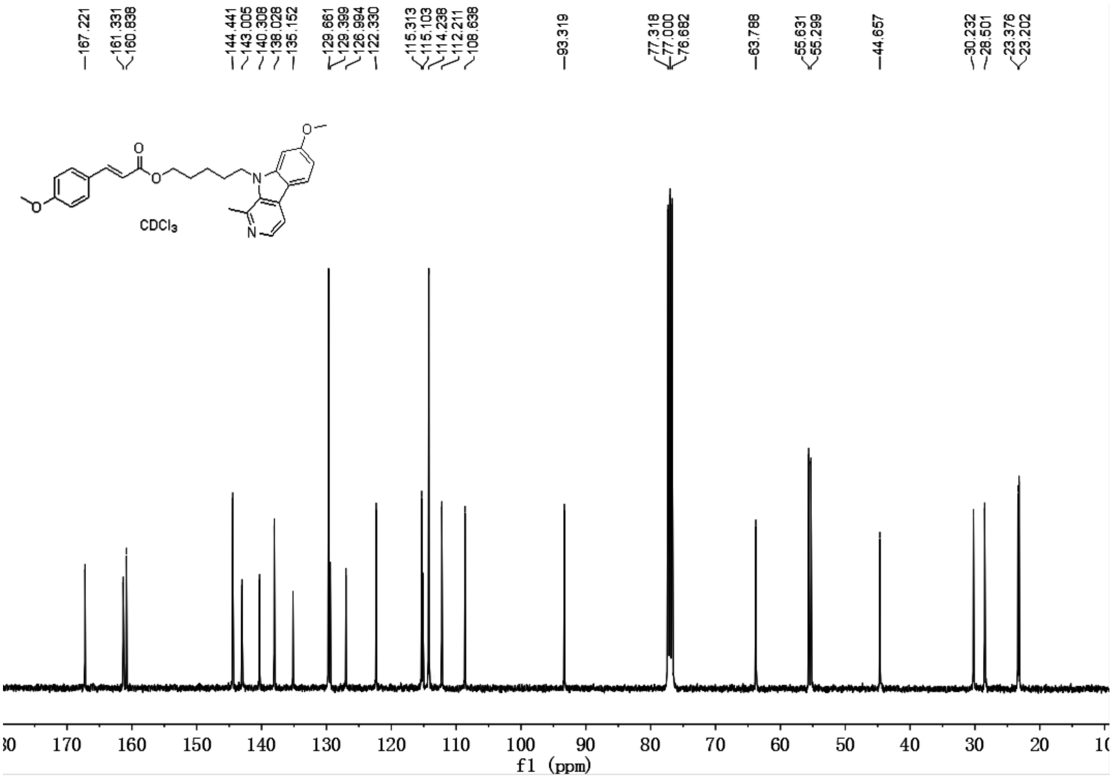

S20: 13C-NMR of 7g

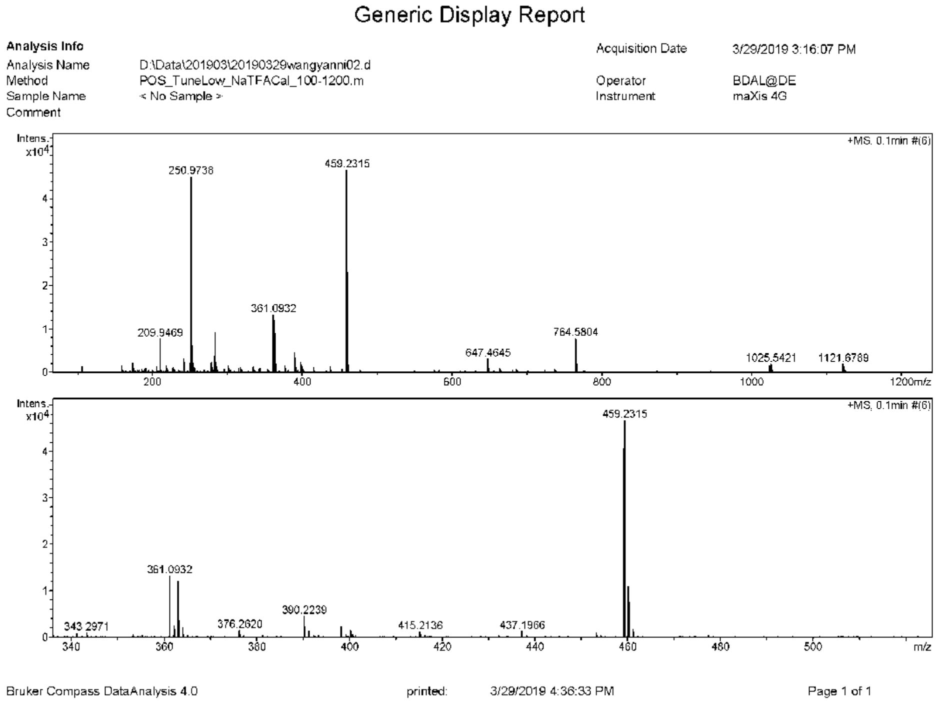

S21: ESI-MS of 7g

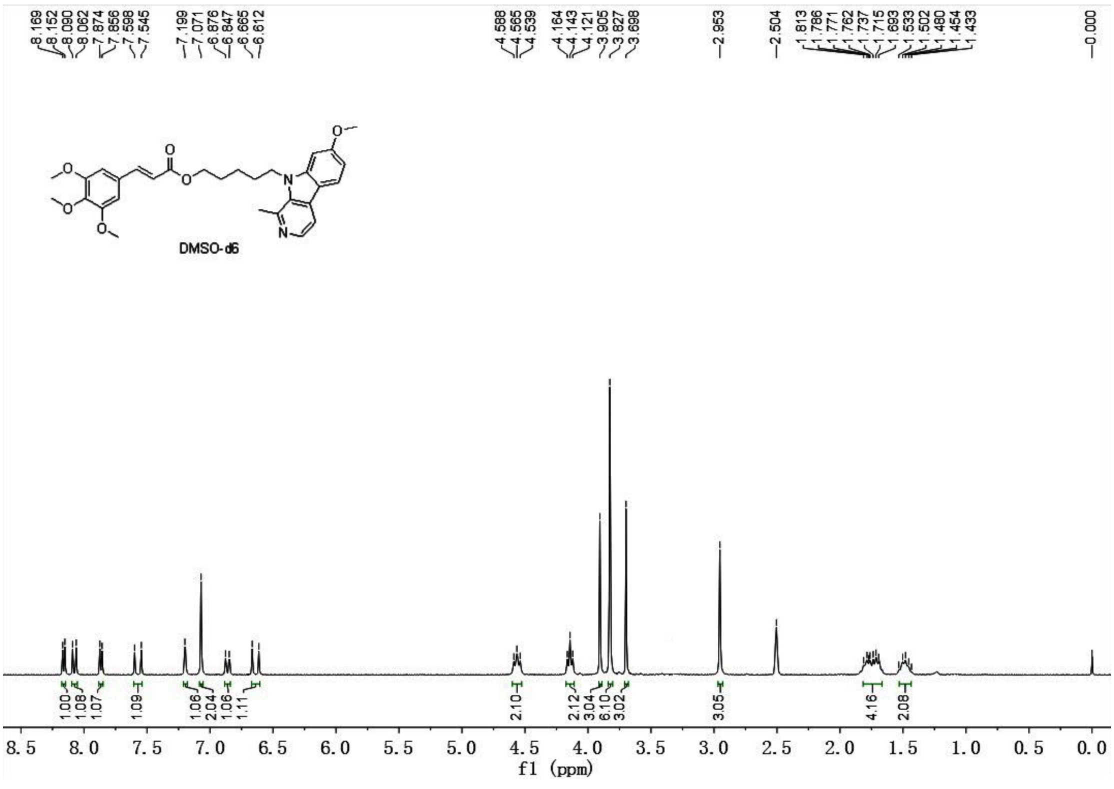

S22: 1H-NMR of 7h

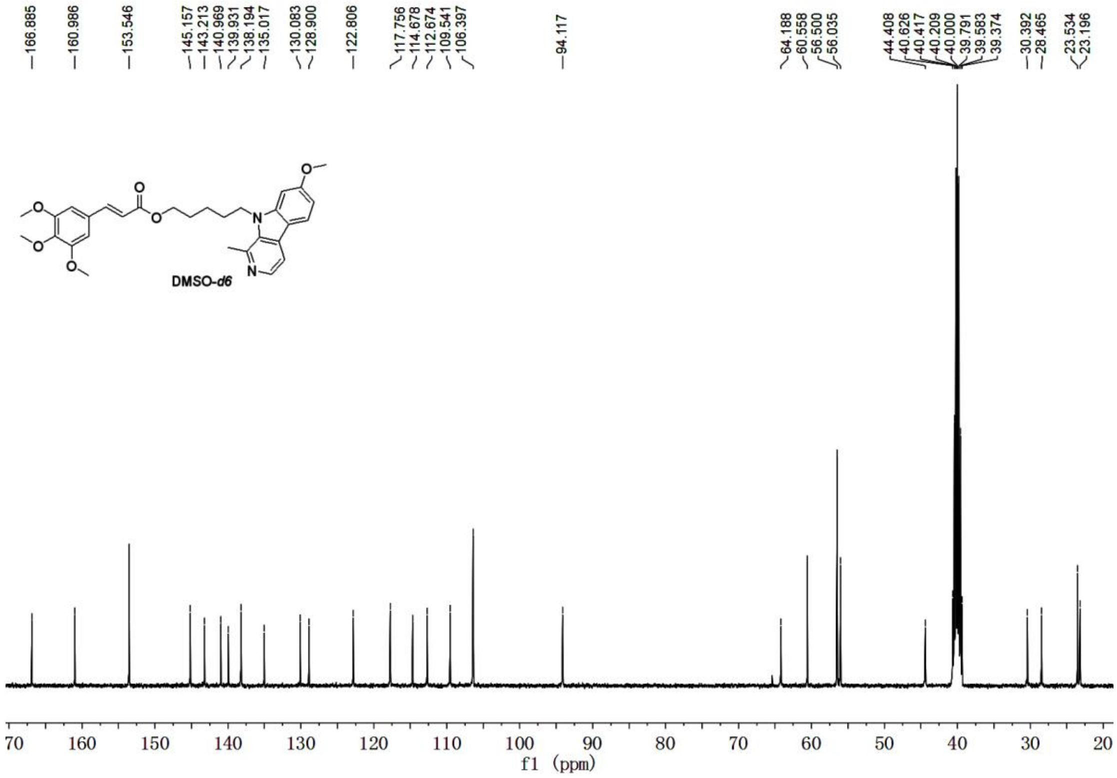

S23: 13C-NMR of 7h

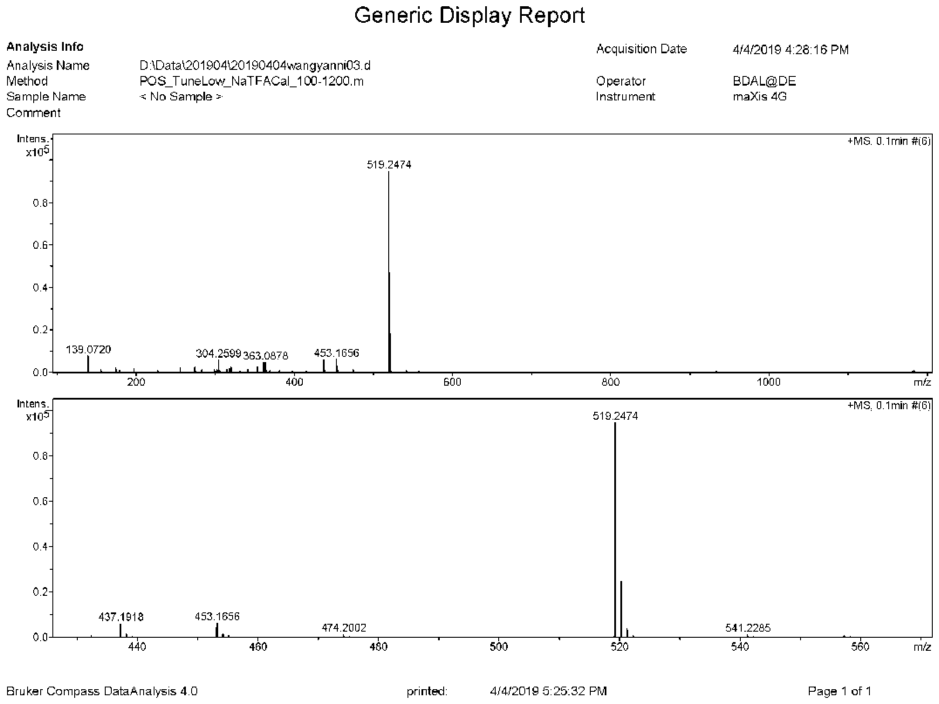

S24: ESI-MS of 7h

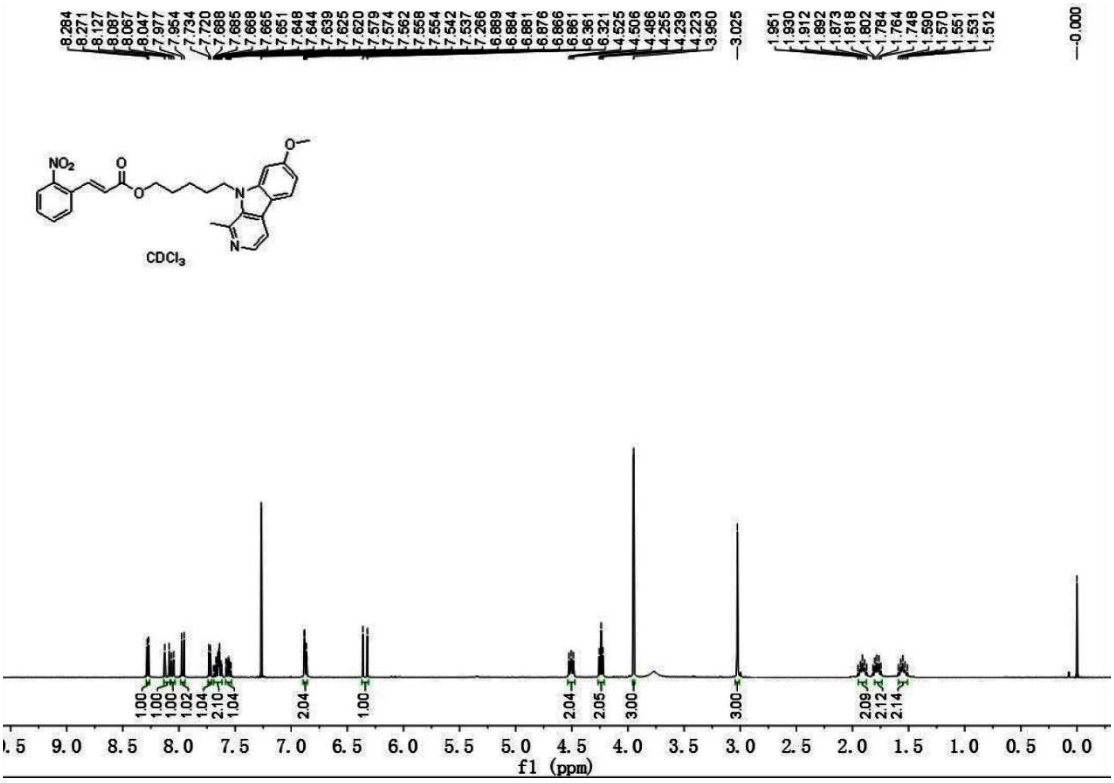

S25: 1H-NMR of 7i

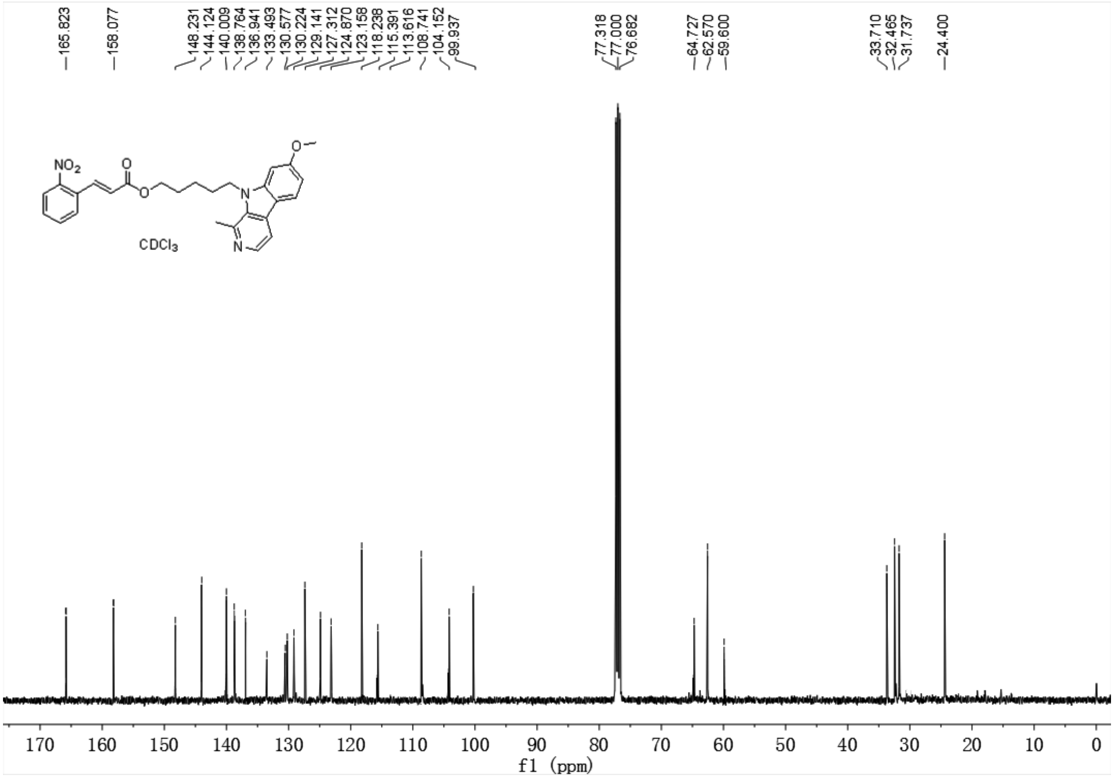

S26: 13C-NMR of 7i

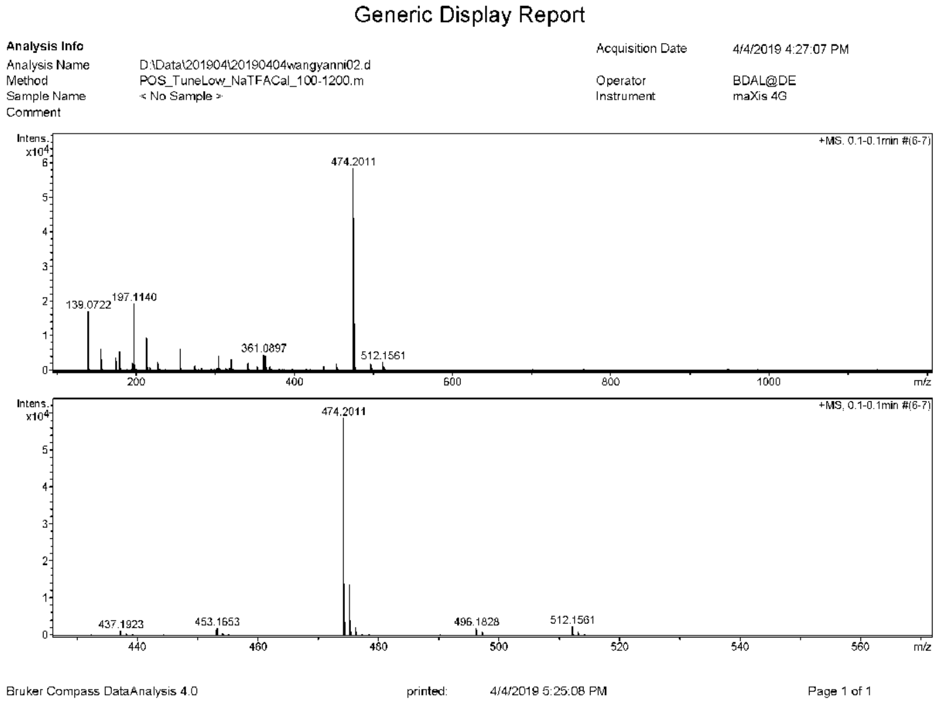

S27: ESI-MS of 7i

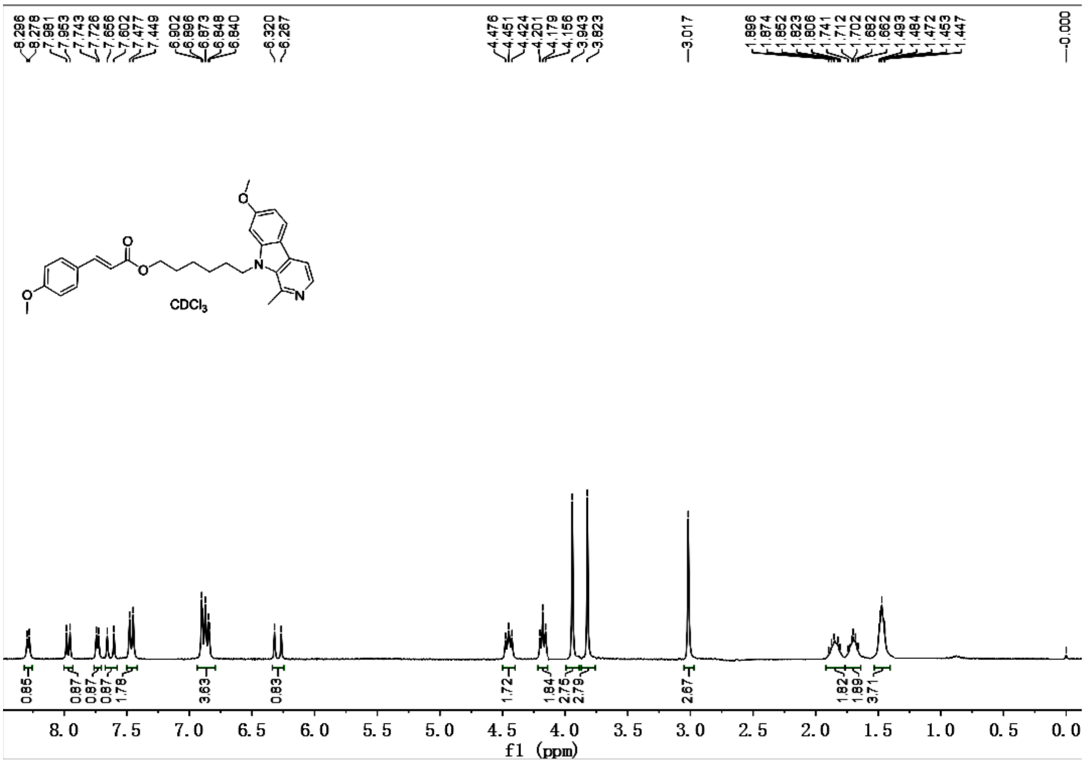

S28: 1H-NMR of 7j

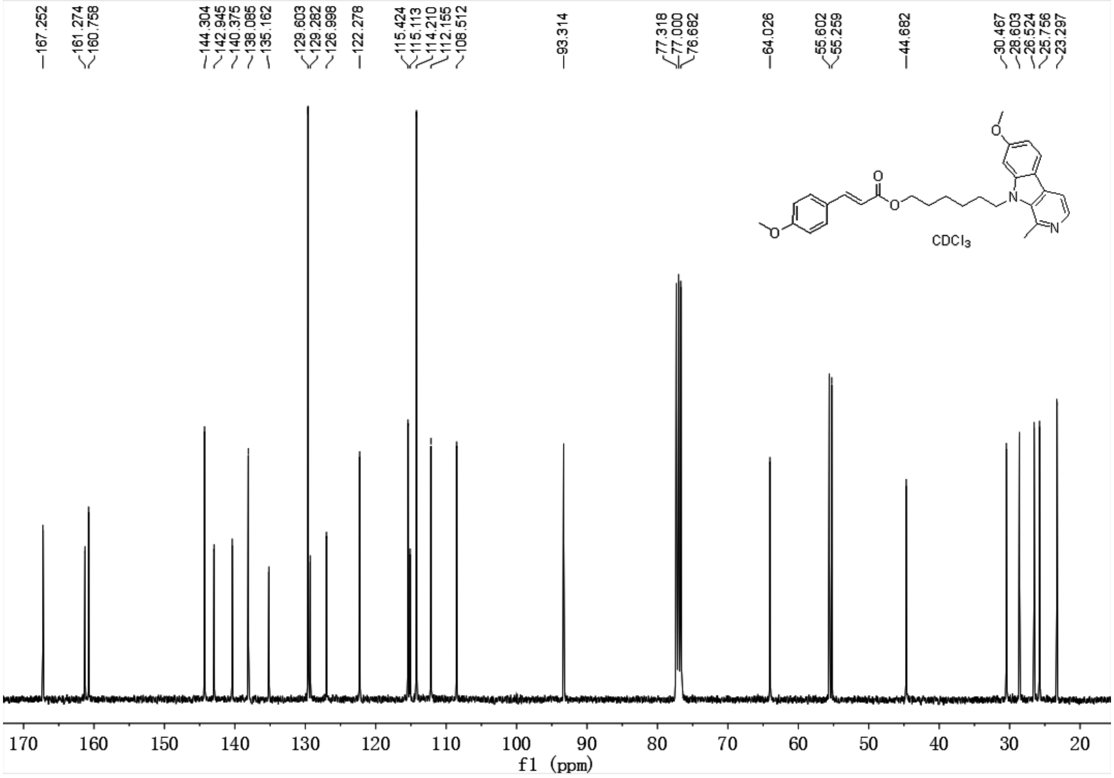

S29: 13C-NMR of 7j

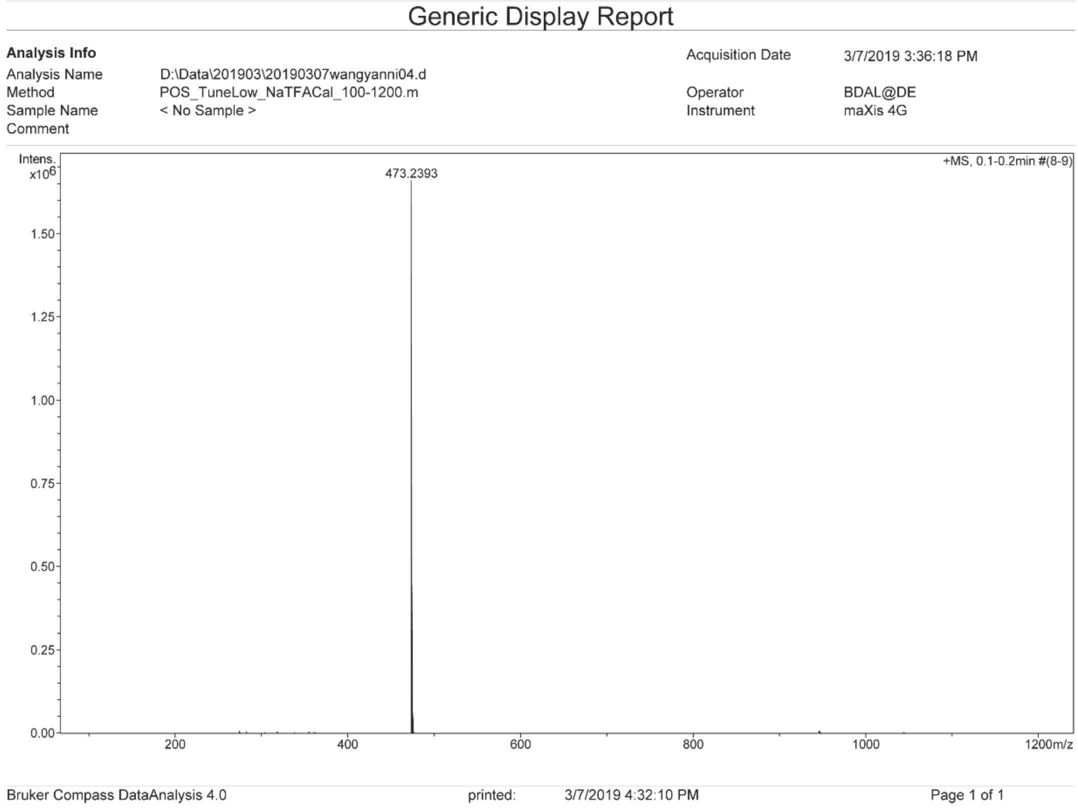

S30: ESI-MS of 7j

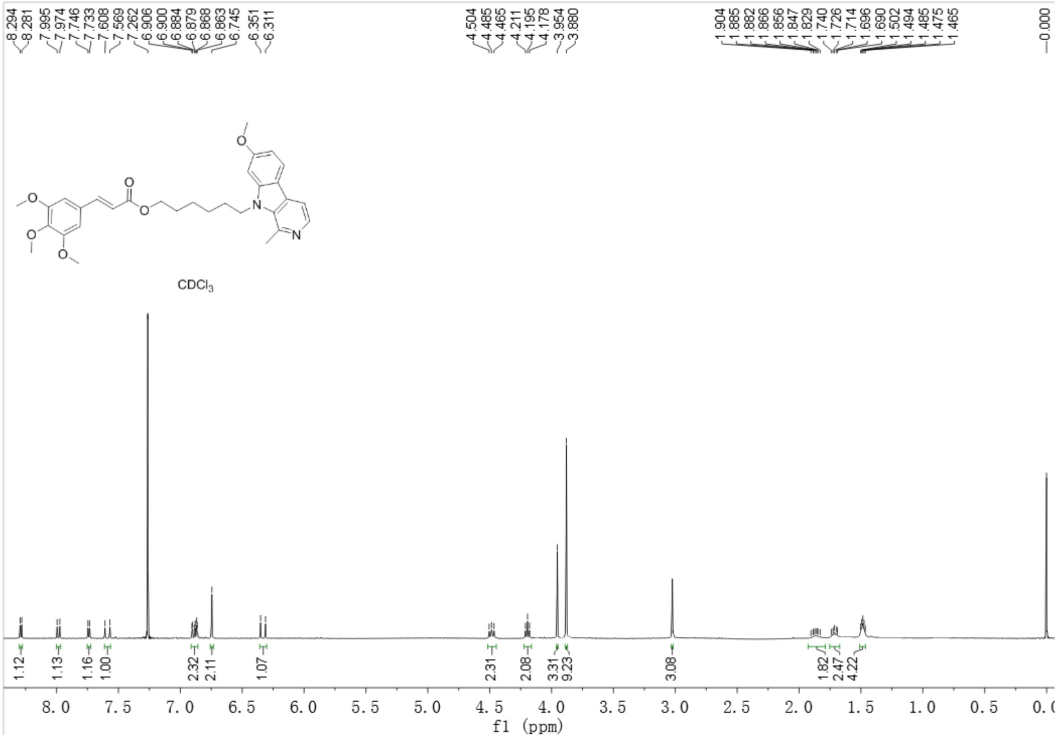

S31: 1H-NMR of 7k

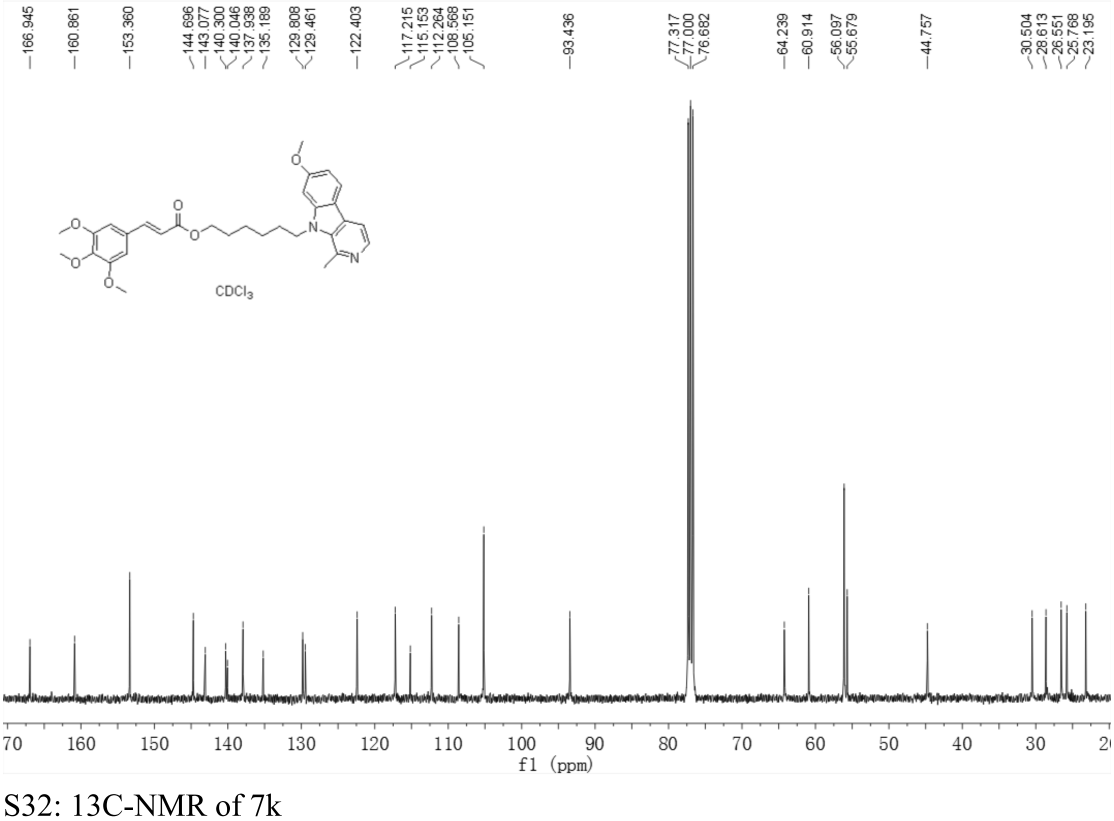

S32: 13C-NMR of 7k

Generic Display Report

Analysis Info

Analysis Name	D:\Data\201903\20190313tanglei04.d	Acquisition Date	3/13/2019 12:01:06 PM
Method	POS_TuneLow_NaTFACal_100-1200.m	Operator	BDAL@DE
Sample Name	< No Sample >	Instrument	maXis 4G
Comment			

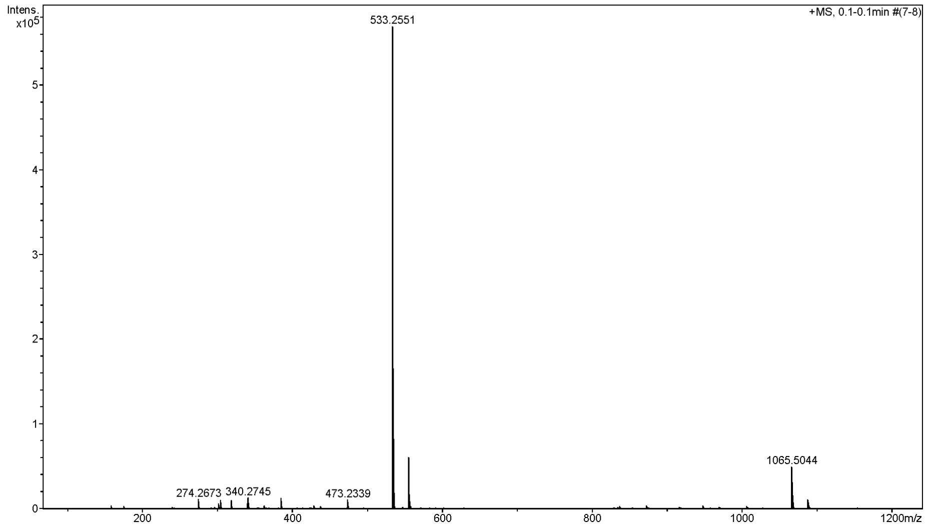

Bruker Compass DataAnalysis 4.0 printed: 3/13/2019 12:34:51 PM Page 1 of 1

S33: ESI-MS of 7k

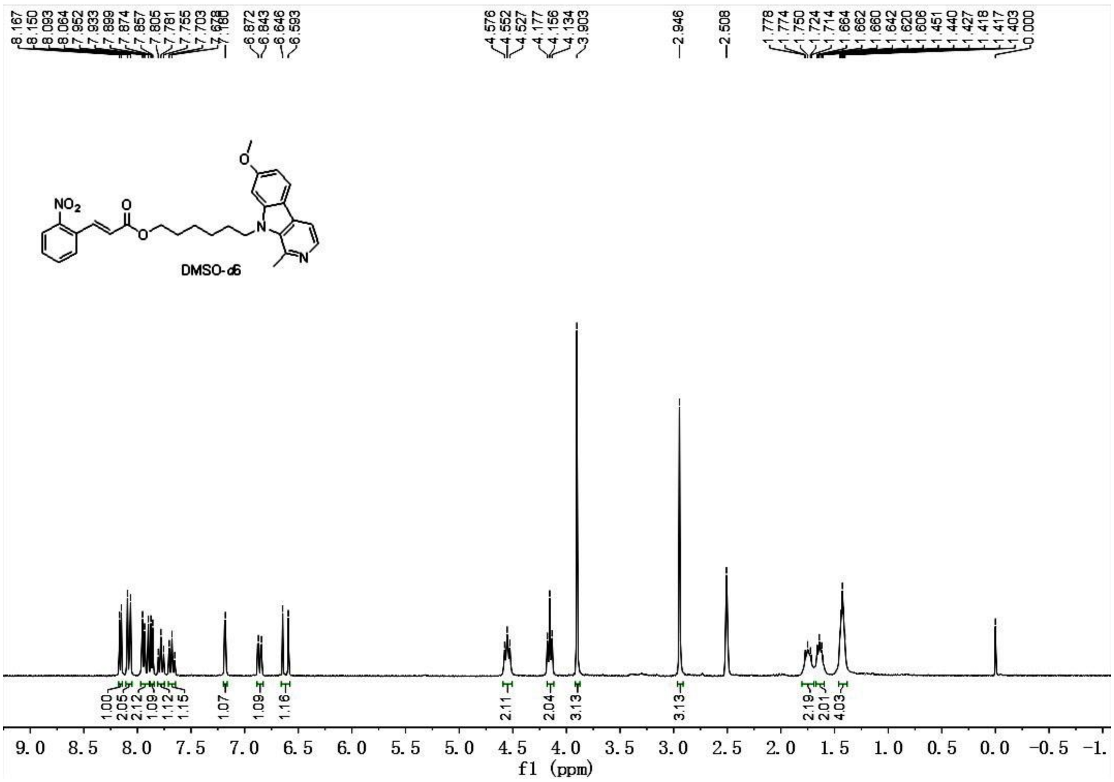

S34: 1H-NMR of 7l

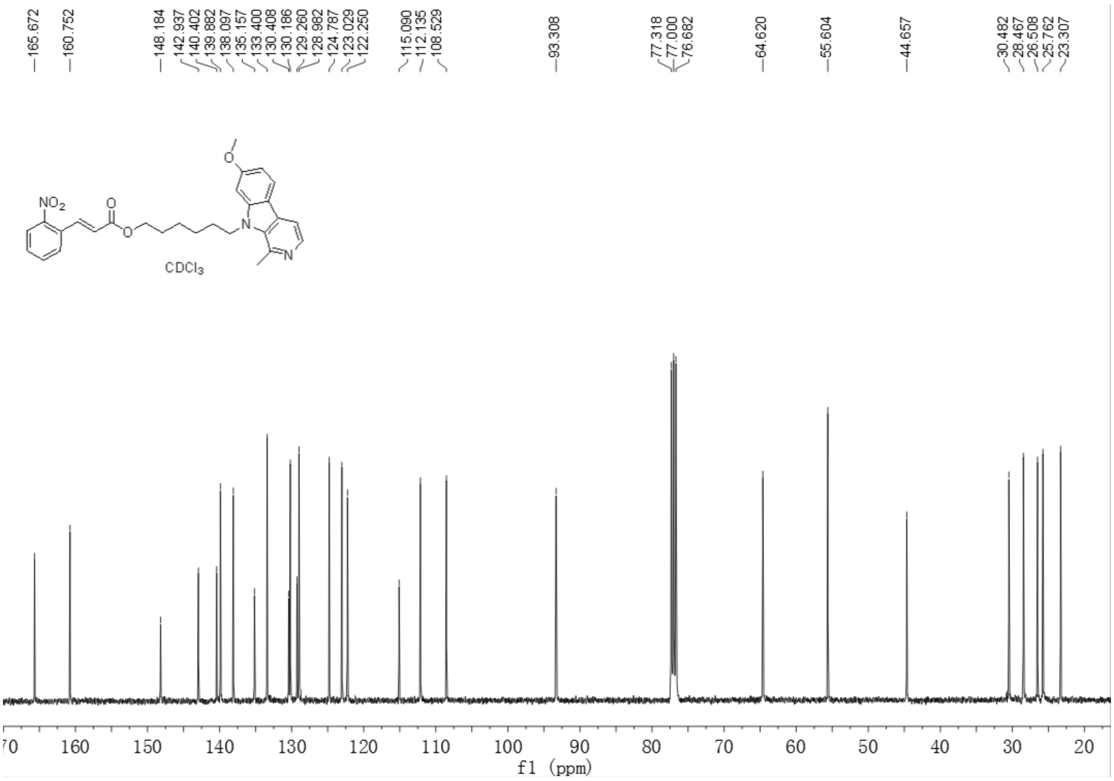

S35: 13C-NMR of 7l

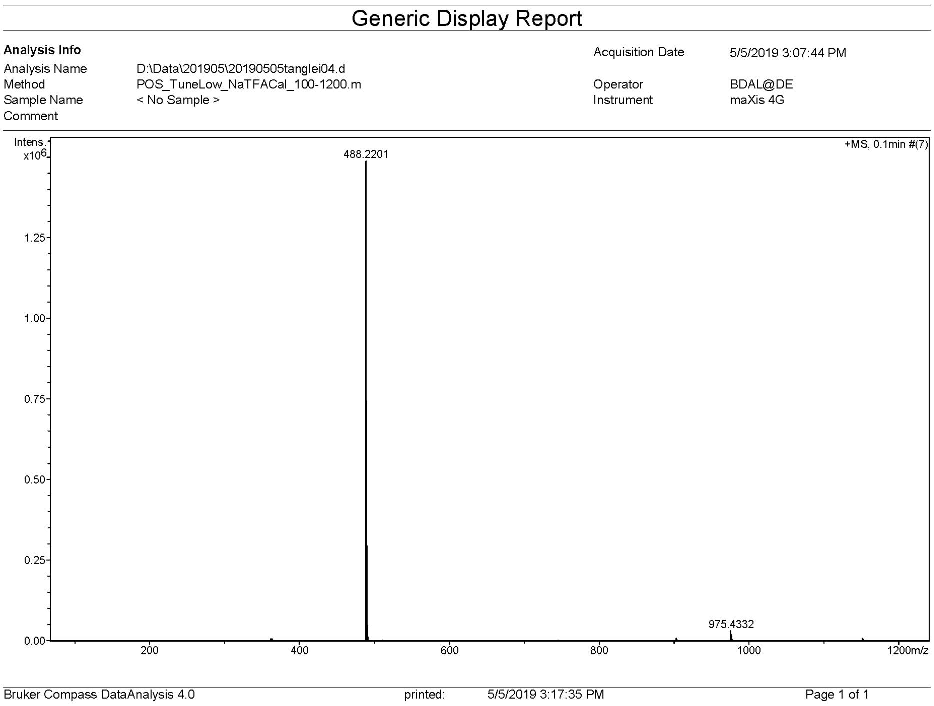

S36: ESI-MS of 7l

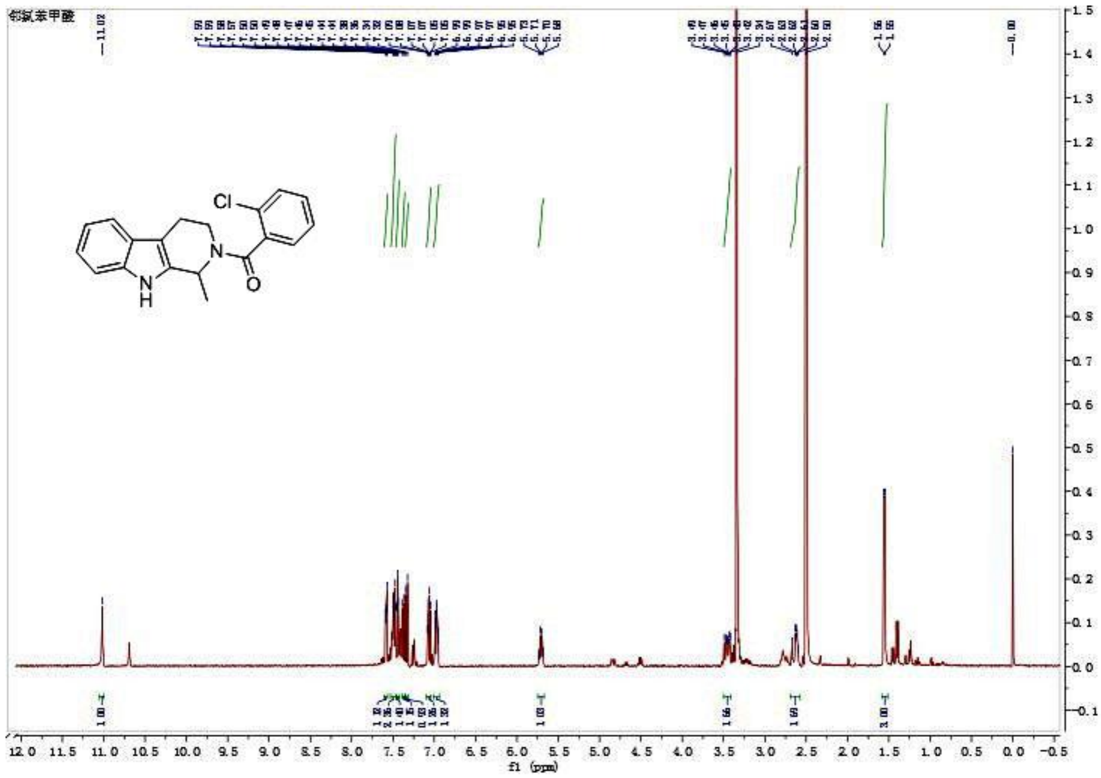

S37: 1H-NMR of 11a

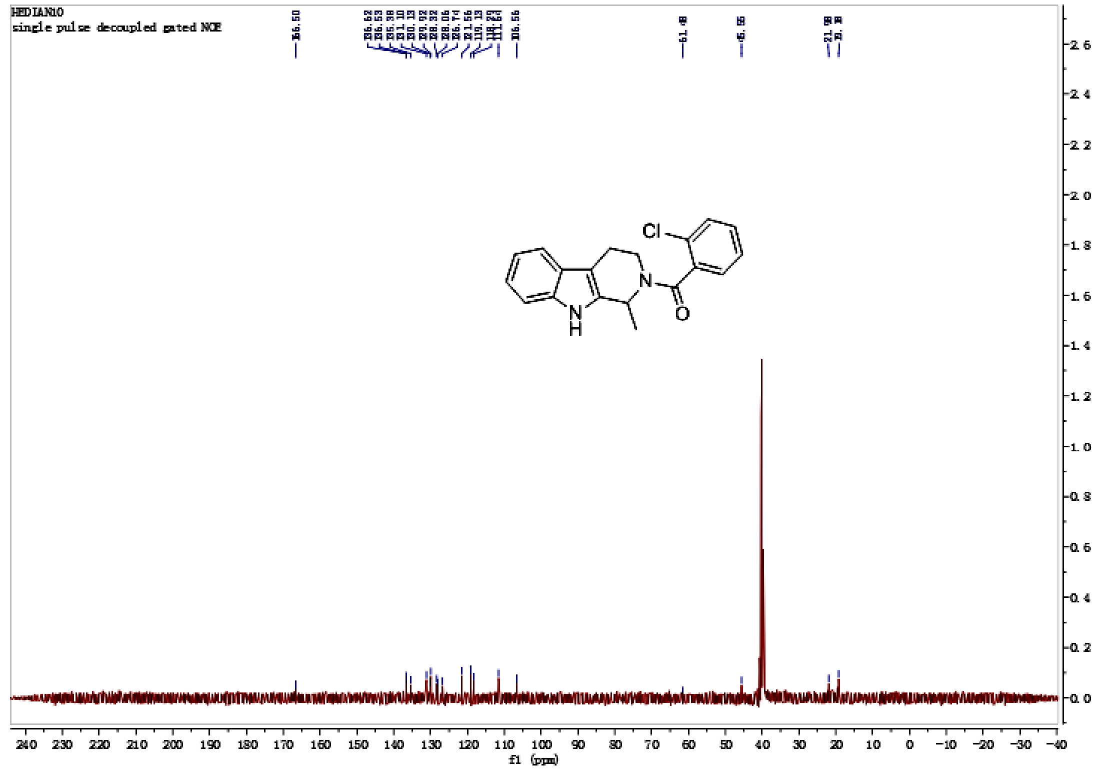

S38: 13C-NMR of 11a

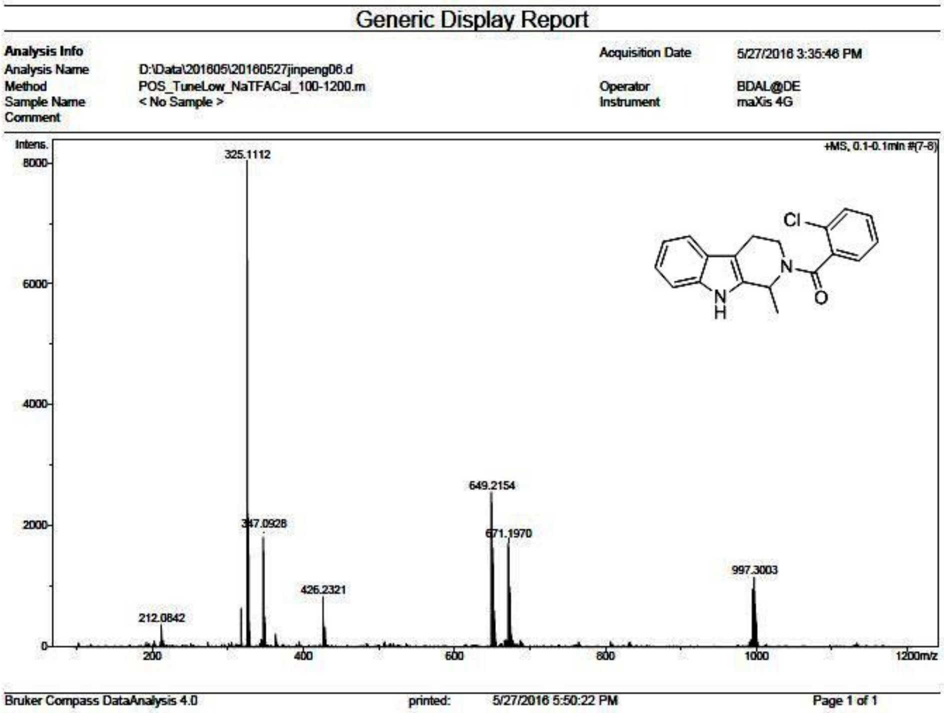

S39: ESI-MS of 11a

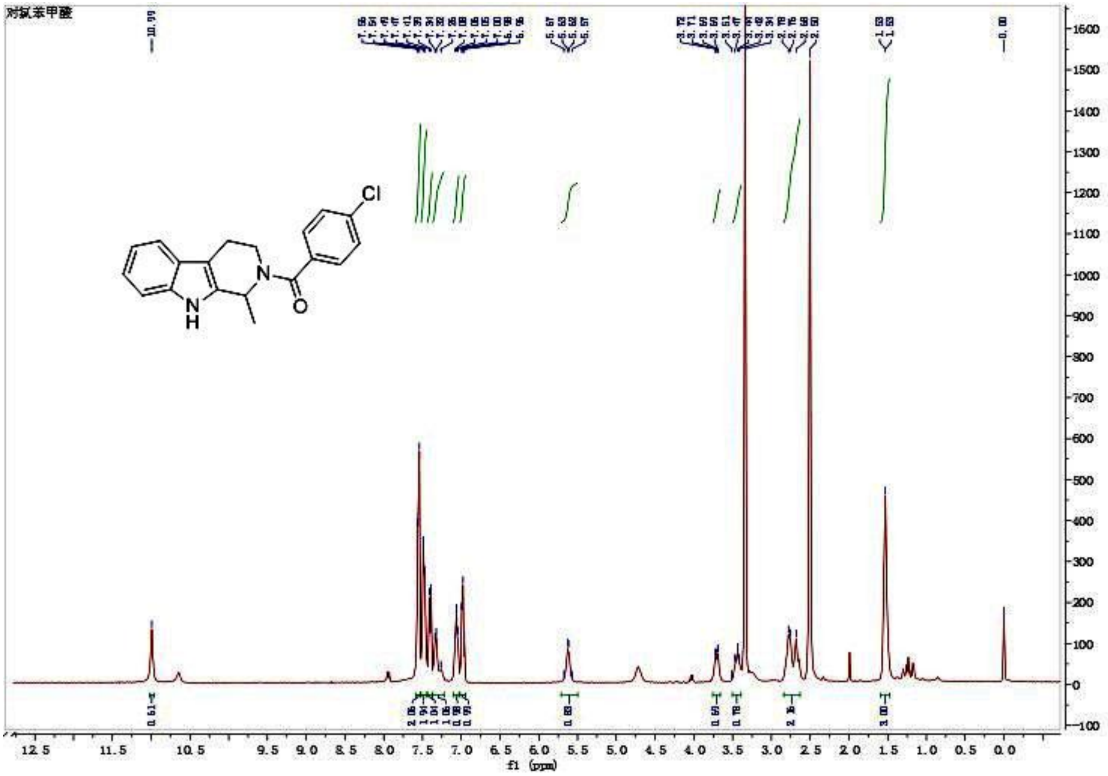

S40: 1H-NMR of 11b

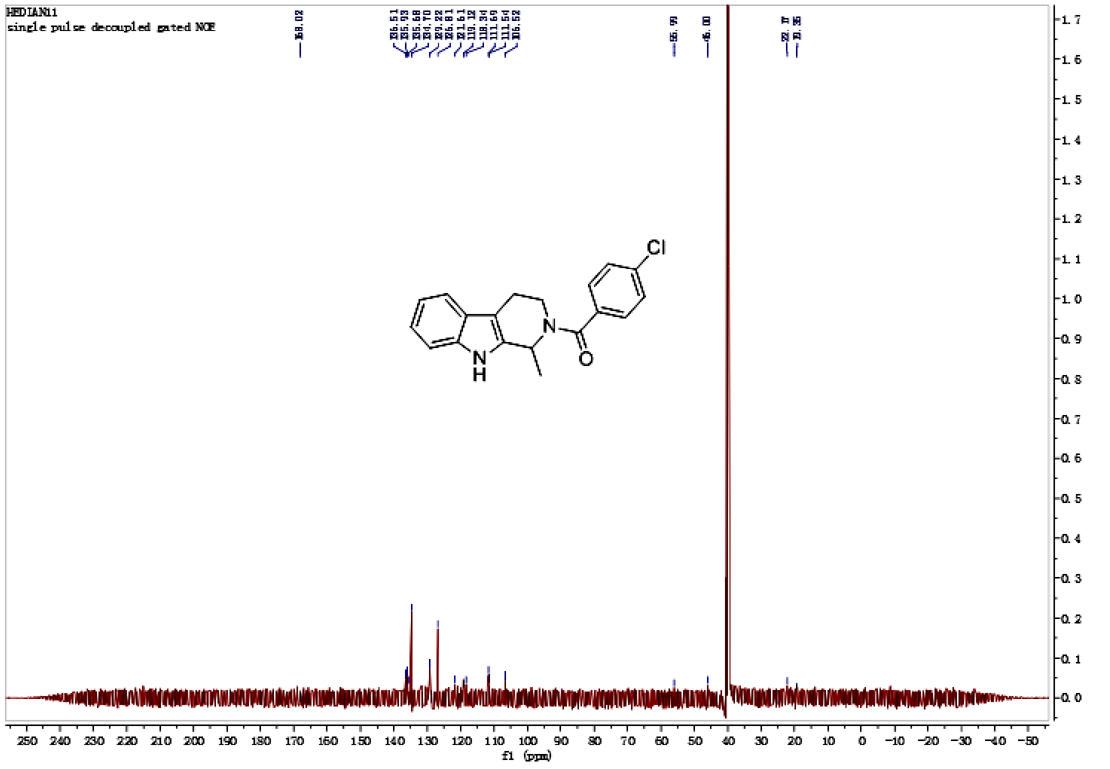

S41: 13C-NMR of 11b

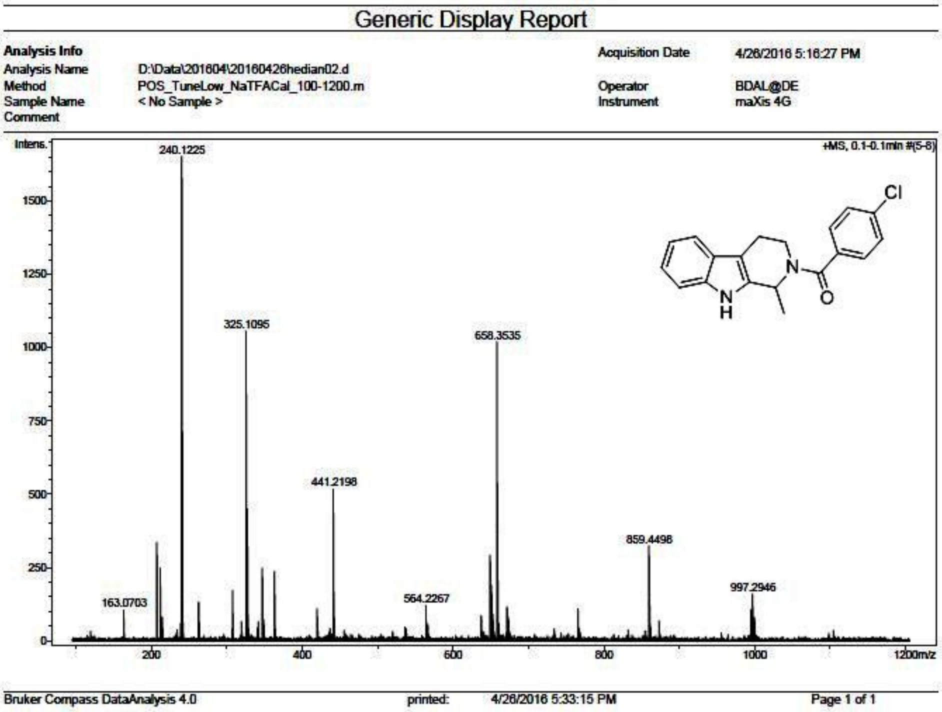

S42: ESI-MS of 11b

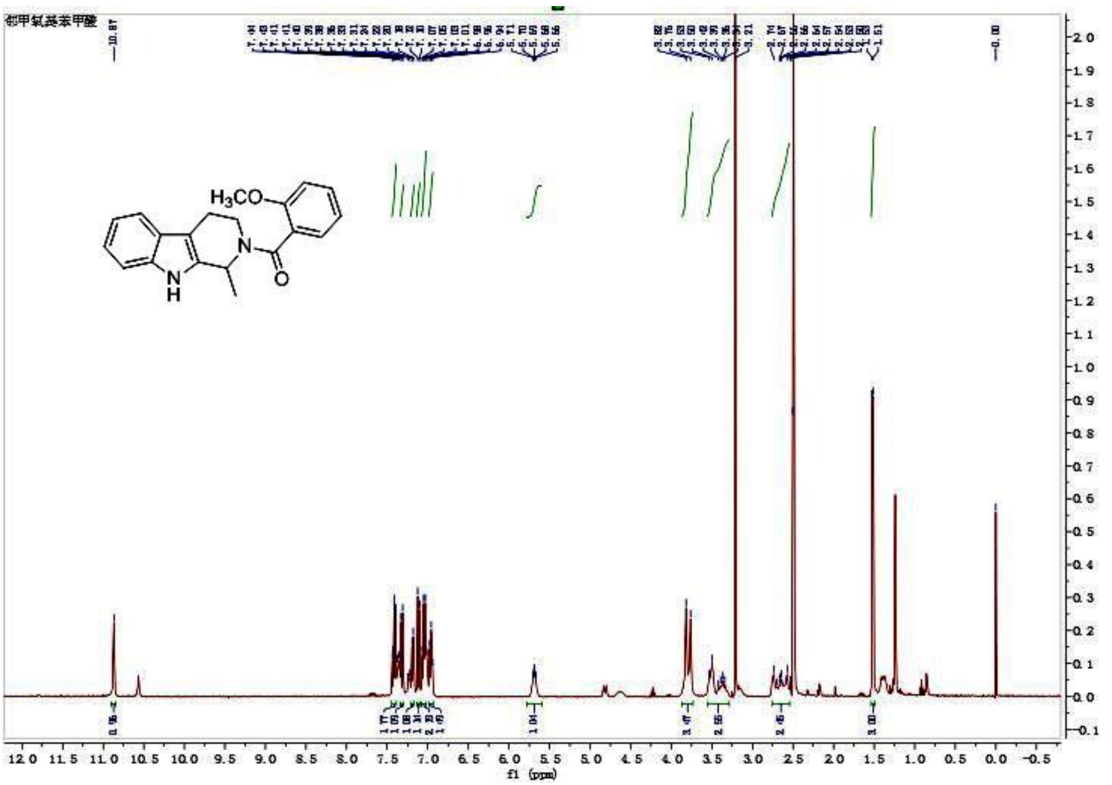

S43: 1H-NMR of 11c

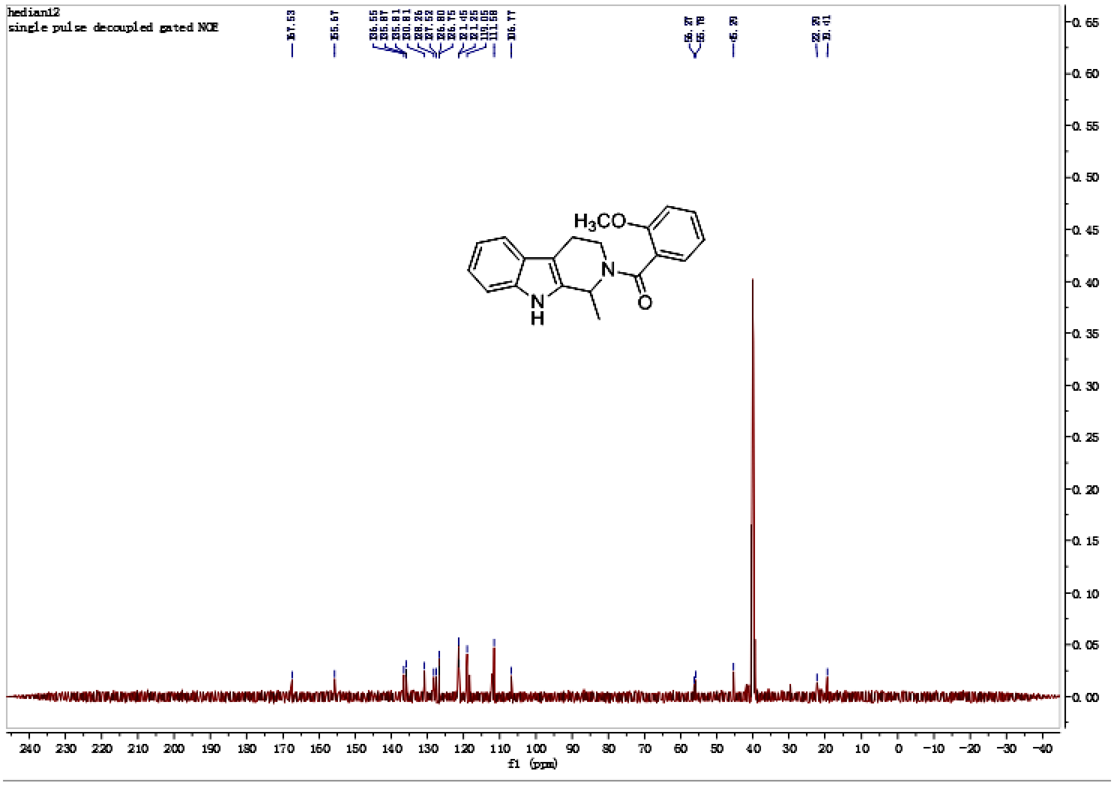

S44: 13C-NMR of 11c

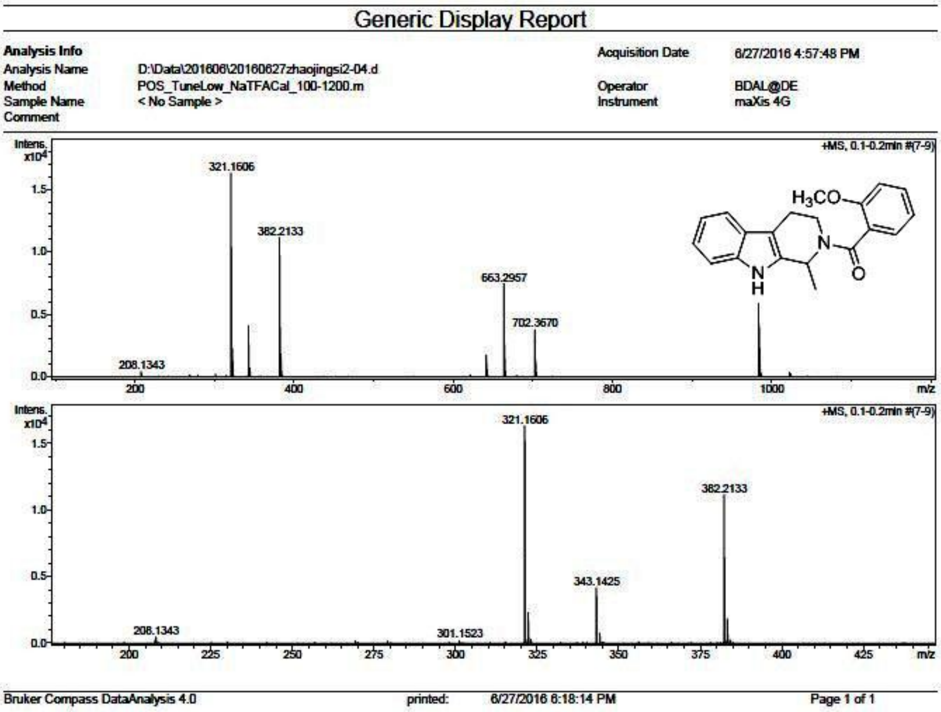

S45: ESI-MS of 11c

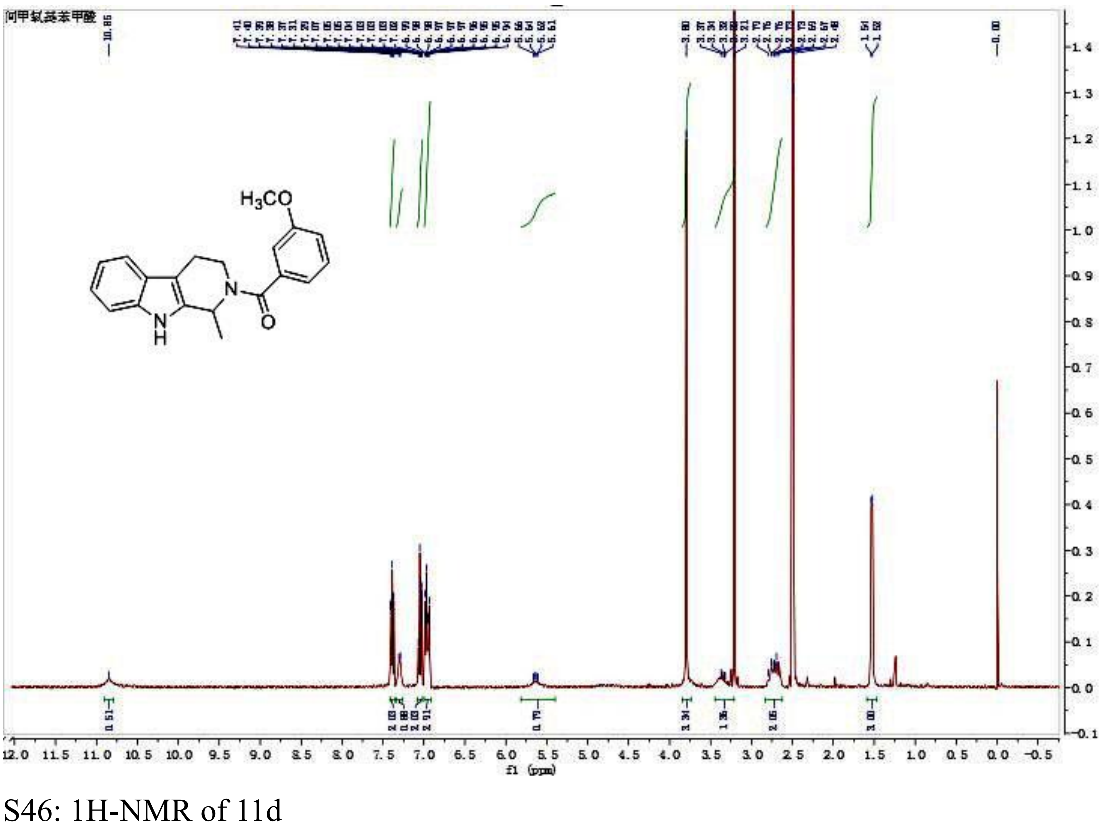

S46: 1H-NMR of 11d

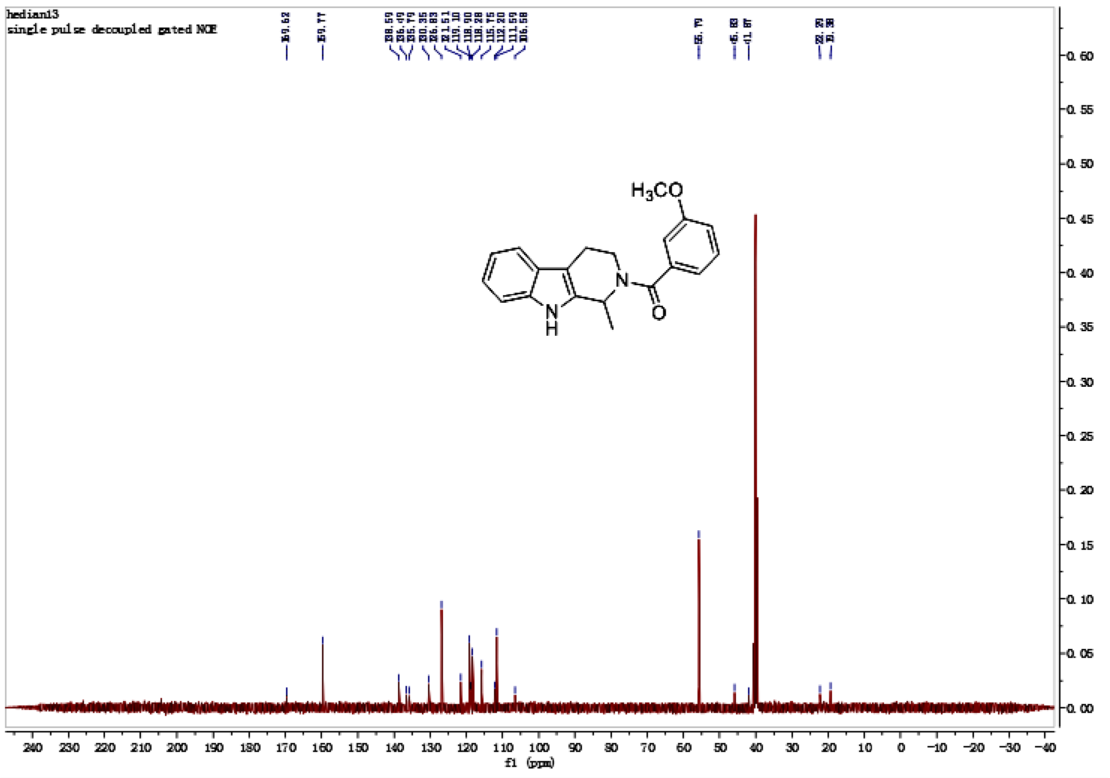

S47:13C-NMR of 11d

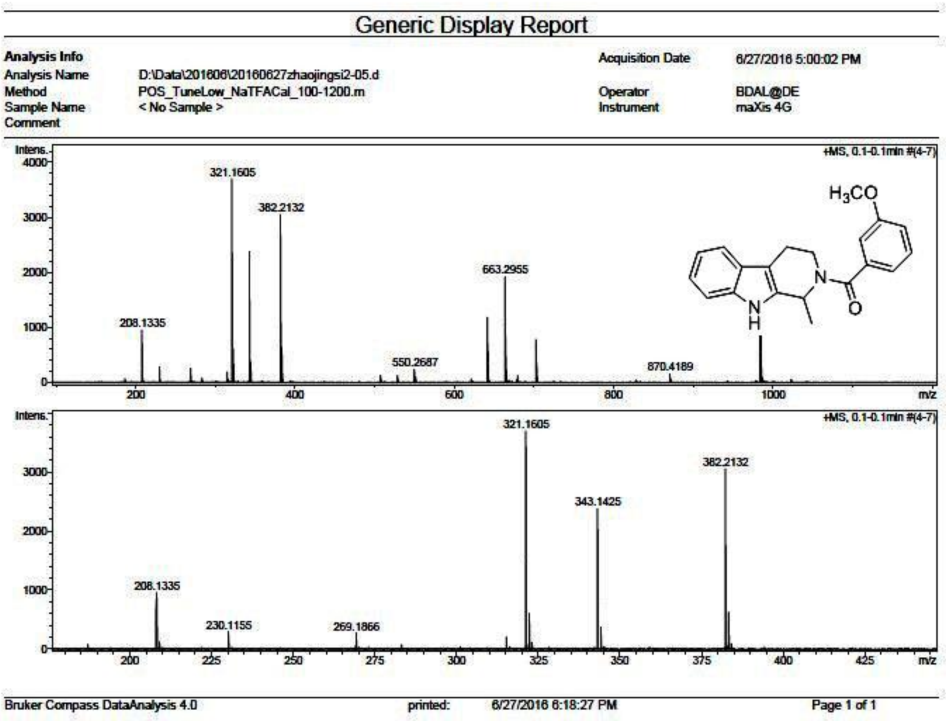

S48: ESI-MS of 11d

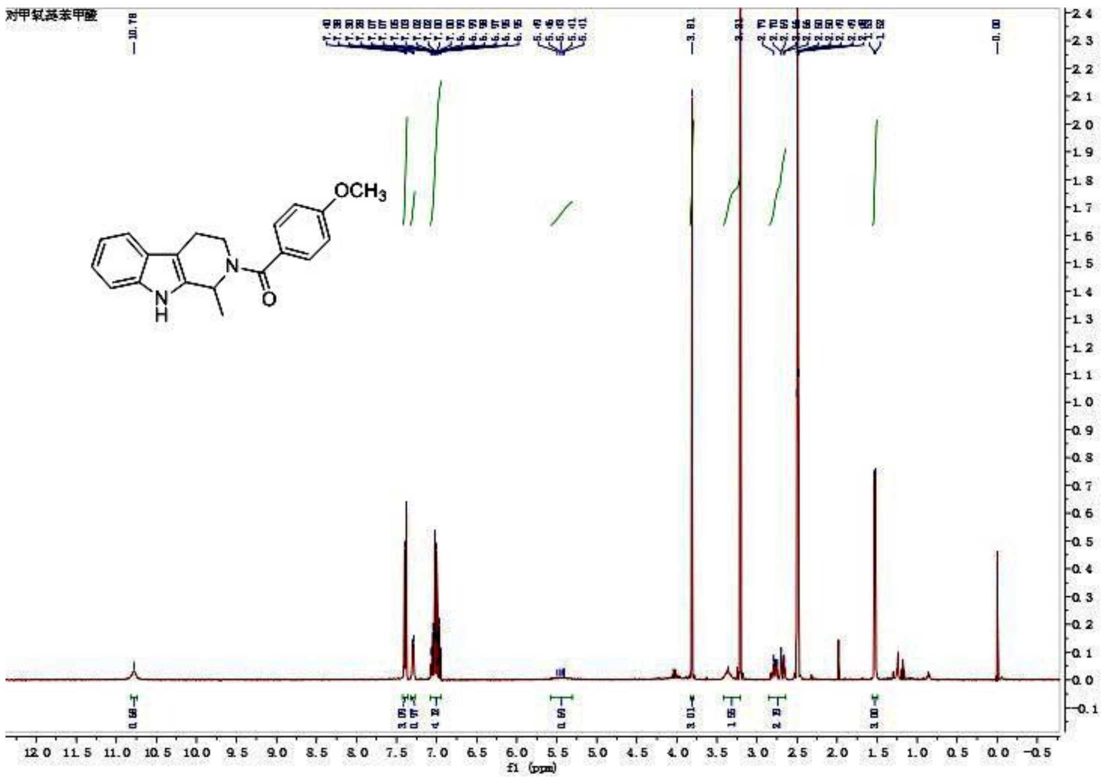

S49: 1H-NMR of 11e

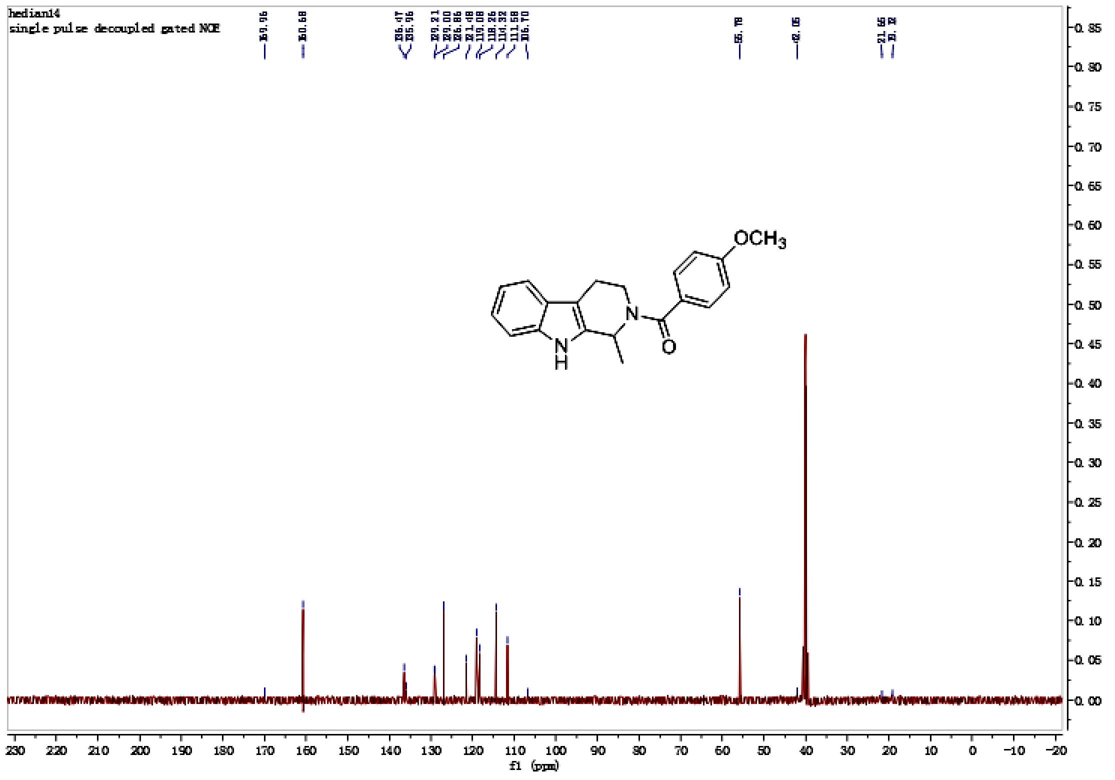

S50: 13C-NMR of 11e

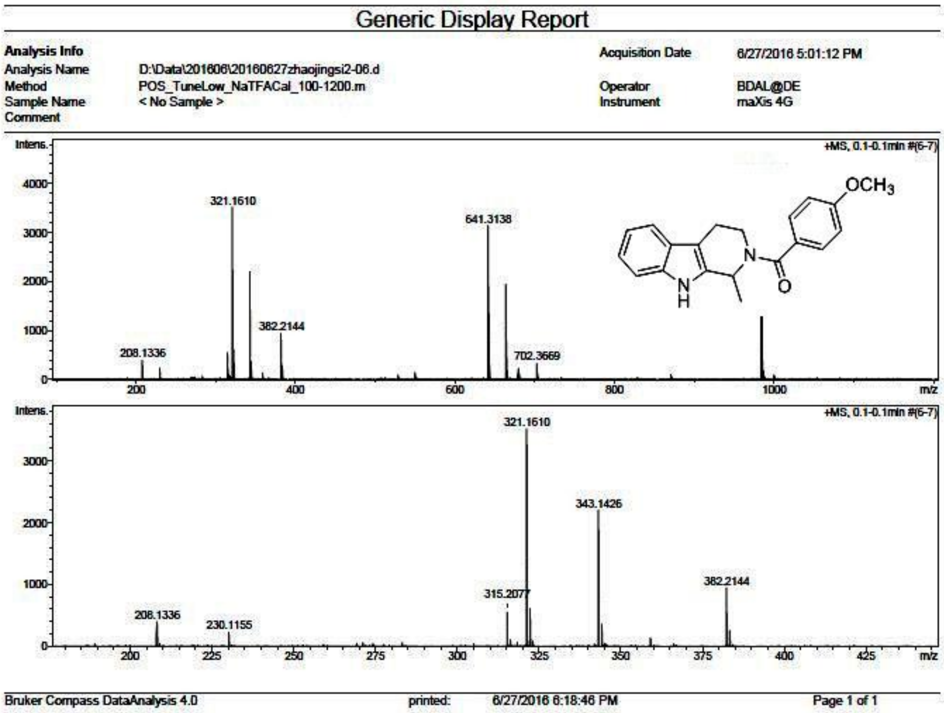

S51: ESI-MS of 11e

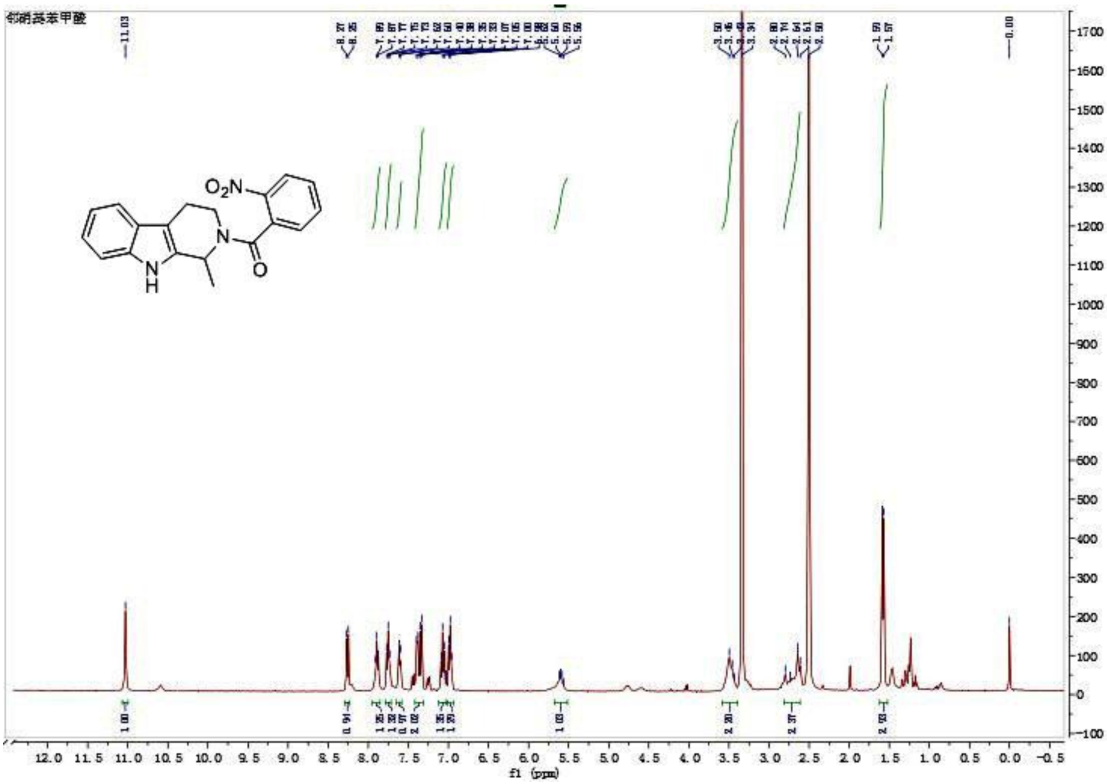

S52: 1H-NMR of 11f

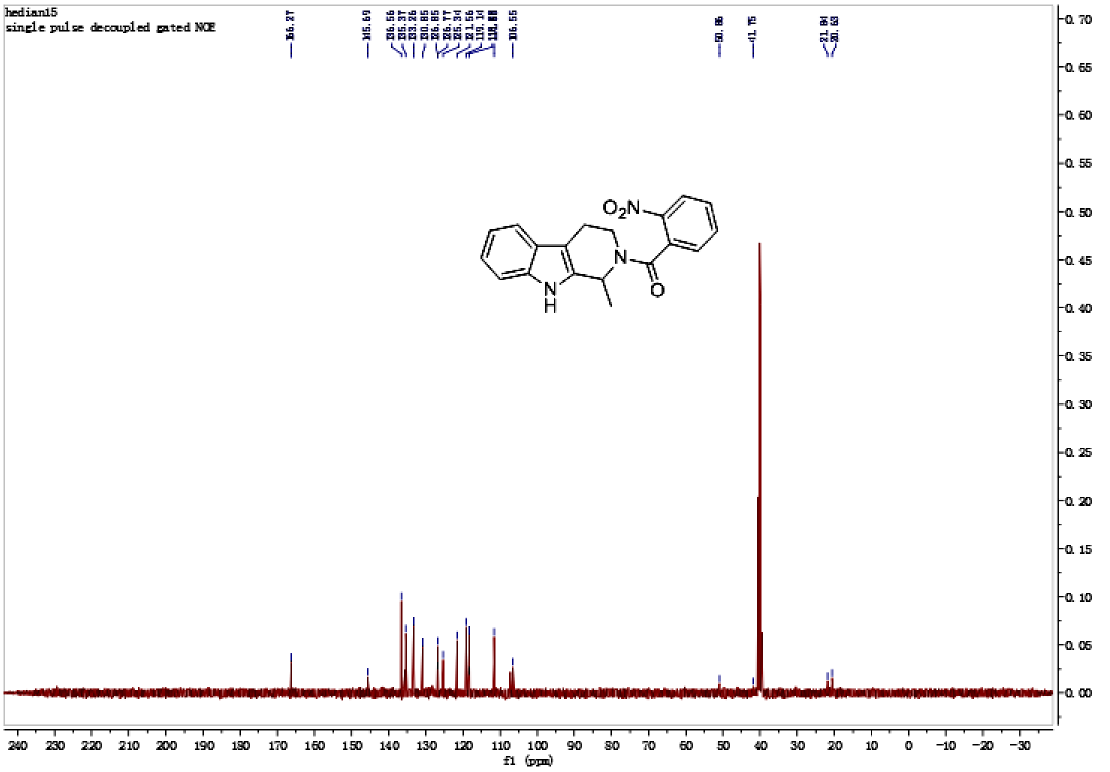

S53: 13C-NMR of 11f

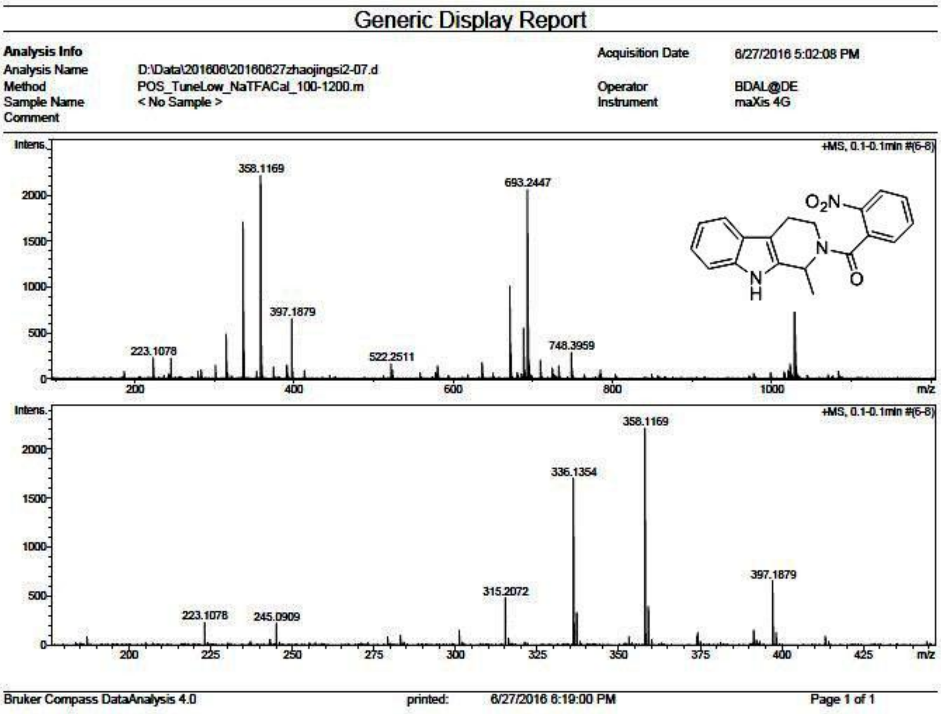

S54: ESI-MS of 11f

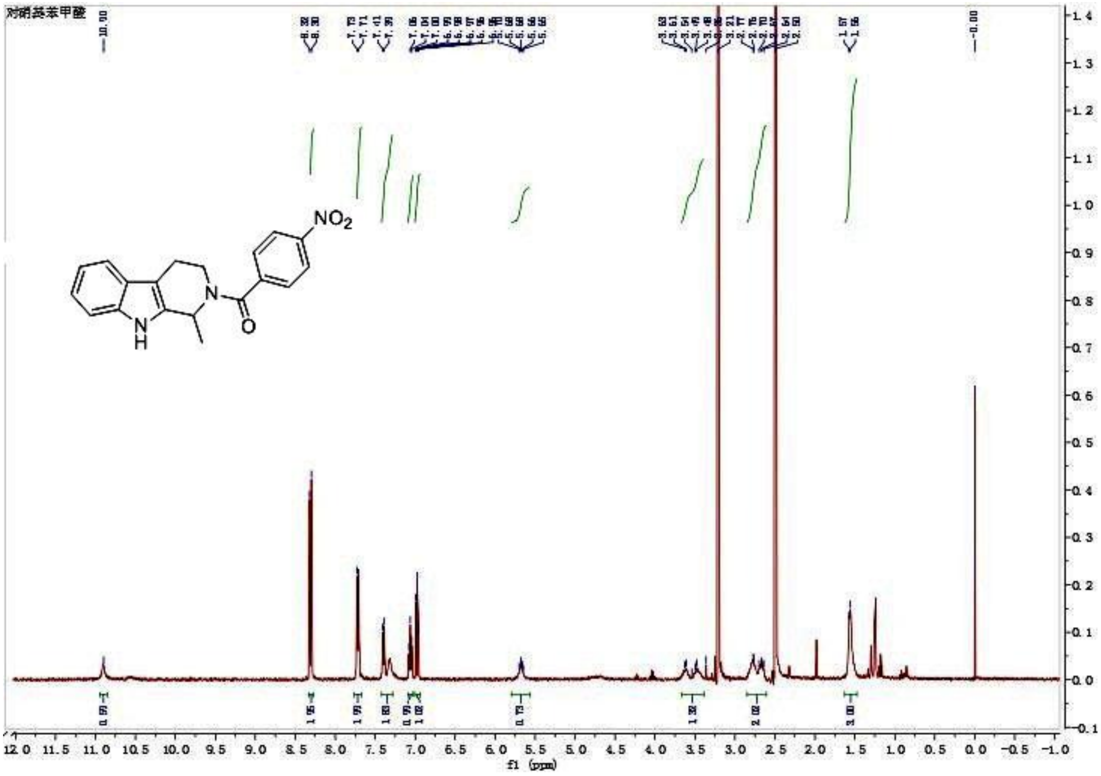

S55: 1H-NMR of 11g

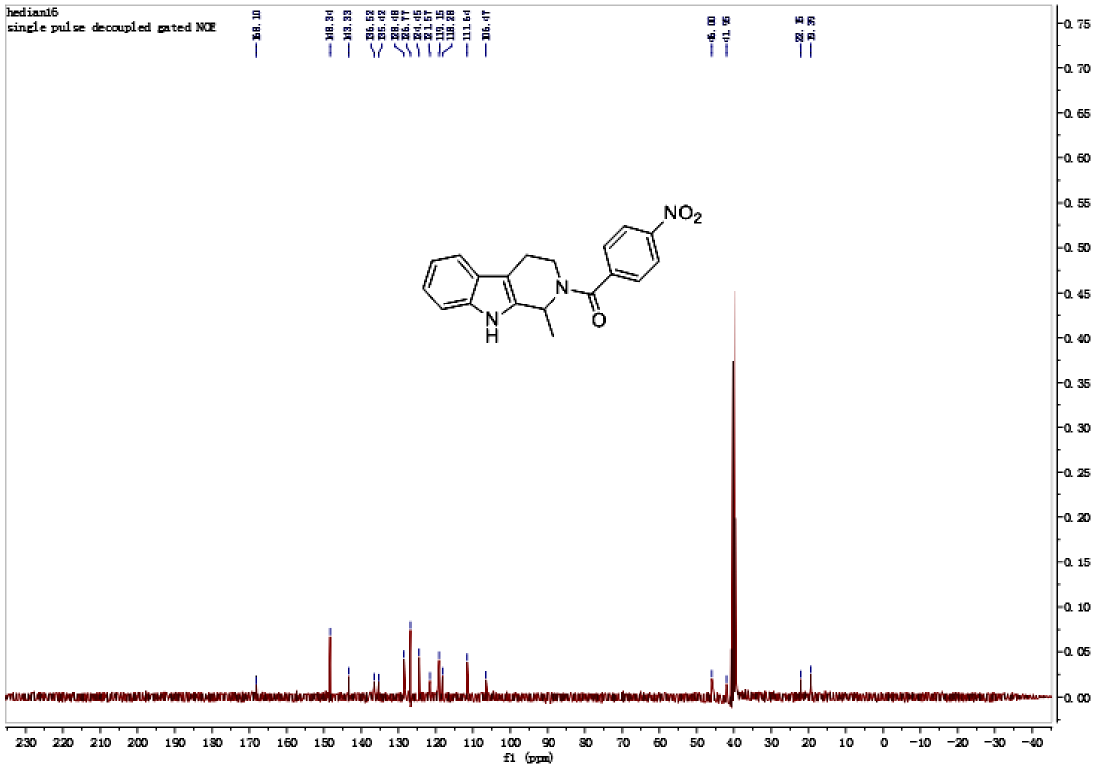

S56: 13C-NMR of 11g

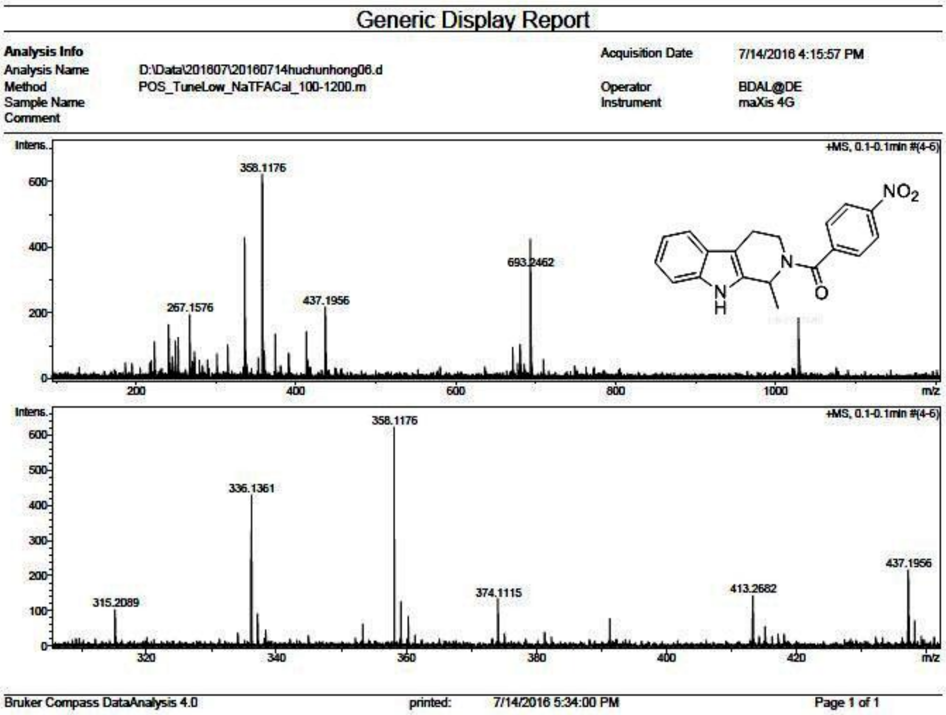

S57: ESI-MS of 11g

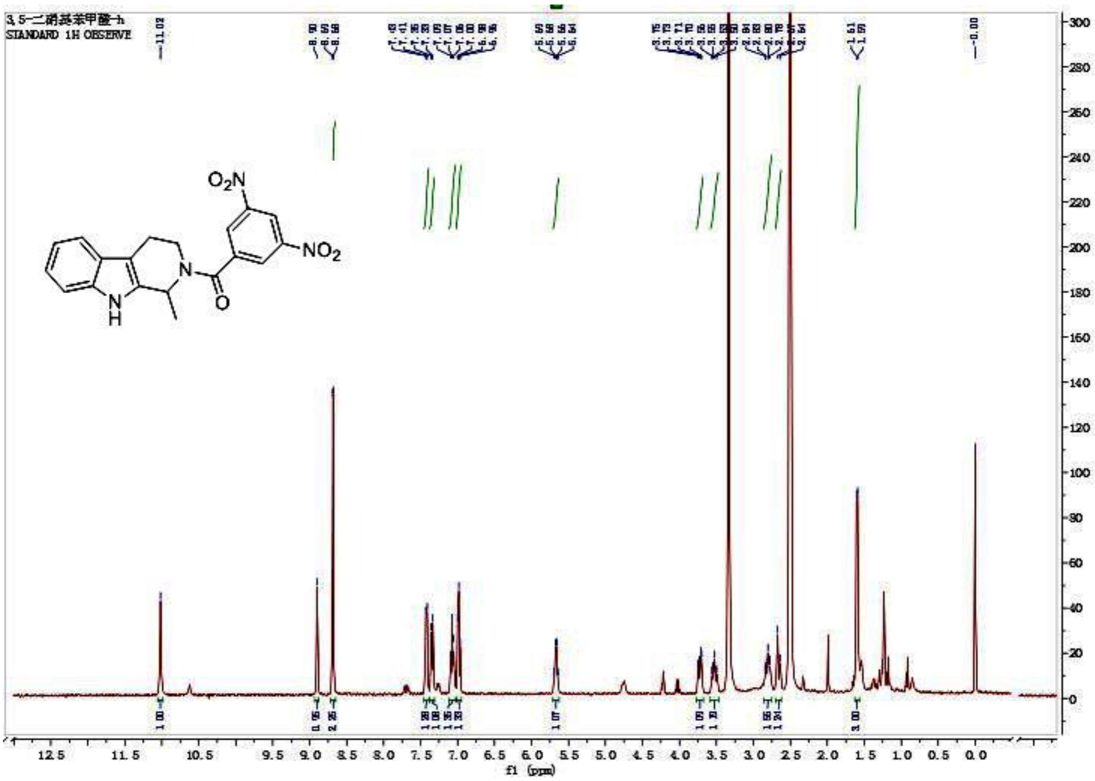

S58: 1H-NMR of 11h

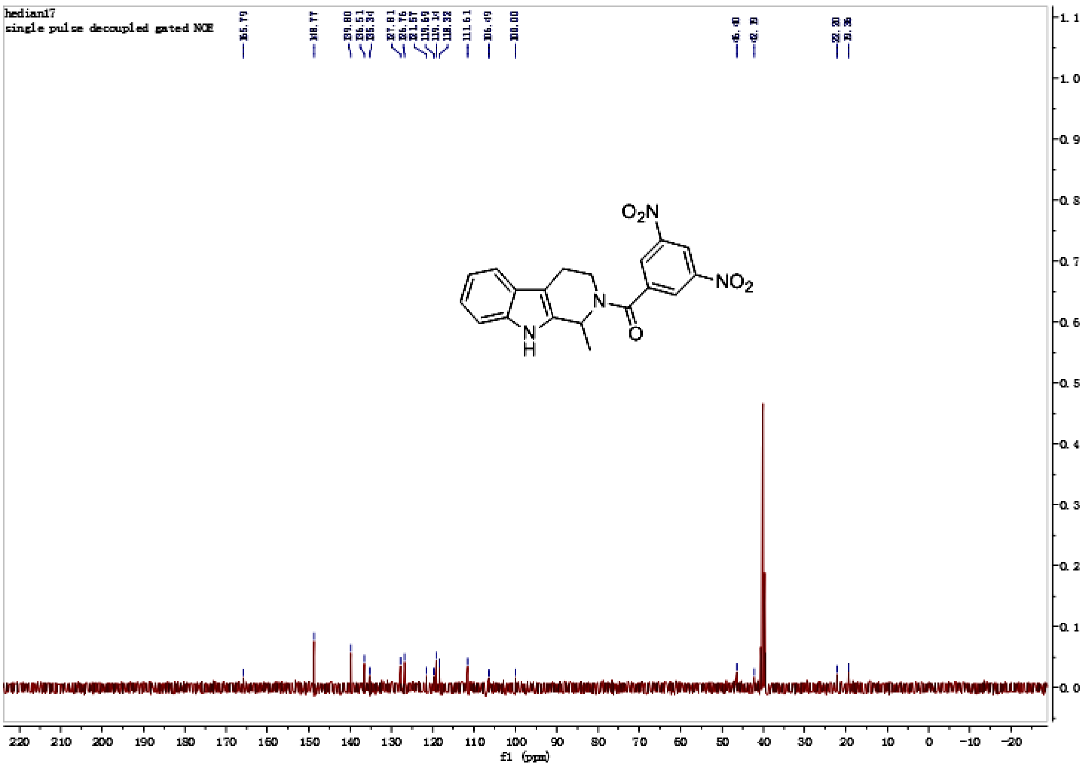

S59: 13C-NMR of 11h

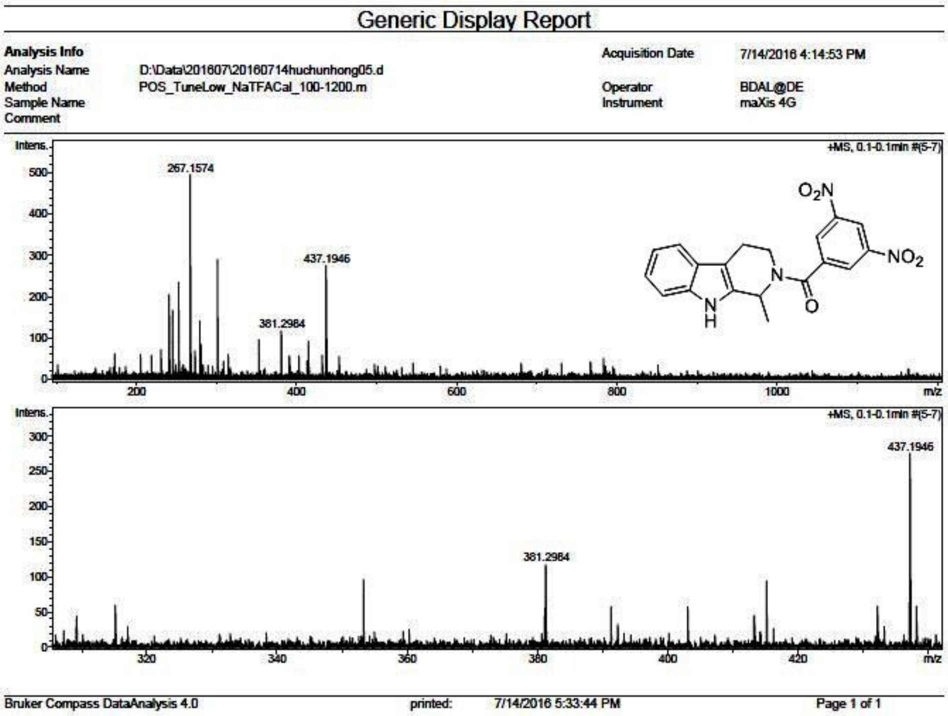

S60: ESI-MS of 11h

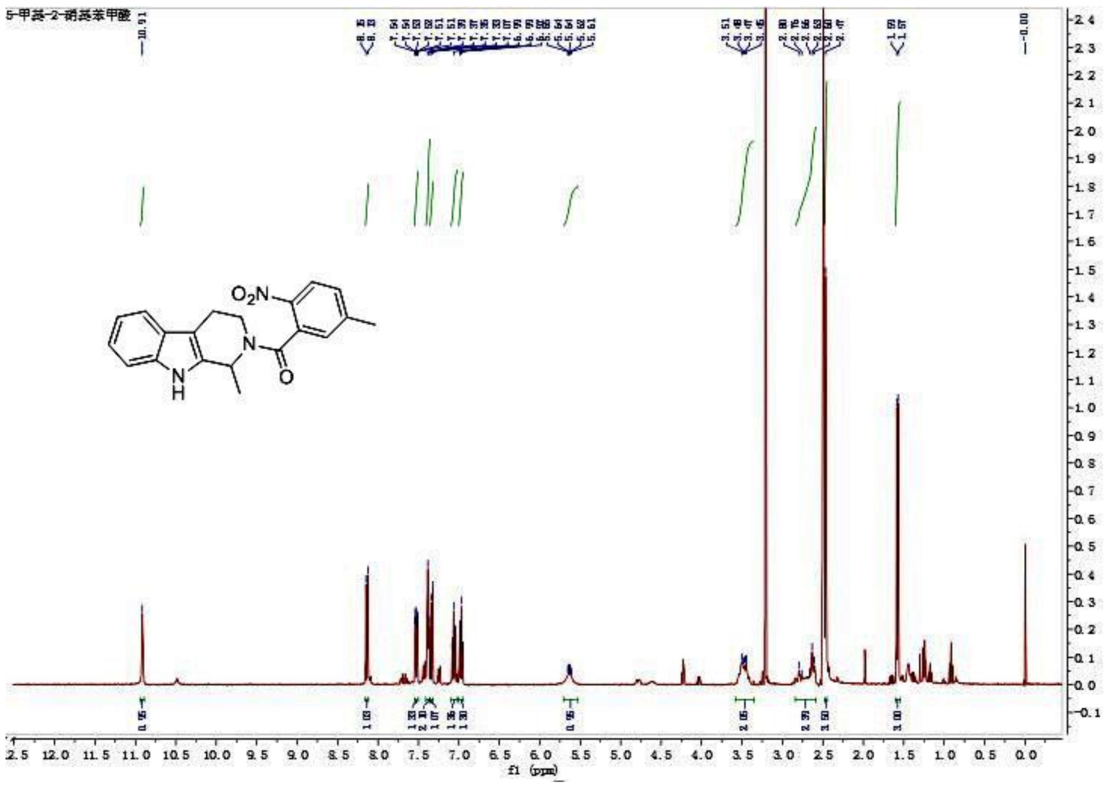

S61: 1H-NMR of 11i

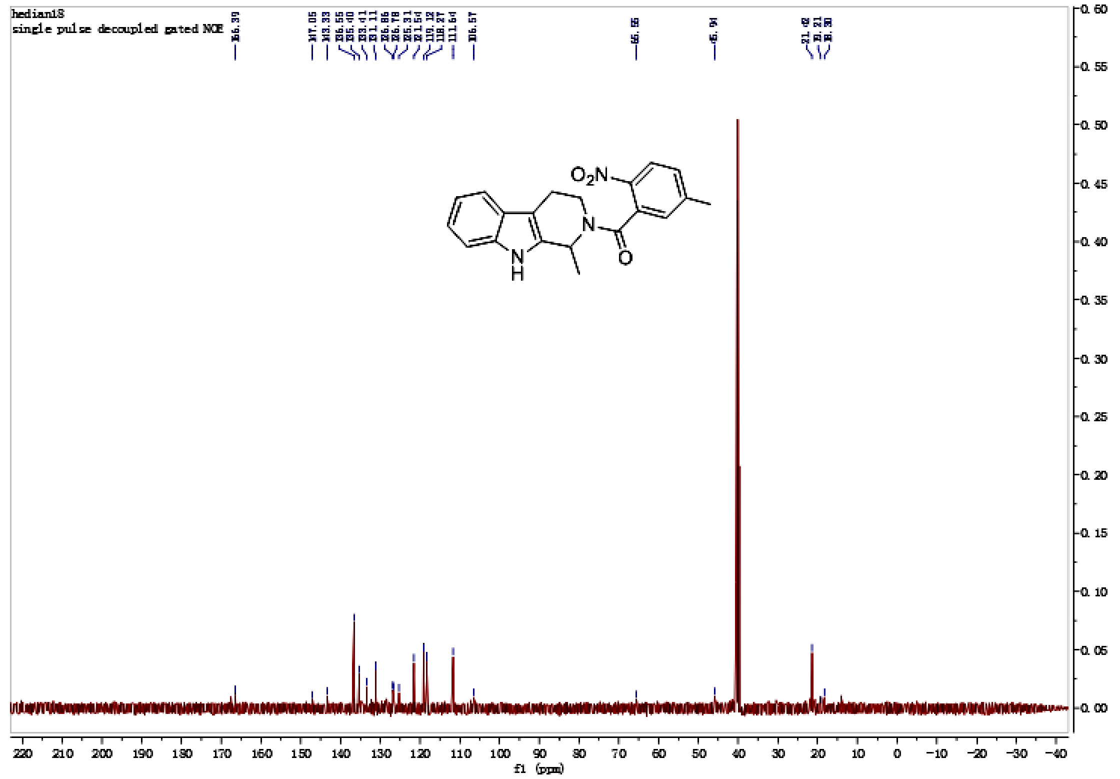

S62: 13C-NMR of 11i

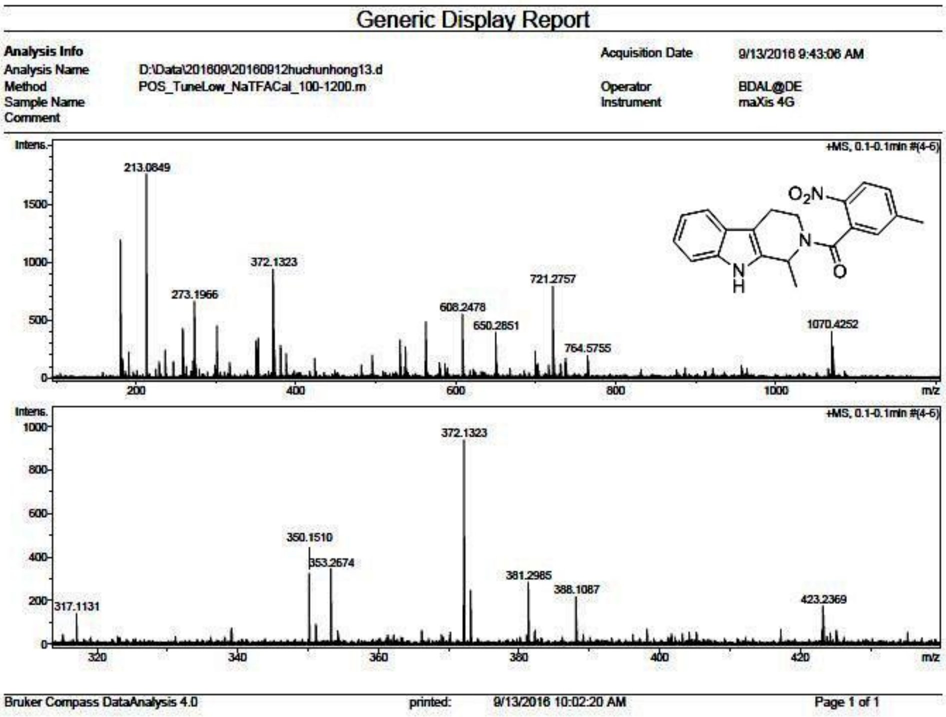

S63: ESI-MS of 11i

S 1. Crystal data and structure refinement for 7l.

Identification code	2_a	
Empirical formula	C28 H29 N3 O5	
Formula weight	487.54	
Temperature	296(2) K	
Wavelength	71.073 pm	
Crystal system	Monoclinic	
Space group	$P2_1/c$	
Unit cell dimensions	a = 870.50(11) pm	α= 90°.
	b = 2713.8(3) pm	β= 92.221(2)°.
	c = 1041.21(14) pm	γ = 90°.
Volume	2.4579(5) nm^3	
Z	4	
Density (calculated)	1.318 Mg/m^3	
Absorption coefficient	0.091 mm^{-1}	
F(000)	1032	
Crystal size	0.300 x 0.200 x 0.200 mm^3	
Theta range for data collection	1.501 to 31.345°.	
Index ranges	-12<=h<=10, -38<=k<=34, -13<=l<=13	
Reflections collected	16974	
Independent reflections	7068 [R(int) = 0.0447]	
Completeness to theta = 25.242°	99.7 %	
Absorption correction	Semi-empirical from equivalents	
Refinement method	Full-matrix least-squares on F^2	
Data / restraints / parameters	7068 / 0 / 327	
Goodness-of-fit on F^2	1.000	
Final R indices [I>2sigma(I)]	R1 = 0.0485, wR2 = 0.0893	
R indices (all data)	R1 = 0.1030, wR2 = 0.1067	
Extinction coefficient	n/a	
Largest diff. peak and hole	0.149 and -0.194 e.$Å^{-3}$	

S 2. Atomic coordinates (x 10^4) and equivalent isotropic displacement parameters (pm^2x 10^{-1}) for 7l. U(eq) is defined as one third of the trace of the orthogonalized U^{ij} tensor.

	x	y	z	U(eq)
C(1)	5206(2)	5910(1)	1929(2)	48(1)
C(2)	5924(2)	5620(1)	1024(2)	52(1)
C(3)	6884(2)	5118(1)	2657(2)	70(1)
C(4)	6188(2)	5374(1)	3603(2)	64(1)
C(5)	5327(2)	5784(1)	3243(2)	52(1)
C(6)	4453(2)	6136(1)	3933(2)	52(1)
C(7)	4108(2)	6197(1)	5217(2)	62(1)
C(8)	3180(2)	6580(1)	5561(2)	66(1)
C(9)	2591(2)	6907(1)	4634(2)	58(1)
C(10)	2897(2)	6858(1)	3362(2)	54(1)
C(11)	3834(2)	6466(1)	3018(2)	48(1)
C(12)	5794(2)	5695(1)	-405(2)	66(1)
C(13)	943(2)	7597(1)	4208(2)	82(1)
C(14)	3932(2)	6620(1)	662(1)	51(1)
C(15)	5196(2)	6976(1)	319(1)	52(1)
C(16)	5619(2)	7344(1)	1358(1)	49(1)
C(17)	6811(2)	7716(1)	967(1)	49(1)
C(18)	7297(2)	8048(1)	2077(1)	55(1)
C(19)	8472(2)	8420(1)	1735(1)	57(1)
C(20)	10088(2)	8991(1)	2856(1)	48(1)
C(21)	10599(2)	9185(1)	4116(1)	48(1)
C(22)	9987(2)	9054(1)	5202(1)	43(1)
C(23)	10449(2)	9239(1)	6482(1)	41(1)
C(24)	11302(2)	9670(1)	6616(1)	54(1)
C(25)	11695(2)	9862(1)	7804(2)	62(1)
C(26)	11223(2)	9636(1)	8900(2)	61(1)
C(27)	10375(2)	9213(1)	8817(1)	53(1)
C(28)	10016(2)	9018(1)	7620(1)	42(1)
N(1)	6770(2)	5232(1)	1402(2)	63(1)
N(2)	4281(1)	6329(1)	1809(1)	49(1)
N(3)	9189(2)	8548(1)	7616(1)	52(1)

O(1)	8974(1)	8660(1)	2925(1)	61(1)
O(2)	10617(2)	9117(1)	1863(1)	73(1)
O(3)	9416(2)	8255(1)	6752(1)	75(1)
O(4)	8358(2)	8460(1)	8494(1)	82(1)
O(5)	1692(1)	7276(1)	5111(1)	75(1)

S 3. Bond lengths [pm] and angles [°] for 7l.

C(1)-C(2)	139.5(2)
C(1)-N(2)	139.58(17)
C(1)-C(5)	141.0(2)
C(2)-N(1)	133.32(19)
C(2)-C(12)	150.2(2)
C(3)-N(1)	134.3(2)
C(3)-C(4)	136.6(2)
C(3)-H(3)	93.00
C(4)-C(5)	138.6(2)
C(4)-H(4)	93.00
C(5)-C(6)	143.1(2)
C(6)-C(7)	139.1(2)
C(6)-C(11)	140.0(2)
C(7)-C(8)	137.3(2)
C(7)-H(7)	93.00
C(8)-C(9)	139.5(2)
C(8)-H(8)	93.00
C(9)-C(10)	136.7(2)
C(9)-O(5)	137.40(18)
C(10)-C(11)	139.7(2)
C(10)-H(10)	93.00
C(11)-N(2)	138.25(18)
C(12)-H(12A)	96.00
C(12)-H(12B)	96.00
C(12)-H(12C)	96.00
C(13)-O(5)	142.1(2)
C(13)-H(13A)	96.00
C(13)-H(13B)	96.00
C(13)-H(13C)	96.00
C(14)-N(2)	145.31(18)
C(14)-C(15)	151.7(2)
C(14)-H(14A)	97.00
C(14)-H(14B)	97.00
C(15)-C(16)	150.81(19)

C(15)-H(15A)	97.00
C(15)-H(15B)	97.00
C(16)-C(17)	151.47(19)
C(16)-H(16A)	97.00
C(16)-H(16B)	97.00
C(17)-C(18)	151.33(19)
C(17)-H(17A)	97.00
C(17)-H(17B)	97.00
C(18)-C(19)	149.2(2)
C(18)-H(18A)	97.00
C(18)-H(18B)	97.00
C(19)-O(1)	145.17(16)
C(19)-H(19A)	97.00
C(19)-H(19B)	97.00
C(20)-O(2)	119.79(16)
C(20)-O(1)	132.53(16)
C(20)-C(21)	146.67(19)
C(21)-C(22)	131.61(19)
C(21)-H(21)	93.00
C(22)-C(23)	146.56(18)
C(22)-H(22)	93.00
C(23)-C(24)	138.91(19)
C(23)-C(28)	139.32(18)
C(24)-C(25)	137.5(2)
C(24)-H(24)	93.00
C(25)-C(26)	137.2(2)
C(25)-H(25)	93.00
C(26)-C(27)	136.7(2)
C(26)-H(26)	93.00
C(27)-C(28)	137.87(19)
C(27)-H(27)	93.00
C(28)-N(3)	146.30(18)
N(3)-O(4)	121.17(15)
N(3)-O(3)	122.37(16)
C(2)-C(1)-N(2)	132.21(14)

C(2)-C(1)-C(5)	119.99(14)
N(2)-C(1)-C(5)	107.77(13)
N(1)-C(2)-C(1)	120.22(15)
N(1)-C(2)-C(12)	114.79(14)
C(1)-C(2)-C(12)	124.96(14)
N(1)-C(3)-C(4)	124.47(16)
N(1)-C(3)-H(3)	117.8
C(4)-C(3)-H(3)	117.8
C(3)-C(4)-C(5)	117.64(17)
C(3)-C(4)-H(4)	121.2
C(5)-C(4)-H(4)	121.2
C(4)-C(5)-C(1)	118.43(15)
C(4)-C(5)-C(6)	133.79(16)
C(1)-C(5)-C(6)	107.78(13)
C(7)-C(6)-C(11)	119.11(15)
C(7)-C(6)-C(5)	134.50(16)
C(11)-C(6)-C(5)	106.37(14)
C(8)-C(7)-C(6)	119.47(16)
C(8)-C(7)-H(7)	120.3
C(6)-C(7)-H(7)	120.3
C(7)-C(8)-C(9)	120.42(17)
C(7)-C(8)-H(8)	119.8
C(9)-C(8)-H(8)	119.8
C(10)-C(9)-O(5)	123.74(16)
C(10)-C(9)-C(8)	121.85(16)
O(5)-C(9)-C(8)	114.40(16)
C(9)-C(10)-C(11)	117.43(15)
C(9)-C(10)-H(10)	121.3
C(11)-C(10)-H(10)	121.3
N(2)-C(11)-C(10)	128.76(14)
N(2)-C(11)-C(6)	109.52(13)
C(10)-C(11)-C(6)	121.71(15)
C(2)-C(12)-H(12A)	109.5
C(2)-C(12)-H(12B)	109.5
H(12A)-C(12)-H(12B)	109.5
C(2)-C(12)-H(12C)	109.5

H(12A)-C(12)-H(12C)	109.5
H(12B)-C(12)-H(12C)	109.5
O(5)-C(13)-H(13A)	109.5
O(5)-C(13)-H(13B)	109.5
H(13A)-C(13)-H(13B)	109.5
O(5)-C(13)-H(13C)	109.5
H(13A)-C(13)-H(13C)	109.5
H(13B)-C(13)-H(13C)	109.5
N(2)-C(14)-C(15)	114.16(12)
N(2)-C(14)-H(14A)	108.7
C(15)-C(14)-H(14A)	108.7
N(2)-C(14)-H(14B)	108.7
C(15)-C(14)-H(14B)	108.7
H(14A)-C(14)-H(14B)	107.6
C(16)-C(15)-C(14)	114.30(13)
C(16)-C(15)-H(15A)	108.7
C(14)-C(15)-H(15A)	108.7
C(16)-C(15)-H(15B)	108.7
C(14)-C(15)-H(15B)	108.7
H(15A)-C(15)-H(15B)	107.6
C(15)-C(16)-C(17)	113.52(12)
C(15)-C(16)-H(16A)	108.9
C(17)-C(16)-H(16A)	108.9
C(15)-C(16)-H(16B)	108.9
C(17)-C(16)-H(16B)	108.9
H(16A)-C(16)-H(16B)	107.7
C(18)-C(17)-C(16)	111.43(12)
C(18)-C(17)-H(17A)	109.3
C(16)-C(17)-H(17A)	109.3
C(18)-C(17)-H(17B)	109.3
C(16)-C(17)-H(17B)	109.3
H(17A)-C(17)-H(17B)	108.0
C(19)-C(18)-C(17)	113.29(12)
C(19)-C(18)-H(18A)	108.9
C(17)-C(18)-H(18A)	108.9
C(19)-C(18)-H(18B)	108.9

C(17)-C(18)-H(18B)	108.9
H(18A)-C(18)-H(18B)	107.7
O(1)-C(19)-C(18)	106.66(12)
O(1)-C(19)-H(19A)	110.4
C(18)-C(19)-H(19A)	110.4
O(1)-C(19)-H(19B)	110.4
C(18)-C(19)-H(19B)	110.4
H(19A)-C(19)-H(19B)	108.6
O(2)-C(20)-O(1)	123.15(13)
O(2)-C(20)-C(21)	123.79(14)
O(1)-C(20)-C(21)	113.06(12)
C(22)-C(21)-C(20)	123.55(14)
C(22)-C(21)-H(21)	118.2
C(20)-C(21)-H(21)	118.2
C(21)-C(22)-C(23)	125.66(14)
C(21)-C(22)-H(22)	117.2
C(23)-C(22)-H(22)	117.2
C(24)-C(23)-C(28)	116.00(12)
C(24)-C(23)-C(22)	120.36(13)
C(28)-C(23)-C(22)	123.56(13)
C(25)-C(24)-C(23)	121.60(15)
C(25)-C(24)-H(24)	119.2
C(23)-C(24)-H(24)	119.2
C(26)-C(25)-C(24)	120.39(16)
C(26)-C(25)-H(25)	119.8
C(24)-C(25)-H(25)	119.8
C(27)-C(26)-C(25)	120.16(14)
C(27)-C(26)-H(26)	119.9
C(25)-C(26)-H(26)	119.9
C(26)-C(27)-C(28)	118.84(14)
C(26)-C(27)-H(27)	120.6
C(28)-C(27)-H(27)	120.6
C(27)-C(28)-C(23)	122.99(14)
C(27)-C(28)-N(3)	115.52(13)
C(23)-C(28)-N(3)	121.46(12)
C(2)-N(1)-C(3)	119.20(15)

C(11)-N(2)-C(1)	108.56(12)
C(11)-N(2)-C(14)	123.03(12)
C(1)-N(2)-C(14)	128.12(13)
O(4)-N(3)-O(3)	122.89(14)
O(4)-N(3)-C(28)	118.60(13)
O(3)-N(3)-C(28)	118.44(13)
C(20)-O(1)-C(19)	117.00(11)
C(9)-O(5)-C(13)	117.42(15)

Symmetry transformations used to generate equivalent atoms:

S 4. Anisotropic displacement parameters ($pm^2 x 10^{-1}$) for 7l. The anisotropic displacement factor exponent takes the form: $-2\pi^2[h^2 a^{*2}U^{11} + ... + 2 h k a^* b^* U^{12}]$

	U^{11}	U^{22}	U^{33}	U^{23}	U^{13}	U^{12}
C(1)	39(1)	38(1)	66(1)	-2(1)	3(1)	-5(1)
C(2)	44(1)	44(1)	68(1)	-9(1)	2(1)	-6(1)
C(3)	67(1)	54(1)	87(1)	-2(1)	-6(1)	12(1)
C(4)	62(1)	57(1)	72(1)	6(1)	-4(1)	4(1)
C(5)	44(1)	45(1)	66(1)	2(1)	2(1)	-5(1)
C(6)	47(1)	48(1)	61(1)	0(1)	6(1)	-7(1)
C(7)	63(1)	59(1)	66(1)	3(1)	6(1)	-7(1)
C(8)	68(1)	68(1)	64(1)	-10(1)	19(1)	-15(1)
C(9)	50(1)	52(1)	72(1)	-12(1)	17(1)	-7(1)
C(10)	46(1)	47(1)	70(1)	-5(1)	10(1)	-2(1)
C(11)	41(1)	43(1)	60(1)	-3(1)	9(1)	-7(1)
C(12)	71(1)	58(1)	72(1)	-14(1)	7(1)	5(1)
C(13)	66(1)	72(1)	108(2)	-29(1)	0(1)	14(1)
C(14)	49(1)	47(1)	57(1)	-4(1)	-4(1)	0(1)
C(15)	60(1)	47(1)	49(1)	0(1)	3(1)	-1(1)
C(16)	54(1)	46(1)	48(1)	2(1)	4(1)	-4(1)
C(17)	53(1)	48(1)	46(1)	0(1)	7(1)	-6(1)
C(18)	59(1)	62(1)	43(1)	1(1)	5(1)	-14(1)
C(19)	68(1)	67(1)	37(1)	-7(1)	4(1)	-20(1)
C(20)	56(1)	48(1)	39(1)	-2(1)	8(1)	-5(1)
C(21)	53(1)	52(1)	39(1)	-3(1)	3(1)	-9(1)
C(22)	50(1)	42(1)	38(1)	1(1)	-2(1)	-3(1)
C(23)	44(1)	43(1)	36(1)	-1(1)	-2(1)	4(1)
C(24)	66(1)	50(1)	44(1)	0(1)	-1(1)	-8(1)
C(25)	74(1)	55(1)	58(1)	-10(1)	-6(1)	-9(1)
C(26)	70(1)	69(1)	44(1)	-16(1)	-9(1)	2(1)
C(27)	59(1)	65(1)	35(1)	-2(1)	1(1)	6(1)
C(28)	44(1)	46(1)	36(1)	0(1)	-1(1)	5(1)
N(1)	55(1)	50(1)	85(1)	-9(1)	-1(1)	6(1)
N(2)	45(1)	41(1)	60(1)	-1(1)	6(1)	-1(1)
N(3)	62(1)	55(1)	40(1)	7(1)	0(1)	-4(1)

O(1)	72(1)	75(1)	35(1)	-6(1)	6(1)	-30(1)
O(2)	100(1)	82(1)	39(1)	-8(1)	20(1)	-37(1)
O(3)	116(1)	56(1)	53(1)	-5(1)	8(1)	-17(1)
O(4)	93(1)	93(1)	63(1)	9(1)	26(1)	-23(1)
O(5)	69(1)	70(1)	88(1)	-21(1)	24(1)	3(1)

S 5. Hydrogen coordinates (x 10^4) and isotropic displacement parameters (pm^{2}x 10 $^{-1}$) for 7l.

	x	y	z	U(eq)
H(3)	7479	4847	2899	84
H(4)	6287	5276	4458	77
H(7)	4504	5980	5837	75
H(8)	2941	6621	6418	80
H(10)	2496	7078	2751	65
H(12A)	6452	5466	-819	100
H(12B)	6098	6026	-606	100
H(12C)	4749	5643	-702	100
H(13A)	1697	7759	3710	124
H(13B)	362	7838	4657	124
H(13C)	263	7409	3648	124
H(14A)	2998	6805	788	61
H(14B)	3736	6398	-56	61
H(15A)	6105	6788	123	63
H(15B)	4870	7153	-453	63
H(16A)	6011	7167	2113	59
H(16B)	4699	7518	1593	59
H(17A)	7703	7543	665	59
H(17B)	6389	7915	264	59
H(18A)	6398	8217	2378	66
H(18B)	7710	7846	2779	66
H(19A)	9334	8261	1341	69
H(19B)	8030	8659	1135	69
H(21)	11400	9412	4151	58
H(22)	9192	8826	5151	52
H(24)	11615	9832	5883	64
H(25)	12285	10148	7866	75
H(26)	11481	9771	9700	74
H(27)	10047	9059	9556	64

S 6. Torsion angles [°] for 7l.

N(2)-C(1)-C(2)-N(1)	179.68(14)
C(5)-C(1)-C(2)-N(1)	-2.4(2)
N(2)-C(1)-C(2)-C(12)	-2.3(3)
C(5)-C(1)-C(2)-C(12)	175.70(14)
N(1)-C(3)-C(4)-C(5)	-1.2(3)
C(3)-C(4)-C(5)-C(1)	0.7(2)
C(3)-C(4)-C(5)-C(6)	179.98(17)
C(2)-C(1)-C(5)-C(4)	1.0(2)
N(2)-C(1)-C(5)-C(4)	179.45(13)
C(2)-C(1)-C(5)-C(6)	-178.44(13)
N(2)-C(1)-C(5)-C(6)	-0.02(16)
C(4)-C(5)-C(6)-C(7)	-1.3(3)
C(1)-C(5)-C(6)-C(7)	178.03(17)
C(4)-C(5)-C(6)-C(11)	-179.62(17)
C(1)-C(5)-C(6)-C(11)	-0.27(16)
C(11)-C(6)-C(7)-C(8)	-0.4(2)
C(5)-C(6)-C(7)-C(8)	-178.54(16)
C(6)-C(7)-C(8)-C(9)	-0.4(2)
C(7)-C(8)-C(9)-C(10)	0.7(2)
C(7)-C(8)-C(9)-O(5)	-179.40(14)
O(5)-C(9)-C(10)-C(11)	179.86(13)
C(8)-C(9)-C(10)-C(11)	-0.3(2)
C(9)-C(10)-C(11)-N(2)	178.29(14)
C(9)-C(10)-C(11)-C(6)	-0.5(2)
C(7)-C(6)-C(11)-N(2)	-178.14(13)
C(5)-C(6)-C(11)-N(2)	0.46(16)
C(7)-C(6)-C(11)-C(10)	0.9(2)
C(5)-C(6)-C(11)-C(10)	179.48(13)
N(2)-C(14)-C(15)-C(16)	-58.41(17)
C(14)-C(15)-C(16)-C(17)	-176.38(12)
C(15)-C(16)-C(17)-C(18)	-175.03(13)
C(16)-C(17)-C(18)-C(19)	179.96(13)
C(17)-C(18)-C(19)-O(1)	-172.15(13)
O(2)-C(20)-C(21)-C(22)	178.14(16)

O(1)-C(20)-C(21)-C(22)	-1.8(2)
C(20)-C(21)-C(22)-C(23)	-179.64(13)
C(21)-C(22)-C(23)-C(24)	17.8(2)
C(21)-C(22)-C(23)-C(28)	-165.49(14)
C(28)-C(23)-C(24)-C(25)	0.3(2)
C(22)-C(23)-C(24)-C(25)	177.18(14)
C(23)-C(24)-C(25)-C(26)	-1.3(3)
C(24)-C(25)-C(26)-C(27)	0.9(3)
C(25)-C(26)-C(27)-C(28)	0.4(2)
C(26)-C(27)-C(28)-C(23)	-1.5(2)
C(26)-C(27)-C(28)-N(3)	176.36(13)
C(24)-C(23)-C(28)-C(27)	1.1(2)
C(22)-C(23)-C(28)-C(27)	-175.68(13)
C(24)-C(23)-C(28)-N(3)	-176.58(13)
C(22)-C(23)-C(28)-N(3)	6.6(2)
C(1)-C(2)-N(1)-C(3)	1.9(2)
C(12)-C(2)-N(1)-C(3)	-176.37(15)
C(4)-C(3)-N(1)-C(2)	-0.1(3)
C(10)-C(11)-N(2)-C(1)	-179.42(14)
C(6)-C(11)-N(2)-C(1)	-0.49(15)
C(10)-C(11)-N(2)-C(14)	6.3(2)
C(6)-C(11)-N(2)-C(14)	-174.80(12)
C(2)-C(1)-N(2)-C(11)	178.46(15)
C(5)-C(1)-N(2)-C(11)	0.31(15)
C(2)-C(1)-N(2)-C(14)	-7.6(2)
C(5)-C(1)-N(2)-C(14)	174.25(13)
C(15)-C(14)-N(2)-C(11)	93.98(16)
C(15)-C(14)-N(2)-C(1)	-79.17(17)
C(27)-C(28)-N(3)-O(4)	29.30(19)
C(23)-C(28)-N(3)-O(4)	-152.84(14)
C(27)-C(28)-N(3)-O(3)	-147.84(14)
C(23)-C(28)-N(3)-O(3)	30.0(2)
O(2)-C(20)-O(1)-C(19)	4.6(2)
C(21)-C(20)-O(1)-C(19)	-175.49(13)
C(18)-C(19)-O(1)-C(20)	176.56(13)
C(10)-C(9)-O(5)-C(13)	5.4(2)

C(8)-C(9)-O(5)-C(13)	-174.50(15)

Symmetry transformations used to generate equivalent atoms:

S 7. Crystal data and structure refinement for 7h.

Identification code	1_a	
Empirical formula	C30 H34 N2 O6	
Formula weight	518.59	
Temperature	296(2) K	
Wavelength	0.71073 Å	
Crystal system	Triclinic	
Space group	P-1	
Unit cell dimensions	a = 7.9502(12) Å	α= 81.041(3)°.
	b = 12.4483(19) Å	β= 80.934(3)°.
	c = 14.631(2) Å	γ = 73.883(3)°.
Volume	1364.1(4) $Å^3$	
Z	2	
Density (calculated)	1.263 Mg/m^3	
Absorption coefficient	0.088 mm^{-1}	
F(000)	552	
Crystal size	0.300 x 0.200 x 0.200 mm^3	
Theta range for data collection	1.419 to 31.405°.	
Index ranges	-11<=h<=4, -18<=k<=16, -19<=l<=19	
Reflections collected	9590	
Independent reflections	7171 [R(int) = 0.0380]	
Completeness to theta = 25.242°	99.3 %	
Absorption correction	Semi-empirical from equivalents	
Refinement method	Full-matrix least-squares on F^2	
Data / restraints / parameters	7171 / 0 / 349	
Goodness-of-fit on F^2	0.999	
Final R indices [I>2sigma(I)]	R1 = 0.0546, wR2 = 0.1246	
R indices (all data)	R1 = 0.1229, wR2 = 0.1606	
Extinction coefficient	0.009(2)	
Largest diff. peak and hole	0.187 and -0.174 e.$Å^{-3}$	

S 8. Atomic coordinates (x 10^4) and equivalent isotropic displacement parameters (Å^{2}x 10^3) for 7h. U(eq) is defined as one third of the trace of the orthogonalized U^{ij} tensor.

	x	y	z	U(eq)
C(1)	7165(3)	436(2)	2311(2)	63(1)
C(2)	6653(3)	-312(2)	1863(2)	63(1)
C(3)	5080(3)	-594(2)	2185(1)	56(1)
C(4)	4036(3)	-173(2)	2963(1)	52(1)
C(5)	4575(3)	549(2)	3432(1)	51(1)
C(6)	6120(3)	858(2)	3089(1)	59(1)
C(7)	9217(4)	1503(3)	2329(2)	103(1)
C(8)	7716(4)	-261(2)	268(2)	96(1)
C(9)	3013(3)	-1560(2)	1937(2)	73(1)
C(10)	3558(3)	985(2)	4277(1)	52(1)
C(11)	2129(3)	731(2)	4735(1)	56(1)
C(12)	1144(3)	1209(2)	5577(1)	53(1)
C(13)	858(2)	2465(2)	6670(1)	51(1)
C(14)	1793(2)	3259(2)	6872(1)	51(1)
C(15)	835(2)	3857(2)	7706(1)	51(1)
C(16)	1570(2)	4814(2)	7845(1)	54(1)
C(17)	3274(2)	4392(2)	8288(1)	52(1)
C(18)	4481(2)	5960(2)	8684(1)	51(1)
C(19)	3566(3)	6268(2)	9542(1)	63(1)
C(20)	5295(4)	7466(2)	9553(2)	83(1)
C(21)	6205(3)	7265(2)	8697(2)	71(1)
C(22)	5796(3)	6497(2)	8242(1)	55(1)
C(23)	6436(2)	6048(2)	7370(1)	53(1)
C(24)	7701(3)	6243(2)	6628(2)	66(1)
C(25)	8022(3)	5637(2)	5887(2)	68(1)
C(26)	7128(3)	4830(2)	5870(1)	64(1)
C(27)	5864(3)	4616(2)	6579(1)	57(1)
C(28)	5535(2)	5246(2)	7328(1)	50(1)
C(29)	6823(4)	3404(2)	5041(2)	91(1)
C(30)	2031(3)	5832(2)	10042(2)	86(1)
N(1)	4351(2)	5192(1)	8125(1)	51(1)

N(2)	4007(3)	6999(2)	9964(1)	77(1)
O(1)	8709(2)	682(2)	1935(1)	87(1)
O(2)	7782(2)	-846(1)	1163(1)	87(1)
O(3)	4678(2)	-1312(1)	1684(1)	71(1)
O(4)	-168(2)	964(1)	5982(1)	74(1)
O(5)	1819(2)	1945(1)	5856(1)	56(1)
O(6)	7577(2)	4283(2)	5088(1)	86(1)

S 9. Bond lengths [Å] and angles [°] for 7h.

C(1)-O(1)	1.359(2)
C(1)-C(6)	1.381(3)
C(1)-C(2)	1.394(3)
C(2)-O(2)	1.374(2)
C(2)-C(3)	1.385(3)
C(3)-O(3)	1.368(2)
C(3)-C(4)	1.381(3)
C(4)-C(5)	1.400(3)
C(4)-H(4)	0.9300
C(5)-C(6)	1.384(2)
C(5)-C(10)	1.459(3)
C(6)-H(6)	0.9300
C(7)-O(1)	1.423(3)
C(7)-H(7A)	0.9600
C(7)-H(7B)	0.9600
C(7)-H(7C)	0.9600
C(8)-O(2)	1.396(3)
C(8)-H(8A)	0.9600
C(8)-H(8B)	0.9600
C(8)-H(8C)	0.9600
C(9)-O(3)	1.422(2)
C(9)-H(9A)	0.9600
C(9)-H(9B)	0.9600
C(9)-H(9C)	0.9600
C(10)-C(11)	1.317(2)
C(10)-H(10)	0.9300
C(11)-C(12)	1.466(3)
C(11)-H(11)	0.9300
C(12)-O(4)	1.208(2)
C(12)-O(5)	1.324(2)
C(13)-O(5)	1.449(2)
C(13)-C(14)	1.479(2)
C(13)-H(13A)	0.9700
C(13)-H(13B)	0.9700

C(14)-C(15)	1.517(2)
C(14)-H(14A)	0.9700
C(14)-H(14B)	0.9700
C(15)-C(16)	1.516(2)
C(15)-H(15A)	0.9700
C(15)-H(15B)	0.9700
C(16)-C(17)	1.518(3)
C(16)-H(16A)	0.9700
C(16)-H(16B)	0.9700
C(17)-N(1)	1.456(2)
C(17)-H(17A)	0.9700
C(17)-H(17B)	0.9700
C(18)-N(1)	1.384(2)
C(18)-C(19)	1.400(3)
C(18)-C(22)	1.414(2)
C(19)-N(2)	1.324(3)
C(19)-C(30)	1.510(3)
C(20)-N(2)	1.332(3)
C(20)-C(21)	1.366(3)
C(20)-H(20)	0.9300
C(21)-C(22)	1.379(3)
C(21)-H(21)	0.9300
C(22)-C(23)	1.434(3)
C(23)-C(28)	1.394(3)
C(23)-C(24)	1.400(3)
C(24)-C(25)	1.366(3)
C(24)-H(24)	0.9300
C(25)-C(26)	1.388(3)
C(25)-H(25)	0.9300
C(26)-O(6)	1.370(3)
C(26)-C(27)	1.376(3)
C(27)-C(28)	1.396(3)
C(27)-H(27)	0.9300
C(28)-N(1)	1.384(2)
C(29)-O(6)	1.400(3)
C(29)-H(29A)	0.9600

C(29)-H(29B)	0.9600
C(29)-H(29C)	0.9600
C(30)-H(30A)	0.9600
C(30)-H(30B)	0.9600
C(30)-H(30C)	0.9600
O(1)-C(1)-C(6)	124.9(2)
O(1)-C(1)-C(2)	115.58(19)
C(6)-C(1)-C(2)	119.49(19)
O(2)-C(2)-C(3)	120.0(2)
O(2)-C(2)-C(1)	120.0(2)
C(3)-C(2)-C(1)	119.71(18)
O(3)-C(3)-C(4)	124.07(18)
O(3)-C(3)-C(2)	115.16(18)
C(4)-C(3)-C(2)	120.8(2)
C(3)-C(4)-C(5)	119.56(18)
C(3)-C(4)-H(4)	120.2
C(5)-C(4)-H(4)	120.2
C(6)-C(5)-C(4)	119.42(18)
C(6)-C(5)-C(10)	118.71(18)
C(4)-C(5)-C(10)	121.87(17)
C(1)-C(6)-C(5)	121.0(2)
C(1)-C(6)-H(6)	119.5
C(5)-C(6)-H(6)	119.5
O(1)-C(7)-H(7A)	109.5
O(1)-C(7)-H(7B)	109.5
H(7A)-C(7)-H(7B)	109.5
O(1)-C(7)-H(7C)	109.5
H(7A)-C(7)-H(7C)	109.5
H(7B)-C(7)-H(7C)	109.5
O(2)-C(8)-H(8A)	109.5
O(2)-C(8)-H(8B)	109.5
H(8A)-C(8)-H(8B)	109.5
O(2)-C(8)-H(8C)	109.5
H(8A)-C(8)-H(8C)	109.5
H(8B)-C(8)-H(8C)	109.5

O(3)-C(9)-H(9A)	109.5
O(3)-C(9)-H(9B)	109.5
H(9A)-C(9)-H(9B)	109.5
O(3)-C(9)-H(9C)	109.5
H(9A)-C(9)-H(9C)	109.5
H(9B)-C(9)-H(9C)	109.5
C(11)-C(10)-C(5)	127.33(18)
C(11)-C(10)-H(10)	116.3
C(5)-C(10)-H(10)	116.3
C(10)-C(11)-C(12)	124.96(18)
C(10)-C(11)-H(11)	117.5
C(12)-C(11)-H(11)	117.5
O(4)-C(12)-O(5)	122.52(18)
O(4)-C(12)-C(11)	123.63(18)
O(5)-C(12)-C(11)	113.85(16)
O(5)-C(13)-C(14)	108.07(14)
O(5)-C(13)-H(13A)	110.1
C(14)-C(13)-H(13A)	110.1
O(5)-C(13)-H(13B)	110.1
C(14)-C(13)-H(13B)	110.1
H(13A)-C(13)-H(13B)	108.4
C(13)-C(14)-C(15)	111.07(15)
C(13)-C(14)-H(14A)	109.4
C(15)-C(14)-H(14A)	109.4
C(13)-C(14)-H(14B)	109.4
C(15)-C(14)-H(14B)	109.4
H(14A)-C(14)-H(14B)	108.0
C(16)-C(15)-C(14)	112.98(15)
C(16)-C(15)-H(15A)	109.0
C(14)-C(15)-H(15A)	109.0
C(16)-C(15)-H(15B)	109.0
C(14)-C(15)-H(15B)	109.0
H(15A)-C(15)-H(15B)	107.8
C(15)-C(16)-C(17)	112.11(16)
C(15)-C(16)-H(16A)	109.2
C(17)-C(16)-H(16A)	109.2

C(15)-C(16)-H(16B)	109.2
C(17)-C(16)-H(16B)	109.2
H(16A)-C(16)-H(16B)	107.9
N(1)-C(17)-C(16)	113.75(15)
N(1)-C(17)-H(17A)	108.8
C(16)-C(17)-H(17A)	108.8
N(1)-C(17)-H(17B)	108.8
C(16)-C(17)-H(17B)	108.8
H(17A)-C(17)-H(17B)	107.7
N(1)-C(18)-C(19)	132.01(18)
N(1)-C(18)-C(22)	108.71(17)
C(19)-C(18)-C(22)	119.27(19)
N(2)-C(19)-C(18)	120.3(2)
N(2)-C(19)-C(30)	115.43(19)
C(18)-C(19)-C(30)	124.28(19)
N(2)-C(20)-C(21)	124.7(2)
N(2)-C(20)-H(20)	117.7
C(21)-C(20)-H(20)	117.7
C(20)-C(21)-C(22)	117.5(2)
C(20)-C(21)-H(21)	121.3
C(22)-C(21)-H(21)	121.3
C(21)-C(22)-C(18)	118.7(2)
C(21)-C(22)-C(23)	134.8(2)
C(18)-C(22)-C(23)	106.50(17)
C(28)-C(23)-C(24)	118.9(2)
C(28)-C(23)-C(22)	107.04(16)
C(24)-C(23)-C(22)	134.1(2)
C(25)-C(24)-C(23)	119.1(2)
C(25)-C(24)-H(24)	120.5
C(23)-C(24)-H(24)	120.5
C(24)-C(25)-C(26)	120.9(2)
C(24)-C(25)-H(25)	119.6
C(26)-C(25)-H(25)	119.6
O(6)-C(26)-C(27)	122.9(2)
O(6)-C(26)-C(25)	115.0(2)
C(27)-C(26)-C(25)	122.1(2)

C(26)-C(27)-C(28)	116.5(2)
C(26)-C(27)-H(27)	121.8
C(28)-C(27)-H(27)	121.8
N(1)-C(28)-C(23)	109.38(17)
N(1)-C(28)-C(27)	128.07(18)
C(23)-C(28)-C(27)	122.54(18)
O(6)-C(29)-H(29A)	109.5
O(6)-C(29)-H(29B)	109.5
H(29A)-C(29)-H(29B)	109.5
O(6)-C(29)-H(29C)	109.5
H(29A)-C(29)-H(29C)	109.5
H(29B)-C(29)-H(29C)	109.5
C(19)-C(30)-H(30A)	109.5
C(19)-C(30)-H(30B)	109.5
H(30A)-C(30)-H(30B)	109.5
C(19)-C(30)-H(30C)	109.5
H(30A)-C(30)-H(30C)	109.5
H(30B)-C(30)-H(30C)	109.5
C(28)-N(1)-C(18)	108.34(15)
C(28)-N(1)-C(17)	121.45(16)
C(18)-N(1)-C(17)	130.16(15)
C(19)-N(2)-C(20)	119.5(2)
C(1)-O(1)-C(7)	117.42(19)
C(2)-O(2)-C(8)	116.07(19)
C(3)-O(3)-C(9)	117.60(15)
C(12)-O(5)-C(13)	115.74(14)
C(26)-O(6)-C(29)	118.63(18)

Symmetry transformations used to generate equivalent atoms:

S 10. Anisotropic displacement parameters ($Å^2$x 10^3) for 7h. The anisotropic displacement factor exponent takes the form: $-2\pi^2[h^2 a^{*2}U^{11} + ... + 2 h k a^* b^* U^{12}]$

	U^{11}	U^{22}	U^{33}	U^{23}	U^{13}	U^{12}
C(1)	52(1)	66(1)	67(1)	-5(1)	4(1)	-15(1)
C(2)	59(1)	62(1)	58(1)	-11(1)	8(1)	-3(1)
C(3)	63(1)	53(1)	50(1)	-14(1)	-2(1)	-12(1)
C(4)	53(1)	54(1)	49(1)	-15(1)	-1(1)	-12(1)
C(5)	54(1)	52(1)	47(1)	-8(1)	-4(1)	-11(1)
C(6)	60(1)	59(1)	62(1)	-12(1)	-6(1)	-19(1)
C(7)	73(2)	110(2)	133(3)	-9(2)	6(2)	-45(2)
C(8)	109(2)	120(2)	62(2)	-25(2)	14(1)	-41(2)
C(9)	85(2)	77(2)	66(2)	-30(1)	1(1)	-30(1)
C(10)	57(1)	49(1)	53(1)	-13(1)	-8(1)	-16(1)
C(11)	64(1)	58(1)	53(1)	-23(1)	-2(1)	-23(1)
C(12)	58(1)	56(1)	53(1)	-17(1)	-4(1)	-24(1)
C(13)	52(1)	56(1)	49(1)	-18(1)	-3(1)	-16(1)
C(14)	52(1)	58(1)	50(1)	-19(1)	1(1)	-21(1)
C(15)	48(1)	57(1)	50(1)	-18(1)	1(1)	-15(1)
C(16)	52(1)	55(1)	59(1)	-22(1)	-4(1)	-14(1)
C(17)	52(1)	61(1)	48(1)	-12(1)	-1(1)	-22(1)
C(18)	51(1)	54(1)	51(1)	-13(1)	-7(1)	-13(1)
C(19)	68(1)	69(1)	53(1)	-21(1)	-4(1)	-15(1)
C(20)	113(2)	67(2)	83(2)	-27(1)	-20(2)	-31(2)
C(21)	82(2)	58(1)	83(2)	-8(1)	-16(1)	-32(1)
C(22)	57(1)	49(1)	61(1)	-6(1)	-12(1)	-15(1)
C(23)	47(1)	53(1)	56(1)	-1(1)	-2(1)	-13(1)
C(24)	60(1)	64(1)	68(2)	8(1)	-4(1)	-17(1)
C(25)	57(1)	78(2)	60(1)	1(1)	7(1)	-15(1)
C(26)	59(1)	74(2)	46(1)	-9(1)	2(1)	0(1)
C(27)	53(1)	66(1)	50(1)	-13(1)	1(1)	-12(1)
C(28)	43(1)	56(1)	49(1)	-10(1)	-1(1)	-12(1)
C(29)	102(2)	98(2)	61(2)	-32(2)	-6(1)	3(2)
C(30)	81(2)	122(2)	60(1)	-41(1)	19(1)	-35(2)
N(1)	50(1)	64(1)	46(1)	-19(1)	4(1)	-25(1)

N(2)	96(2)	76(1)	69(1)	-32(1)	-8(1)	-26(1)
O(1)	65(1)	105(1)	94(1)	-18(1)	12(1)	-33(1)
O(2)	85(1)	89(1)	70(1)	-21(1)	20(1)	-4(1)
O(3)	78(1)	78(1)	60(1)	-33(1)	8(1)	-20(1)
O(4)	73(1)	92(1)	74(1)	-41(1)	15(1)	-45(1)
O(5)	62(1)	63(1)	52(1)	-25(1)	3(1)	-26(1)
O(6)	86(1)	106(1)	55(1)	-24(1)	13(1)	-12(1)

S 11. Hydrogen coordinates (x 10^4) and isotropic displacement parameters ($Å^2$x 10^3) for 7h.

	x	y	z	U(eq)
H(4)	2981	-367	3175	63
H(6)	6459	1358	3388	71
H(7A)	8303	2195	2301	155
H(7B)	10290	1630	1985	155
H(7C)	9400	1238	2967	155
H(8A)	6579	-169	76	143
H(8B)	8613	-677	-158	143
H(8C)	7910	466	272	143
H(9A)	2952	-1932	2563	110
H(9B)	2872	-2043	1519	110
H(9C)	2091	-873	1899	110
H(10)	3964	1502	4519	62
H(11)	1714	208	4508	67
H(13A)	800	1894	7198	61
H(13B)	-336	2864	6551	61
H(14A)	1879	3811	6332	61
H(14B)	2980	2851	6996	61
H(15A)	-403	4153	7625	61
H(15B)	918	3316	8262	61
H(16A)	703	5312	8240	65
H(16B)	1784	5245	7247	65
H(17A)	2990	4218	8954	62
H(17B)	3957	3700	8043	62
H(20)	5596	7963	9871	100
H(21)	7067	7633	8431	85
H(24)	8313	6777	6638	79
H(25)	8852	5767	5388	82
H(27)	5261	4079	6561	69
H(29A)	7125	2826	5549	136
H(29B)	7263	3098	4461	136

H(29C)	5564	3687	5082	136
H(30A)	1558	6203	10590	128
H(30B)	2426	5035	10217	128
H(30C)	1134	5982	9636	128

S 12. Torsion angles [°] for 7h.

O(1)-C(1)-C(2)-O(2)	7.5(3)
C(6)-C(1)-C(2)-O(2)	-171.86(19)
O(1)-C(1)-C(2)-C(3)	-178.66(18)
C(6)-C(1)-C(2)-C(3)	2.0(3)
O(2)-C(2)-C(3)-O(3)	-7.7(3)
C(1)-C(2)-C(3)-O(3)	178.50(19)
O(2)-C(2)-C(3)-C(4)	171.77(19)
C(1)-C(2)-C(3)-C(4)	-2.1(3)
O(3)-C(3)-C(4)-C(5)	179.41(18)
C(2)-C(3)-C(4)-C(5)	0.0(3)
C(3)-C(4)-C(5)-C(6)	2.1(3)
C(3)-C(4)-C(5)-C(10)	-177.70(18)
O(1)-C(1)-C(6)-C(5)	-179.16(19)
C(2)-C(1)-C(6)-C(5)	0.1(3)
C(4)-C(5)-C(6)-C(1)	-2.2(3)
C(10)-C(5)-C(6)-C(1)	177.61(19)
C(6)-C(5)-C(10)-C(11)	-175.3(2)
C(4)-C(5)-C(10)-C(11)	4.4(3)
C(5)-C(10)-C(11)-C(12)	-179.11(19)
C(10)-C(11)-C(12)-O(4)	179.7(2)
C(10)-C(11)-C(12)-O(5)	-0.1(3)
O(5)-C(13)-C(14)-C(15)	178.72(15)
C(13)-C(14)-C(15)-C(16)	-170.57(16)
C(14)-C(15)-C(16)-C(17)	-77.6(2)
C(15)-C(16)-C(17)-N(1)	158.47(15)
N(1)-C(18)-C(19)-N(2)	176.6(2)
C(22)-C(18)-C(19)-N(2)	-4.4(3)
N(1)-C(18)-C(19)-C(30)	-4.6(4)
C(22)-C(18)-C(19)-C(30)	174.4(2)
N(2)-C(20)-C(21)-C(22)	-2.2(4)
C(20)-C(21)-C(22)-C(18)	-0.4(3)
C(20)-C(21)-C(22)-C(23)	-178.8(2)
N(1)-C(18)-C(22)-C(21)	-177.18(18)
C(19)-C(18)-C(22)-C(21)	3.6(3)

N(1)-C(18)-C(22)-C(23)	1.7(2)
C(19)-C(18)-C(22)-C(23)	-177.61(18)
C(21)-C(22)-C(23)-C(28)	177.0(2)
C(18)-C(22)-C(23)-C(28)	-1.6(2)
C(21)-C(22)-C(23)-C(24)	-2.2(4)
C(18)-C(22)-C(23)-C(24)	179.2(2)
C(28)-C(23)-C(24)-C(25)	-0.4(3)
C(22)-C(23)-C(24)-C(25)	178.7(2)
C(23)-C(24)-C(25)-C(26)	-0.6(3)
C(24)-C(25)-C(26)-O(6)	-179.89(19)
C(24)-C(25)-C(26)-C(27)	1.2(3)
O(6)-C(26)-C(27)-C(28)	-179.41(18)
C(25)-C(26)-C(27)-C(28)	-0.6(3)
C(24)-C(23)-C(28)-N(1)	-179.73(17)
C(22)-C(23)-C(28)-N(1)	0.9(2)
C(24)-C(23)-C(28)-C(27)	1.1(3)
C(22)-C(23)-C(28)-C(27)	-178.32(18)
C(26)-C(27)-C(28)-N(1)	-179.60(19)
C(26)-C(27)-C(28)-C(23)	-0.5(3)
C(23)-C(28)-N(1)-C(18)	0.1(2)
C(27)-C(28)-N(1)-C(18)	179.29(19)
C(23)-C(28)-N(1)-C(17)	-177.57(16)
C(27)-C(28)-N(1)-C(17)	1.6(3)
C(19)-C(18)-N(1)-C(28)	178.0(2)
C(22)-C(18)-N(1)-C(28)	-1.1(2)
C(19)-C(18)-N(1)-C(17)	-4.6(3)
C(22)-C(18)-N(1)-C(17)	176.30(17)
C(16)-C(17)-N(1)-C(28)	-85.8(2)
C(16)-C(17)-N(1)-C(18)	97.0(2)
C(18)-C(19)-N(2)-C(20)	1.9(3)
C(30)-C(19)-N(2)-C(20)	-177.0(2)
C(21)-C(20)-N(2)-C(19)	1.5(4)
C(6)-C(1)-O(1)-C(7)	-5.1(3)
C(2)-C(1)-O(1)-C(7)	175.6(2)
C(3)-C(2)-O(2)-C(8)	99.6(3)
C(1)-C(2)-O(2)-C(8)	-86.6(3)

C(4)-C(3)-O(3)-C(9)	5.5(3)
C(2)-C(3)-O(3)-C(9)	-175.07(18)
O(4)-C(12)-O(5)-C(13)	-1.5(3)
C(11)-C(12)-O(5)-C(13)	178.23(16)
C(14)-C(13)-O(5)-C(12)	-179.65(16)
C(27)-C(26)-O(6)-C(29)	-5.3(3)
C(25)-C(26)-O(6)-C(29)	175.8(2)

Symmetry transformations used to generate equivalent atoms:

S 13. Crystal data and structure refinement for 11b.

Identification code	1_a	
Empirical formula	C19 H17 Cl N2 O	
Formula weight	324.79	
Temperature	296(2) K	
Wavelength	71.073 pm	
Crystal system	Monoclinic	
Space group	$P2_1/c$	
Unit cell dimensions	a = 1487.3(3) pm	α= 90°.
	b = 947.60(19) pm	β= 107.846(3)°.
	c = 1219.4(2) pm	γ = 90°.
Volume	1.6359(6) nm^3	
Z	4	
Density (calculated)	1.319 Mg/m^3	
Absorption coefficient	0.239 mm^{-1}	
F(000)	680	
Crystal size	0.300 x 0.200 x 0.200 mm^3	
Theta range for data collection	1.438 to 31.242°.	
Index ranges	-19<=h<=18, -13<=k<=12, -15<=l<=17	
Reflections collected	11140	
Independent reflections	4719 [R(int) = 0.0295]	
Completeness to theta = 25.242°	99.9 %	
Absorption correction	Semi-empirical from equivalents	
Refinement method	Full-matrix least-squares on F^2	
Data / restraints / parameters	4719 / 0 / 209	
Goodness-of-fit on F^2	0.999	
Final R indices [I>2sigma(I)]	R1 = 0.0500, wR2 = 0.1431	
R indices (all data)	R1 = 0.0915, wR2 = 0.1755	
Extinction coefficient	n/a	
Largest diff. peak and hole	0.314 and -0.417 e.$Å^{-3}$	

S 14. Atomic coordinates (x 10^4) and equivalent isotropic displacement parameters (pm^2x 10^{-1}) for 11b. U(eq) is defined as one third of the trace of the orthogonalized U^{ij} tensor.

	x	y	z	U(eq)
C(1)	7713(1)	5861(2)	5275(1)	42(1)
C(2)	7738(1)	7277(2)	4899(2)	45(1)
C(3)	7084(1)	8198(2)	4180(2)	55(1)
C(4)	7368(2)	9511(2)	3974(2)	68(1)
C(5)	8298(2)	9973(2)	4481(2)	67(1)
C(6)	8956(2)	9098(2)	5203(2)	55(1)
C(7)	8671(1)	7766(2)	5405(2)	44(1)
C(8)	8598(1)	5546(2)	5970(1)	41(1)
C(9)	8940(1)	4161(2)	6533(1)	41(1)
C(10)	7419(1)	3332(2)	5152(1)	44(1)
C(11)	6954(1)	4757(2)	4969(2)	49(1)
C(12)	9710(1)	3525(2)	6109(2)	56(1)
C(13)	8136(1)	2194(2)	7077(1)	40(1)
C(14)	7325(1)	1197(2)	6891(1)	40(1)
C(15)	7538(1)	-226(2)	7008(2)	48(1)
C(16)	6834(2)	-1220(2)	6861(2)	55(1)
C(17)	5920(1)	-785(2)	6627(2)	59(1)
C(18)	5690(1)	638(3)	6541(2)	65(1)
C(19)	6393(1)	1610(2)	6668(2)	54(1)
Cl(1)	5032(1)	-2029(1)	6465(1)	105(1)
N(1)	9180(1)	6689(2)	6065(1)	45(1)
N(2)	8111(1)	3225(2)	6308(1)	40(1)
O(1)	8822(1)	2042(1)	7953(1)	51(1)

S 15. Bond lengths [pm] and angles [°] for 11b.

C(1)-C(8)	136.1(2)
C(1)-C(2)	142.1(2)
C(1)-C(11)	150.0(2)
C(2)-C(3)	139.8(2)
C(2)-C(7)	141.3(3)
C(3)-C(4)	136.2(3)
C(3)-H(3)	93.00
C(4)-C(5)	140.2(3)
C(4)-H(4)	93.00
C(5)-C(6)	137.6(3)
C(5)-H(5)	93.00
C(6)-C(7)	137.7(3)
C(6)-H(6)	93.00
C(7)-N(1)	137.4(2)
C(8)-N(1)	136.9(2)
C(8)-C(9)	149.6(2)
C(9)-N(2)	147.5(2)
C(9)-C(12)	151.8(3)
C(9)-H(9)	98.00
C(10)-N(2)	147.3(2)
C(10)-C(11)	150.2(3)
C(10)-H(10A)	97.00
C(10)-H(10B)	97.00
C(11)-H(11A)	97.00
C(11)-H(11B)	97.00
C(12)-H(12A)	96.00
C(12)-H(12B)	96.00
C(12)-H(12C)	96.00
C(13)-O(1)	123.8(2)
C(13)-N(2)	134.7(2)
C(13)-C(14)	149.5(2)
C(14)-C(15)	138.3(3)
C(14)-C(19)	138.4(2)
C(15)-C(16)	137.9(3)

C(15)-H(15)	93.00
C(16)-C(17)	136.4(3)
C(16)-H(16)	93.00
C(17)-C(18)	138.7(3)
C(17)-Cl(1)	173.5(2)
C(18)-C(19)	136.7(3)
C(18)-H(18)	93.00
C(19)-H(19)	93.00
N(1)-H(1)	86.00
C(8)-C(1)-C(2)	107.02(15)
C(8)-C(1)-C(11)	120.77(16)
C(2)-C(1)-C(11)	132.01(15)
C(3)-C(2)-C(7)	118.08(17)
C(3)-C(2)-C(1)	135.21(18)
C(7)-C(2)-C(1)	106.71(15)
C(4)-C(3)-C(2)	119.4(2)
C(4)-C(3)-H(3)	120.3
C(2)-C(3)-H(3)	120.3
C(3)-C(4)-C(5)	121.6(2)
C(3)-C(4)-H(4)	119.2
C(5)-C(4)-H(4)	119.2
C(6)-C(5)-C(4)	120.4(2)
C(6)-C(5)-H(5)	119.8
C(4)-C(5)-H(5)	119.8
C(5)-C(6)-C(7)	118.0(2)
C(5)-C(6)-H(6)	121.0
C(7)-C(6)-H(6)	121.0
N(1)-C(7)-C(6)	129.82(17)
N(1)-C(7)-C(2)	107.64(15)
C(6)-C(7)-C(2)	122.53(17)
C(1)-C(8)-N(1)	110.12(15)
C(1)-C(8)-C(9)	127.33(15)
N(1)-C(8)-C(9)	122.51(14)
N(2)-C(9)-C(8)	107.14(12)
N(2)-C(9)-C(12)	111.89(15)

C(8)-C(9)-C(12)	112.23(15)
N(2)-C(9)-H(9)	108.5
C(8)-C(9)-H(9)	108.5
C(12)-C(9)-H(9)	108.5
N(2)-C(10)-C(11)	111.04(14)
N(2)-C(10)-H(10A)	109.4
C(11)-C(10)-H(10A)	109.4
N(2)-C(10)-H(10B)	109.4
C(11)-C(10)-H(10B)	109.4
H(10A)-C(10)-H(10B)	108.0
C(1)-C(11)-C(10)	108.23(14)
C(1)-C(11)-H(11A)	110.1
C(10)-C(11)-H(11A)	110.1
C(1)-C(11)-H(11B)	110.1
C(10)-C(11)-H(11B)	110.1
H(11A)-C(11)-H(11B)	108.4
C(9)-C(12)-H(12A)	109.5
C(9)-C(12)-H(12B)	109.5
H(12A)-C(12)-H(12B)	109.5
C(9)-C(12)-H(12C)	109.5
H(12A)-C(12)-H(12C)	109.5
H(12B)-C(12)-H(12C)	109.5
O(1)-C(13)-N(2)	121.68(16)
O(1)-C(13)-C(14)	118.07(15)
N(2)-C(13)-C(14)	120.25(14)
C(15)-C(14)-C(19)	118.71(17)
C(15)-C(14)-C(13)	116.82(15)
C(19)-C(14)-C(13)	124.40(16)
C(16)-C(15)-C(14)	120.74(17)
C(16)-C(15)-H(15)	119.6
C(14)-C(15)-H(15)	119.6
C(17)-C(16)-C(15)	119.34(19)
C(17)-C(16)-H(16)	120.3
C(15)-C(16)-H(16)	120.3
C(16)-C(17)-C(18)	121.07(18)
C(16)-C(17)-Cl(1)	119.58(18)

C(18)-C(17)-Cl(1)	119.33(16)
C(19)-C(18)-C(17)	118.95(18)
C(19)-C(18)-H(18)	120.5
C(17)-C(18)-H(18)	120.5
C(18)-C(19)-C(14)	121.14(19)
C(18)-C(19)-H(19)	119.4
C(14)-C(19)-H(19)	119.4
C(8)-N(1)-C(7)	108.49(14)
C(8)-N(1)-H(1)	125.8
C(7)-N(1)-H(1)	125.8
C(13)-N(2)-C(10)	125.39(14)
C(13)-N(2)-C(9)	117.63(13)
C(10)-N(2)-C(9)	115.81(13)

Symmetry transformations used to generate equivalent atoms:

S 16. Anisotropic displacement parameters ($pm^2 x 10^{-1}$) for 11b. The anisotropic displacement factor exponent takes the form: $-2\pi^2[h^2 a^{*2}U^{11} + ... + 2 h k a^* b^* U^{12}]$

	U^{11}	U^{22}	U^{33}	U^{23}	U^{13}	U^{12}
C(1)	40(1)	40(1)	42(1)	-3(1)	6(1)	0(1)
C(2)	48(1)	42(1)	41(1)	-2(1)	10(1)	5(1)
C(3)	59(1)	49(1)	53(1)	-2(1)	9(1)	11(1)
C(4)	90(2)	51(1)	59(1)	9(1)	18(1)	22(1)
C(5)	92(2)	44(1)	72(1)	6(1)	35(1)	2(1)
C(6)	63(1)	43(1)	63(1)	1(1)	25(1)	-5(1)
C(7)	49(1)	42(1)	44(1)	-2(1)	17(1)	1(1)
C(8)	38(1)	43(1)	39(1)	-1(1)	9(1)	-3(1)
C(9)	36(1)	42(1)	38(1)	2(1)	3(1)	-5(1)
C(10)	46(1)	43(1)	36(1)	1(1)	1(1)	-5(1)
C(11)	40(1)	49(1)	47(1)	3(1)	-2(1)	-2(1)
C(12)	48(1)	54(1)	64(1)	9(1)	17(1)	4(1)
C(13)	38(1)	42(1)	40(1)	0(1)	11(1)	3(1)
C(14)	40(1)	41(1)	37(1)	3(1)	10(1)	0(1)
C(15)	48(1)	50(1)	49(1)	3(1)	18(1)	1(1)
C(16)	69(1)	45(1)	54(1)	2(1)	22(1)	-8(1)
C(17)	54(1)	72(1)	46(1)	7(1)	7(1)	-21(1)
C(18)	36(1)	86(2)	70(1)	15(1)	11(1)	-3(1)
C(19)	45(1)	56(1)	62(1)	6(1)	15(1)	5(1)
Cl(1)	78(1)	118(1)	98(1)	31(1)	-4(1)	-54(1)
N(1)	40(1)	44(1)	48(1)	1(1)	8(1)	-5(1)
N(2)	38(1)	40(1)	36(1)	2(1)	3(1)	-4(1)
O(1)	42(1)	58(1)	46(1)	10(1)	1(1)	-2(1)

S 17. Hydrogen coordinates (x 10^4) and isotropic displacement parameters (pm^2x 10^{-1}) for 11b.

	x	y	z	U(eq)
H(3)	6460	7916	3846	66
H(4)	6933	10115	3484	81
H(5)	8472	10878	4328	81
H(6)	9574	9396	5545	66
H(9)	9196	4313	7366	49
H(10A)	6941	2607	5061	53
H(10B)	7735	3176	4574	53
H(11A)	6572	4863	4171	59
H(11B)	6547	4858	5452	59
H(12A)	9899	2632	6479	83
H(12B)	10243	4150	6290	83
H(12C)	9474	3392	5290	83
H(15)	8164	-516	7188	58
H(16)	6980	-2176	6921	66
H(18)	5067	925	6401	78
H(19)	6243	2565	6602	65
H(1)	9765	6727	6470	54

S 18. Torsion angles [°] for 11b.

C(8)-C(1)-C(2)-C(3)	179.0(2)
C(11)-C(1)-C(2)-C(3)	4.3(4)
C(8)-C(1)-C(2)-C(7)	-0.1(2)
C(11)-C(1)-C(2)-C(7)	-174.78(18)
C(7)-C(2)-C(3)-C(4)	1.3(3)
C(1)-C(2)-C(3)-C(4)	-177.7(2)
C(2)-C(3)-C(4)-C(5)	-1.1(3)
C(3)-C(4)-C(5)-C(6)	0.4(4)
C(4)-C(5)-C(6)-C(7)	0.2(3)
C(5)-C(6)-C(7)-N(1)	178.89(19)
C(5)-C(6)-C(7)-C(2)	0.1(3)
C(3)-C(2)-C(7)-N(1)	-179.85(16)
C(1)-C(2)-C(7)-N(1)	-0.6(2)
C(3)-C(2)-C(7)-C(6)	-0.8(3)
C(1)-C(2)-C(7)-C(6)	178.48(17)
C(2)-C(1)-C(8)-N(1)	0.7(2)
C(11)-C(1)-C(8)-N(1)	176.12(15)
C(2)-C(1)-C(8)-C(9)	-176.99(17)
C(11)-C(1)-C(8)-C(9)	-1.5(3)
C(1)-C(8)-C(9)-N(2)	-6.4(2)
N(1)-C(8)-C(9)-N(2)	176.22(15)
C(1)-C(8)-C(9)-C(12)	116.81(19)
N(1)-C(8)-C(9)-C(12)	-60.6(2)
C(8)-C(1)-C(11)-C(10)	-20.8(2)
C(2)-C(1)-C(11)-C(10)	153.31(19)
N(2)-C(10)-C(11)-C(1)	51.3(2)
O(1)-C(13)-C(14)-C(15)	-48.9(2)
N(2)-C(13)-C(14)-C(15)	131.28(17)
O(1)-C(13)-C(14)-C(19)	127.87(19)
N(2)-C(13)-C(14)-C(19)	-51.9(2)
C(19)-C(14)-C(15)-C(16)	2.5(3)
C(13)-C(14)-C(15)-C(16)	179.45(16)
C(14)-C(15)-C(16)-C(17)	-1.7(3)
C(15)-C(16)-C(17)-C(18)	-0.4(3)

C(15)-C(16)-C(17)-Cl(1)	-178.93(15)
C(16)-C(17)-C(18)-C(19)	1.6(3)
Cl(1)-C(17)-C(18)-C(19)	-179.85(16)
C(17)-C(18)-C(19)-C(14)	-0.8(3)
C(15)-C(14)-C(19)-C(18)	-1.2(3)
C(13)-C(14)-C(19)-C(18)	-177.97(18)
C(1)-C(8)-N(1)-C(7)	-1.0(2)
C(9)-C(8)-N(1)-C(7)	176.75(16)
C(6)-C(7)-N(1)-C(8)	-177.98(19)
C(2)-C(7)-N(1)-C(8)	1.0(2)
O(1)-C(13)-N(2)-C(10)	168.10(16)
C(14)-C(13)-N(2)-C(10)	-12.1(3)
O(1)-C(13)-N(2)-C(9)	1.1(2)
C(14)-C(13)-N(2)-C(9)	-179.16(14)
C(11)-C(10)-N(2)-C(13)	127.29(18)
C(11)-C(10)-N(2)-C(9)	-65.46(19)
C(8)-C(9)-N(2)-C(13)	-152.68(15)
C(12)-C(9)-N(2)-C(13)	83.91(19)
C(8)-C(9)-N(2)-C(10)	39.03(19)
C(12)-C(9)-N(2)-C(10)	-84.38(18)

Symmetry transformations used to generate equivalent atoms:

S 19. Crystal data and structure refinement for 11f.

Identification code	1_a	
Empirical formula	C19 H19.50 N3 O3	
Formula weight	337.87	
Temperature	296(2) K	
Wavelength	0.71073 Å	
Crystal system	Monoclinic	
Space group	$P2_1/n$	
Unit cell dimensions	a = 8.3100(14) Å	α= 90°.
	b = 14.485(3) Å	β= 105.111(3)°.
	c = 14.462(2) Å	γ = 90°.
Volume	1680.6(5) $Å^3$	
Z	4	
Density (calculated)	1.335 Mg/m^3	
Absorption coefficient	0.092 mm^{-1}	
F(000)	714	
Crystal size	0.300 x 0.200 x 0.200 mm^3	
Theta range for data collection	2.026 to 30.510°.	
Index ranges	-9<=h<=11, -18<=k<=20, -19<=l<=18	
Reflections collected	11295	
Independent reflections	4748 [R(int) = 0.0229]	
Completeness to theta = 25.242°	99.9 %	
Absorption correction	Semi-empirical from equivalents	
Refinement method	Full-matrix least-squares on F^2	
Data / restraints / parameters	4748 / 0 / 227	
Goodness-of-fit on F^2	0.999	
Final R indices [I>2sigma(I)]	R1 = 0.0501, wR2 = 0.1171	
R indices (all data)	R1 = 0.0769, wR2 = 0.1309	
Extinction coefficient	n/a	
Largest diff. peak and hole	0.198 and -0.245 e.$Å^{-3}$	

S 20. Atomic coordinates (x 10^4) and equivalent isotropic displacement parameters (Å^{2}x 10^3) for 11f. U(eq) is defined as one third of the trace of the orthogonalized U^{ij} tensor.

	x	y	z	U(eq)
O(3)	4587(2)	3719(1)	6355(1)	54(1)
O(2)	1085(2)	3627(1)	6491(1)	64(1)
N(2)	3071(2)	3220(1)	4906(1)	42(1)
N(1)	3656(2)	4591(1)	2839(1)	49(1)
N(5)	1623(2)	3130(1)	7184(1)	58(1)
C(10)	3338(2)	3919(1)	3446(1)	43(1)
C(13)	3693(2)	3128(1)	5856(1)	42(1)
C(7)	2969(2)	3101(1)	2973(1)	44(1)
C(19)	3346(2)	2252(1)	6337(1)	43(1)
O(1)	1447(3)	3296(1)	7980(1)	101(1)
C(11)	3378(2)	4106(1)	4468(1)	43(1)
C(18)	2483(2)	2278(1)	7043(1)	47(1)
C(6)	3084(2)	3255(1)	2008(1)	45(1)
C(9)	1842(2)	2591(1)	4295(1)	48(1)
C(1)	3501(2)	4198(1)	1949(1)	47(1)
C(8)	2460(2)	2264(1)	3443(1)	52(1)
C(17)	2351(2)	1525(1)	7609(1)	59(1)
C(14)	4068(2)	1415(1)	6195(1)	56(1)
C(2)	3680(2)	4590(1)	1099(1)	61(1)
C(12)	2133(3)	4847(1)	4591(1)	60(1)
C(5)	2855(2)	2700(1)	1190(1)	54(1)
C(16)	3086(3)	705(1)	7451(1)	67(1)
C(15)	3926(2)	649(1)	6754(2)	66(1)
C(4)	3041(3)	3090(1)	354(1)	65(1)
C(3)	3438(3)	4024(1)	308(1)	69(1)

S 21. Bond lengths [Å] and angles [°] for 11f.

O(3)-C(13)	1.2353(16)
O(2)-N(5)	1.2199(18)
N(2)-C(13)	1.3423(18)
N(2)-C(9)	1.4773(18)
N(2)-C(11)	1.4817(17)
N(1)-C(10)	1.3826(18)
N(1)-C(1)	1.3829(19)
N(1)-H(1)	0.8600
N(5)-O(1)	1.2223(18)
N(5)-C(18)	1.466(2)
C(10)-C(7)	1.3631(19)
C(10)-C(11)	1.493(2)
C(13)-C(19)	1.5112(19)
C(7)-C(6)	1.441(2)
C(7)-C(8)	1.503(2)
C(19)-C(14)	1.392(2)
C(19)-C(18)	1.392(2)
C(11)-C(12)	1.533(2)
C(11)-H(11)	0.9800
C(18)-C(17)	1.386(2)
C(6)-C(5)	1.402(2)
C(6)-C(1)	1.417(2)
C(9)-C(8)	1.528(2)
C(9)-H(9A)	0.9700
C(9)-H(9B)	0.9700
C(1)-C(2)	1.396(2)
C(8)-H(8A)	0.9700
C(8)-H(8B)	0.9700
C(17)-C(16)	1.381(3)
C(17)-H(17)	0.9300
C(14)-C(15)	1.395(2)
C(14)-H(14)	0.9300
C(2)-C(3)	1.379(3)
C(2)-H(2)	0.9300

C(12)-H(12A)	0.9600
C(12)-H(12B)	0.9600
C(12)-H(12C)	0.9600
C(5)-C(4)	1.380(2)
C(5)-H(5)	0.9300
C(16)-C(15)	1.371(3)
C(16)-H(16)	0.9300
C(15)-H(15)	0.9300
C(4)-C(3)	1.398(3)
C(4)-H(4)	0.9300
C(3)-H(3)	0.9300
C(13)-N(2)-C(9)	124.90(12)
C(13)-N(2)-C(11)	117.43(11)
C(9)-N(2)-C(11)	116.56(11)
C(10)-N(1)-C(1)	108.32(12)
C(10)-N(1)-H(1)	125.8
C(1)-N(1)-H(1)	125.8
O(2)-N(5)-O(1)	123.32(16)
O(2)-N(5)-C(18)	118.07(13)
O(1)-N(5)-C(18)	118.58(15)
C(7)-C(10)-N(1)	110.38(13)
C(7)-C(10)-C(11)	126.71(13)
N(1)-C(10)-C(11)	122.89(12)
O(3)-C(13)-N(2)	122.79(13)
O(3)-C(13)-C(19)	118.08(13)
N(2)-C(13)-C(19)	119.09(12)
C(10)-C(7)-C(6)	106.78(12)
C(10)-C(7)-C(8)	121.80(13)
C(6)-C(7)-C(8)	131.32(13)
C(14)-C(19)-C(18)	117.17(14)
C(14)-C(19)-C(13)	121.51(14)
C(18)-C(19)-C(13)	120.71(13)
N(2)-C(11)-C(10)	107.74(11)
N(2)-C(11)-C(12)	111.13(13)
C(10)-C(11)-C(12)	113.56(13)

N(2)-C(11)-H(11)	108.1
C(10)-C(11)-H(11)	108.1
C(12)-C(11)-H(11)	108.1
C(17)-C(18)-C(19)	123.10(15)
C(17)-C(18)-N(5)	117.68(15)
C(19)-C(18)-N(5)	119.19(13)
C(5)-C(6)-C(1)	118.89(14)
C(5)-C(6)-C(7)	134.44(14)
C(1)-C(6)-C(7)	106.67(13)
N(2)-C(9)-C(8)	110.37(13)
N(2)-C(9)-H(9A)	109.6
C(8)-C(9)-H(9A)	109.6
N(2)-C(9)-H(9B)	109.6
C(8)-C(9)-H(9B)	109.6
H(9A)-C(9)-H(9B)	108.1
N(1)-C(1)-C(2)	130.21(15)
N(1)-C(1)-C(6)	107.83(13)
C(2)-C(1)-C(6)	121.96(15)
C(7)-C(8)-C(9)	107.95(12)
C(7)-C(8)-H(8A)	110.1
C(9)-C(8)-H(8A)	110.1
C(7)-C(8)-H(8B)	110.1
C(9)-C(8)-H(8B)	110.1
H(8A)-C(8)-H(8B)	108.4
C(16)-C(17)-C(18)	118.26(18)
C(16)-C(17)-H(17)	120.9
C(18)-C(17)-H(17)	120.9
C(19)-C(14)-C(15)	120.16(17)
C(19)-C(14)-H(14)	119.9
C(15)-C(14)-H(14)	119.9
C(3)-C(2)-C(1)	117.54(16)
C(3)-C(2)-H(2)	121.2
C(1)-C(2)-H(2)	121.2
C(11)-C(12)-H(12A)	109.5
C(11)-C(12)-H(12B)	109.5
H(12A)-C(12)-H(12B)	109.5

C(11)-C(12)-H(12C)	109.5
H(12A)-C(12)-H(12C)	109.5
H(12B)-C(12)-H(12C)	109.5
C(4)-C(5)-C(6)	118.89(16)
C(4)-C(5)-H(5)	120.6
C(6)-C(5)-H(5)	120.6
C(15)-C(16)-C(17)	120.30(16)
C(15)-C(16)-H(16)	119.9
C(17)-C(16)-H(16)	119.9
C(16)-C(15)-C(14)	121.00(17)
C(16)-C(15)-H(15)	119.5
C(14)-C(15)-H(15)	119.5
C(5)-C(4)-C(3)	121.33(17)
C(5)-C(4)-H(4)	119.3
C(3)-C(4)-H(4)	119.3
C(2)-C(3)-C(4)	121.39(17)
C(2)-C(3)-H(3)	119.3
C(4)-C(3)-H(3)	119.3

Symmetry transformations used to generate equivalent atoms:

S 22. Anisotropic displacement parameters ($Å^2$x 10^3) for 11f. The anisotropic displacement factor exponent takes the form: $-2\pi^2[h^2 a^{*2}U^{11} + ... + 2 h k a^* b^* U^{12}]$

	U^{11}	U^{22}	U^{33}	U^{23}	U^{13}	U^{12}
O(3)	66(1)	38(1)	49(1)	-2(1)	-1(1)	-14(1)
O(2)	75(1)	42(1)	72(1)	5(1)	16(1)	7(1)
N(2)	49(1)	32(1)	41(1)	0(1)	6(1)	-10(1)
N(1)	64(1)	32(1)	51(1)	2(1)	18(1)	-7(1)
N(5)	80(1)	40(1)	54(1)	-6(1)	20(1)	-9(1)
C(10)	50(1)	33(1)	45(1)	2(1)	11(1)	-5(1)
C(13)	47(1)	31(1)	45(1)	-1(1)	5(1)	-4(1)
C(7)	51(1)	35(1)	45(1)	-1(1)	11(1)	-6(1)
C(19)	48(1)	32(1)	43(1)	2(1)	-1(1)	-6(1)
O(1)	180(2)	68(1)	71(1)	-12(1)	61(1)	1(1)
C(11)	53(1)	30(1)	45(1)	0(1)	9(1)	-7(1)
C(18)	58(1)	34(1)	42(1)	1(1)	2(1)	-7(1)
C(6)	48(1)	41(1)	46(1)	-1(1)	12(1)	-1(1)
C(9)	53(1)	42(1)	44(1)	0(1)	5(1)	-16(1)
C(1)	51(1)	42(1)	49(1)	3(1)	14(1)	1(1)
C(8)	69(1)	35(1)	48(1)	-3(1)	10(1)	-15(1)
C(17)	72(1)	50(1)	50(1)	10(1)	5(1)	-13(1)
C(14)	55(1)	37(1)	71(1)	0(1)	10(1)	-1(1)
C(2)	73(1)	54(1)	60(1)	11(1)	26(1)	0(1)
C(12)	82(1)	43(1)	58(1)	1(1)	22(1)	9(1)
C(5)	60(1)	50(1)	53(1)	-6(1)	16(1)	0(1)
C(16)	72(1)	42(1)	74(1)	21(1)	0(1)	-7(1)
C(15)	61(1)	34(1)	94(1)	8(1)	3(1)	2(1)
C(4)	76(1)	73(1)	50(1)	-8(1)	22(1)	5(1)
C(3)	83(1)	75(1)	54(1)	11(1)	30(1)	7(1)

S 23. Hydrogen coordinates (x 10^4) and isotropic displacement parameters ($Å^2$x 10^{3}) for 11f.

	x	y	z	U(eq)
H(1)	3909	5158	2989	58
H(11)	4501	4317	4798	52
H(9A)	786	2909	4066	57
H(9B)	1666	2063	4669	57
H(8A)	3402	1851	3659	62
H(8B)	1580	1935	2991	62
H(17)	1782	1570	8082	71
H(14)	4646	1365	5726	67
H(2)	3952	5210	1068	73
H(12A)	1020	4645	4289	90
H(12B)	2356	5412	4299	90
H(12C)	2246	4949	5261	90
H(5)	2583	2079	1210	65
H(16)	3011	189	7820	80
H(15)	4409	92	6651	80
H(4)	2899	2725	-190	78
H(3)	3542	4269	-268	82

S 24. Torsion angles [°] for 11f.

C(1)-N(1)-C(10)-C(7)	-0.38(18)
C(1)-N(1)-C(10)-C(11)	-179.32(14)
C(9)-N(2)-C(13)-O(3)	-172.45(15)
C(11)-N(2)-C(13)-O(3)	-5.0(2)
C(9)-N(2)-C(13)-C(19)	10.0(2)
C(11)-N(2)-C(13)-C(19)	177.43(13)
N(1)-C(10)-C(7)-C(6)	0.93(18)
C(11)-C(10)-C(7)-C(6)	179.81(14)
N(1)-C(10)-C(7)-C(8)	-175.90(14)
C(11)-C(10)-C(7)-C(8)	3.0(3)
O(3)-C(13)-C(19)-C(14)	-107.54(18)
N(2)-C(13)-C(19)-C(14)	70.2(2)
O(3)-C(13)-C(19)-C(18)	63.2(2)
N(2)-C(13)-C(19)-C(18)	-119.06(16)
C(13)-N(2)-C(11)-C(10)	153.61(13)
C(9)-N(2)-C(11)-C(10)	-37.87(17)
C(13)-N(2)-C(11)-C(12)	-81.39(17)
C(9)-N(2)-C(11)-C(12)	87.13(16)
C(7)-C(10)-C(11)-N(2)	4.3(2)
N(1)-C(10)-C(11)-N(2)	-176.91(13)
C(7)-C(10)-C(11)-C(12)	-119.19(18)
N(1)-C(10)-C(11)-C(12)	59.6(2)
C(14)-C(19)-C(18)-C(17)	1.4(2)
C(13)-C(19)-C(18)-C(17)	-169.81(15)
C(14)-C(19)-C(18)-N(5)	-176.68(14)
C(13)-C(19)-C(18)-N(5)	12.1(2)
O(2)-N(5)-C(18)-C(17)	-148.00(16)
O(1)-N(5)-C(18)-C(17)	30.3(2)
O(2)-N(5)-C(18)-C(19)	30.2(2)
O(1)-N(5)-C(18)-C(19)	-151.58(17)
C(10)-C(7)-C(6)-C(5)	179.98(18)
C(8)-C(7)-C(6)-C(5)	-3.6(3)
C(10)-C(7)-C(6)-C(1)	-1.11(17)
C(8)-C(7)-C(6)-C(1)	175.30(16)

C(13)-N(2)-C(9)-C(8)	-128.13(15)
C(11)-N(2)-C(9)-C(8)	64.30(17)
C(10)-N(1)-C(1)-C(2)	179.21(17)
C(10)-N(1)-C(1)-C(6)	-0.34(18)
C(5)-C(6)-C(1)-N(1)	180.00(14)
C(7)-C(6)-C(1)-N(1)	0.89(17)
C(5)-C(6)-C(1)-C(2)	0.4(2)
C(7)-C(6)-C(1)-C(2)	-178.71(15)
C(10)-C(7)-C(8)-C(9)	20.4(2)
C(6)-C(7)-C(8)-C(9)	-155.57(16)
N(2)-C(9)-C(8)-C(7)	-50.58(17)
C(19)-C(18)-C(17)-C(16)	-1.2(3)
N(5)-C(18)-C(17)-C(16)	176.89(15)
C(18)-C(19)-C(14)-C(15)	-0.6(2)
C(13)-C(19)-C(14)-C(15)	170.48(15)
N(1)-C(1)-C(2)-C(3)	-179.60(17)
C(6)-C(1)-C(2)-C(3)	-0.1(3)
C(1)-C(6)-C(5)-C(4)	-0.1(2)
C(7)-C(6)-C(5)-C(4)	178.70(17)
C(18)-C(17)-C(16)-C(15)	0.2(3)
C(17)-C(16)-C(15)-C(14)	0.5(3)
C(19)-C(14)-C(15)-C(16)	-0.3(3)
C(6)-C(5)-C(4)-C(3)	-0.5(3)
C(1)-C(2)-C(3)-C(4)	-0.5(3)
C(5)-C(4)-C(3)-C(2)	0.8(3)

Symmetry transformations used to generate equivalent atoms:

S 25. Crystal data and structure refinement for 11g.

Identification code	1_a	
Empirical formula	C19 H17 N3 O3	
Formula weight	335.35	
Temperature	296(2) K	
Wavelength	0.71073 Å	
Crystal system	Monoclinic	
Space group	$P2_1/c$	
Unit cell dimensions	a = 15.447(3) Å	α= 90°.
	b = 9.462(2) Å	β= 110.467(4)°.
	c = 12.184(3) Å	γ = 90°.
Volume	1668.5(6) $Å^3$	
Z	4	
Density (calculated)	1.335 Mg/m^3	
Absorption coefficient	0.092 mm^{-1}	
F(000)	704	
Crystal size	0.300 x 0.200 x 0.200 mm^3	
Theta range for data collection	1.407 to 31.304°.	
Index ranges	-20<=h<=19, -12<=k<=13, -13<=l<=17	
Reflections collected	11273	
Independent reflections	4785 [R(int) = 0.0369]	
Completeness to theta = 25.242°	99.9 %	
Absorption correction	Semi-empirical from equivalents	
Refinement method	Full-matrix least-squares on F^2	
Data / restraints / parameters	4785 / 0 / 227	
Goodness-of-fit on F^2	0.999	
Final R indices [I>2sigma(I)]	R1 = 0.0518, wR2 = 0.1245	
R indices (all data)	R1 = 0.1129, wR2 = 0.1527	
Extinction coefficient	n/a	
Largest diff. peak and hole	0.138 and -0.209 e.$Å^{-3}$	

S 26. Atomic coordinates (x 10^4) and equivalent isotropic displacement parameters (Å^{2}x 10^3) for 11g. U(eq) is defined as one third of the trace of the orthogonalized U^{ij} tensor.

	x	y	z	U(eq)
C(1)	2219(1)	2741(2)	5184(1)	47(1)
C(2)	2860(1)	1811(2)	5932(2)	60(1)
C(3)	1649(2)	78(2)	5621(2)	71(1)
C(4)	2562(2)	490(2)	6146(2)	72(1)
C(5)	1004(2)	954(2)	4867(2)	61(1)
C(6)	1295(1)	2286(2)	4664(2)	48(1)
C(7)	1384(1)	4492(2)	4074(1)	42(1)
C(8)	2253(1)	4154(2)	4784(1)	45(1)
C(9)	3017(1)	5218(2)	5086(2)	53(1)
C(10)	2583(1)	6678(2)	4915(1)	48(1)
C(11)	1841(1)	7828(2)	2968(1)	41(1)
C(12)	2656(1)	8795(2)	3186(1)	42(1)
C(13)	2498(1)	10230(2)	3192(2)	51(1)
C(14)	3205(1)	11183(2)	3349(2)	58(1)
C(15)	4061(1)	10674(2)	3458(2)	56(1)
C(16)	4237(1)	9260(2)	3419(2)	62(1)
C(17)	3529(1)	8311(2)	3296(2)	55(1)
C(18)	1052(1)	5879(2)	3491(1)	43(1)
C(19)	312(1)	6546(2)	3889(2)	57(1)
N(1)	798(1)	3368(2)	3981(1)	48(1)
N(2)	1869(1)	6807(1)	3741(1)	42(1)
N(3)	4811(1)	11698(3)	3588(2)	81(1)
O(1)	4716(1)	12895(2)	3865(2)	124(1)
O(2)	5490(2)	11289(3)	3430(2)	137(1)
O(3)	1165(1)	8009(1)	2062(1)	53(1)

S 27. Bond lengths [Å] and angles [°] for 11g.

C(1)-C(2)	1.398(2)
C(1)-C(6)	1.412(2)
C(1)-C(8)	1.430(2)
C(2)-C(4)	1.387(3)
C(2)-H(2)	0.9300
C(3)-C(5)	1.373(3)
C(3)-C(4)	1.386(3)
C(3)-H(3)	0.9300
C(4)-H(4)	0.9300
C(5)-C(6)	1.389(2)
C(5)-H(5)	0.9300
C(6)-N(1)	1.371(2)
C(7)-C(8)	1.356(2)
C(7)-N(1)	1.375(2)
C(7)-C(18)	1.496(2)
C(8)-C(9)	1.497(2)
C(9)-C(10)	1.517(3)
C(9)-H(9A)	0.9700
C(9)-H(9B)	0.9700
C(10)-N(2)	1.474(2)
C(10)-H(10A)	0.9700
C(10)-H(10B)	0.9700
C(11)-O(3)	1.2374(19)
C(11)-N(2)	1.339(2)
C(11)-C(12)	1.504(2)
C(12)-C(13)	1.380(2)
C(12)-C(17)	1.384(2)
C(13)-C(14)	1.377(2)
C(13)-H(13)	0.9300
C(14)-C(15)	1.369(3)
C(14)-H(14)	0.9300
C(15)-C(16)	1.369(3)
C(15)-N(3)	1.475(2)
C(16)-C(17)	1.383(3)

C(16)-H(16)	0.9300
C(17)-H(17)	0.9300
C(18)-N(2)	1.4793(19)
C(18)-C(19)	1.526(2)
C(18)-H(18)	0.9800
C(19)-H(19A)	0.9600
C(19)-H(19B)	0.9600
C(19)-H(19C)	0.9600
N(1)-H(1)	0.8600
N(3)-O(2)	1.195(3)
N(3)-O(1)	1.205(3)

C(2)-C(1)-C(6)	118.36(17)
C(2)-C(1)-C(8)	135.14(17)
C(6)-C(1)-C(8)	106.49(15)
C(4)-C(2)-C(1)	119.0(2)
C(4)-C(2)-H(2)	120.5
C(1)-C(2)-H(2)	120.5
C(5)-C(3)-C(4)	121.5(2)
C(5)-C(3)-H(3)	119.3
C(4)-C(3)-H(3)	119.3
C(3)-C(4)-C(2)	121.2(2)
C(3)-C(4)-H(4)	119.4
C(2)-C(4)-H(4)	119.4
C(3)-C(5)-C(6)	117.63(19)
C(3)-C(5)-H(5)	121.2
C(6)-C(5)-H(5)	121.2
N(1)-C(6)-C(5)	129.66(17)
N(1)-C(6)-C(1)	107.95(15)
C(5)-C(6)-C(1)	122.40(17)
C(8)-C(7)-N(1)	110.27(15)
C(8)-C(7)-C(18)	127.42(15)
N(1)-C(7)-C(18)	122.26(14)
C(7)-C(8)-C(1)	106.94(14)
C(7)-C(8)-C(9)	121.23(15)
C(1)-C(8)-C(9)	131.66(15)

C(8)-C(9)-C(10)	107.81(14)
C(8)-C(9)-H(9A)	110.1
C(10)-C(9)-H(9A)	110.1
C(8)-C(9)-H(9B)	110.1
C(10)-C(9)-H(9B)	110.1
H(9A)-C(9)-H(9B)	108.5
N(2)-C(10)-C(9)	110.74(13)
N(2)-C(10)-H(10A)	109.5
C(9)-C(10)-H(10A)	109.5
N(2)-C(10)-H(10B)	109.5
C(9)-C(10)-H(10B)	109.5
H(10A)-C(10)-H(10B)	108.1
O(3)-C(11)-N(2)	122.49(15)
O(3)-C(11)-C(12)	117.82(14)
N(2)-C(11)-C(12)	119.68(14)
C(13)-C(12)-C(17)	119.52(16)
C(13)-C(12)-C(11)	117.42(15)
C(17)-C(12)-C(11)	122.90(16)
C(14)-C(13)-C(12)	120.80(17)
C(14)-C(13)-H(13)	119.6
C(12)-C(13)-H(13)	119.6
C(15)-C(14)-C(13)	118.38(18)
C(15)-C(14)-H(14)	120.8
C(13)-C(14)-H(14)	120.8
C(14)-C(15)-C(16)	122.44(17)
C(14)-C(15)-N(3)	118.4(2)
C(16)-C(15)-N(3)	119.2(2)
C(15)-C(16)-C(17)	118.69(18)
C(15)-C(16)-H(16)	120.7
C(17)-C(16)-H(16)	120.7
C(16)-C(17)-C(12)	120.11(18)
C(16)-C(17)-H(17)	119.9
C(12)-C(17)-H(17)	119.9
N(2)-C(18)-C(7)	107.31(12)
N(2)-C(18)-C(19)	111.66(14)
C(7)-C(18)-C(19)	112.44(14)

N(2)-C(18)-H(18)	108.4
C(7)-C(18)-H(18)	108.4
C(19)-C(18)-H(18)	108.4
C(18)-C(19)-H(19A)	109.5
C(18)-C(19)-H(19B)	109.5
H(19A)-C(19)-H(19B)	109.5
C(18)-C(19)-H(19C)	109.5
H(19A)-C(19)-H(19C)	109.5
H(19B)-C(19)-H(19C)	109.5
C(6)-N(1)-C(7)	108.34(14)
C(6)-N(1)-H(1)	125.8
C(7)-N(1)-H(1)	125.8
C(11)-N(2)-C(10)	124.85(13)
C(11)-N(2)-C(18)	117.86(13)
C(10)-N(2)-C(18)	116.48(13)
O(2)-N(3)-O(1)	123.2(2)
O(2)-N(3)-C(15)	118.1(2)
O(1)-N(3)-C(15)	118.7(2)

Symmetry transformations used to generate equivalent atoms:

S 28. Anisotropic displacement parameters ($Å^2 x 10^3$) for 11g. The anisotropic displacement factor exponent takes the form: $-2\pi^2[h^2 a^{*2}U^{11} + ... + 2 h k a^* b^* U^{12}]$

	U^{11}	U^{22}	U^{33}	U^{23}	U^{13}	U^{12}
C(1)	54(1)	43(1)	45(1)	-2(1)	16(1)	3(1)
C(2)	66(1)	54(1)	55(1)	1(1)	15(1)	10(1)
C(3)	98(2)	46(1)	82(2)	8(1)	46(1)	2(1)
C(4)	97(2)	52(1)	66(1)	14(1)	28(1)	21(1)
C(5)	69(1)	50(1)	72(1)	4(1)	35(1)	-6(1)
C(6)	56(1)	42(1)	50(1)	0(1)	23(1)	-2(1)
C(7)	44(1)	40(1)	42(1)	-1(1)	15(1)	-4(1)
C(8)	44(1)	40(1)	46(1)	-2(1)	11(1)	-1(1)
C(9)	46(1)	51(1)	50(1)	2(1)	3(1)	-4(1)
C(10)	52(1)	46(1)	39(1)	-1(1)	6(1)	-9(1)
C(11)	41(1)	41(1)	42(1)	-2(1)	15(1)	1(1)
C(12)	44(1)	45(1)	37(1)	1(1)	15(1)	-2(1)
C(13)	51(1)	47(1)	60(1)	1(1)	26(1)	-1(1)
C(14)	70(1)	47(1)	60(1)	-1(1)	27(1)	-9(1)
C(15)	52(1)	66(1)	47(1)	1(1)	15(1)	-19(1)
C(16)	40(1)	76(2)	70(1)	2(1)	18(1)	-2(1)
C(17)	48(1)	53(1)	65(1)	2(1)	20(1)	1(1)
C(18)	40(1)	44(1)	42(1)	0(1)	12(1)	-6(1)
C(19)	52(1)	56(1)	67(1)	8(1)	26(1)	6(1)
N(1)	44(1)	45(1)	54(1)	3(1)	15(1)	-6(1)
N(2)	41(1)	42(1)	39(1)	1(1)	8(1)	-7(1)
N(3)	66(1)	94(2)	76(1)	7(1)	14(1)	-31(1)
O(1)	113(2)	82(1)	165(2)	-9(1)	34(1)	-49(1)
O(2)	81(1)	148(2)	200(2)	-10(2)	71(2)	-43(1)
O(3)	46(1)	59(1)	48(1)	10(1)	7(1)	-2(1)

S 29. Hydrogen coordinates (x 10^4) and isotropic displacement parameters ($Å^2$x 10^3) for 11g.

	x	y	z	U(eq)
H(2)	3476	2074	6281	72
H(3)	1469	-813	5783	85
H(4)	2983	-129	6651	86
H(5)	395	667	4505	73
H(9A)	3379	5097	4583	64
H(9B)	3422	5098	5893	64
H(10A)	2308	6846	5506	58
H(10B)	3057	7386	5007	58
H(13)	1908	10557	3089	61
H(14)	3104	12148	3380	70
H(16)	4821	8945	3475	75
H(17)	3638	7345	3288	66
H(18)	789	5723	2643	51
H(19A)	559	6703	4719	86
H(19B)	-210	5923	3705	86
H(19C)	122	7431	3493	86
H(1)	219	3346	3566	58

S 30. Torsion angles [°] for 11g.

C(6)-C(1)-C(2)-C(4)	-0.8(3)
C(8)-C(1)-C(2)-C(4)	178.20(19)
C(5)-C(3)-C(4)-C(2)	0.2(3)
C(1)-C(2)-C(4)-C(3)	0.8(3)
C(4)-C(3)-C(5)-C(6)	-1.2(3)
C(3)-C(5)-C(6)-N(1)	-178.51(19)
C(3)-C(5)-C(6)-C(1)	1.2(3)
C(2)-C(1)-C(6)-N(1)	179.55(15)
C(8)-C(1)-C(6)-N(1)	0.30(19)
C(2)-C(1)-C(6)-C(5)	-0.2(3)
C(8)-C(1)-C(6)-C(5)	-179.48(16)
N(1)-C(7)-C(8)-C(1)	-0.99(19)
C(18)-C(7)-C(8)-C(1)	176.55(16)
N(1)-C(7)-C(8)-C(9)	-176.69(15)
C(18)-C(7)-C(8)-C(9)	0.8(3)
C(2)-C(1)-C(8)-C(7)	-178.7(2)
C(6)-C(1)-C(8)-C(7)	0.41(19)
C(2)-C(1)-C(8)-C(9)	-3.6(3)
C(6)-C(1)-C(8)-C(9)	175.50(18)
C(7)-C(8)-C(9)-C(10)	22.6(2)
C(1)-C(8)-C(9)-C(10)	-151.93(18)
C(8)-C(9)-C(10)-N(2)	-51.88(19)
O(3)-C(11)-C(12)-C(13)	55.7(2)
N(2)-C(11)-C(12)-C(13)	-124.84(18)
O(3)-C(11)-C(12)-C(17)	-119.69(19)
N(2)-C(11)-C(12)-C(17)	59.7(2)
C(17)-C(12)-C(13)-C(14)	-2.4(3)
C(11)-C(12)-C(13)-C(14)	-177.95(16)
C(12)-C(13)-C(14)-C(15)	2.2(3)
C(13)-C(14)-C(15)-C(16)	-0.2(3)
C(13)-C(14)-C(15)-N(3)	178.08(17)
C(14)-C(15)-C(16)-C(17)	-1.7(3)
N(3)-C(15)-C(16)-C(17)	-179.95(17)
C(15)-C(16)-C(17)-C(12)	1.6(3)

C(13)-C(12)-C(17)-C(16)	0.4(3)
C(11)-C(12)-C(17)-C(16)	175.76(17)
C(8)-C(7)-C(18)-N(2)	4.9(2)
N(1)-C(7)-C(18)-N(2)	-177.81(13)
C(8)-C(7)-C(18)-C(19)	-118.23(19)
N(1)-C(7)-C(18)-C(19)	59.0(2)
C(5)-C(6)-N(1)-C(7)	178.86(18)
C(1)-C(6)-N(1)-C(7)	-0.90(19)
C(8)-C(7)-N(1)-C(6)	1.20(19)
C(18)-C(7)-N(1)-C(6)	-176.49(15)
O(3)-C(11)-N(2)-C(10)	-169.82(16)
C(12)-C(11)-N(2)-C(10)	10.8(2)
O(3)-C(11)-N(2)-C(18)	-0.5(2)
C(12)-C(11)-N(2)-C(18)	-179.88(14)
C(9)-C(10)-N(2)-C(11)	-126.44(17)
C(9)-C(10)-N(2)-C(18)	64.08(19)
C(7)-C(18)-N(2)-C(11)	152.75(14)
C(19)-C(18)-N(2)-C(11)	-83.61(18)
C(7)-C(18)-N(2)-C(10)	-37.02(18)
C(19)-C(18)-N(2)-C(10)	86.63(17)
C(14)-C(15)-N(3)-O(2)	-164.2(2)
C(16)-C(15)-N(3)-O(2)	14.2(3)
C(14)-C(15)-N(3)-O(1)	17.0(3)
C(16)-C(15)-N(3)-O(1)	-164.7(2)

Symmetry transformations used to generate equivalent atoms:
